# A Cold Case: Myxedema Coma

**DOI:** 10.21980/J8VM0J

**Published:** 2025-01-31

**Authors:** Andrew M Namespetra, Matthew J Petruso, Andrew M Bazakis

**Affiliations:** * Central Michigan University College of Medicine, Department of Emergency Medicine, Saginaw, MI

## Abstract

**Audience:**

This case was designed and developed to train emergency medicine residents through high-fidelity simulation and experiential learning in the management of a hemodynamically unstable patient presenting with myxedema coma.

**Introduction:**

Myxedema coma refers to decompensated hypothyroidism manifesting as altered mental status and multisystem organ dysfunction. Myxedema coma is a life-threatening endocrine emergency that requires prompt recognition and treatment. Mortality associated with this condition is high, approaching 30% with optimized treatment, and nearly 100% if untreated.^1^ Whilst myxedema coma is a cannot-miss diagnosis, it is a relatively uncommon presentation to the emergency department (ED); incidence of myxedema coma is as low as 1.08 per million people per year.^2^ The clinical triad of myxedema coma is altered mental status, hypothermia and the presence of a precipitating factor.^3^ Typically, the patient will be over age 60 years, female, and with clinical features associated with hypothyroidism including dry skin, coarse hair, non-pitting edema.^4^ Myxedema coma has a temporal association with most cases occurring in the winter months.^5^ Despite knowledge of the disease process, recognition can be challenging, thus delaying treatment. Therefore, clinicians must have a high degree of suspicion to make the diagnosis in the ED. These characteristics of infrequency and lethality suggest medical simulation as an ideal medium to educate learners on recognition, diagnosis and management of myxedema coma in the ED in a realistic and safe setting.

**Educational Objectives:**

The primary educational goals are to elicit the differential diagnoses for a patient with altered mental status, order an appropriate workup, and initiate life-saving interventions for a patient with decompensated hypothyroidism. At the conclusion of the simulation, the learner is expected to: 1) Recognize the key features on history and examination of a patient presenting in myxedema coma and initiate the appropriate workup and treatment, 2) Describe clinical features and management for a patient with myxedema coma, 3) Develop a differential diagnosis for a critically ill patient with altered mental status, 4) Discuss the management of myxedema coma in the ED, including treatments, appropriate consultation, and disposition.

**Educational Methods:**

This case was delivered as a high-fidelity simulation employing a computerized manikin as the patient, and a confederate actor in the role of the registered nurse (RN). A post-scenario debriefing session was facilitated by the instructor as a four-step formative process described by Rudolph, *et al.*^6^ Other aspects of the debriefing included discussion about the pathophysiology, presentation, management, and disposition of patients with myxedema coma.

**Research Methods:**

Learners were asked to submit anonymous feedback immediately upon completion of the case. Objective data from learners was obtained ranging from 4th year medical students on their Emergency Medicine (EM) clerkship rotation at one clinical site to PGY1–4 EM residents from two residency programs, both experiencing the same simulation at the same site. The post-simulation survey was the same for all learners. Drop-down lists were used when asking the level of training and how many cases of myxedema the learner had seen. The rest of the learner feedback was assessed with a 5-point Likert scale (1: strongly disagree to 5: strongly agree). Anonymous open-ended comments were available for narrative feedback.

**Results:**

Thirty-three learners completed the post-simulation surveys. Learners rated the effectiveness of the simulation very highly with an average score of 4.6/5 on the Likert scale. Most learners endorsed supporting the use of the case in their simulation curriculum (average of 4.5/5). Debriefing effectiveness was also rated very highly, (average 4.8/5). As noted, topics of discussion during debriefing included clinical features and pathophysiology of myxedema coma, principles of resuscitation, empiric management of decompensated hypothyroidism, and disposition.

**Discussion:**

The simulation case was an effective and reproducible method of training EM residents in the recognition and management of a relatively rare yet fatal condition: myxedema coma. Learners were challenged to aggressively resuscitate an unstable critically ill patient whilst thinking through many potential diagnoses in a patient with altered mental status. After review of the learner feedback, the simulation and debriefing were regarded as effective and successful in achieving the learning objectives. The quality, accuracy and effectiveness of the educational content is clearly positive as indicated by the overwhelming positive responses. Furthermore, the survey results demonstrate that many residents (60.6%, Figure 1) have never seen a case. This supports the rarity of the condition and highlights the need for simulation to fill the learning gap.

**Topics:**

Medical simulation, emergency medicine, myxedema coma, hypothyroidism, endocrine emergencies, altered mental status, hypoglycemia, hypothermia, bradyarrhythmia.

## USER GUIDE

[Table t2-jetem-10-1-s1]

**Table t2-jetem-10-1-s1:** 

List of Resources:	
Abstract	1
User Guide	3
Instructor Materials	6
Operator Materials	28
Debriefing and Evaluation Pearls	33
Simulation Assessment	38

**Learner Audience:**

4^th^-year Medical Students (on EM clerkship rotation), Interns, Junior EM Residents, Senior EM Residents

**Time Required for Implementation:**

**Instructor Preparation:** 30 minutes

**Time for case:** 15 minutes

**Time for debriefing:** 15–20 minutes

**Recommended Number of Learners per Instructor:**

Four learners per case, ideally from different levels of training.

**Topics:**

Medical simulation, emergency medicine, myxedema coma, hypothyroidism, endocrine emergencies, altered mental status, hypoglycemia, hypothermia, bradyarrhythmia.

**Objectives:**

At the conclusion of this simulation, the learners will be able to:

1. Recognize the key features on history and examination of a patient presenting in myxedema coma and initiate the appropriate workup and treatment
2. Describe clinical features and management for a patient with myxedema coma
3. Develop a differential diagnosis for a critically ill patient with altered mental status
4. Discuss the management of myxedema coma in the ED, including treatments, appropriate consultation and disposition

### Linked objectives and methods

#### Myxedema coma is a rare but potentially fatal condition

The case is designed to present the key historical features and clinical signs associated with myxedema coma to foster learner recognition. The mannequin may be moulaged to demonstrate features such as thinning hair, pretibial myxedema, periorbital edema, dry scaling skin and obesity, which convey the hallmark clinical features associated with myxedema coma (Objective 1). The learners will encounter the classic clinical features of myxedema coma, including hypothermia, refractory bradycardia and hypotension, respiratory depression, hypoglycemia and hyponatremia (Objective 2). Formulating a broad differential diagnosis for a critically ill patient with altered mental status will be encouraged (Objective 3). The abnormal vital signs and clinical deterioration of the patient prompt the learners to initiate rapid resuscitation, including airway management and early vasopressors. Appropriate dosing of thyroid hormone and corticosteroids for treatment of myxedema coma will be discussed. An endocrinologist, role-played by the instructor or other embedded personnel, is available by phone for consultation, and a critical care physician is available to disposition the patient to the ICU (Objective 4). Throughout the simulation, the learners’ performance is tracked by an observing facilitator who will then give formative feedback during debriefing. A standardized debriefing session will reinforce all learning objectives after the simulation is complete.

### Recommended pre-reading for instructor

The instructor is encouraged to review and become familiar with the attached materials, including the patient’s laboratory and imaging results, the simulation flow chart, the simulation PowerPoint, and the references and suggestions for further reading. Other recommended FOAMed resources include:

1. Bridwell R. EM@3AM: Decompensated hypothyroidism. emDOCs.net - Emergency Medicine Education. July 1, 2020. Accessed May 30, 2024. http://www.emdocs.net/em3am-myxedema-coma/
2. Myxedema Coma - Wikem. Accessed May 31, 2024. https://wikem.org/wiki/Myxedema_coma
3. Farkas J. IBCC Chapter & Cast - Myxedema Coma (decompensated hypothyroidism). EMCrit Project. January 2, 2020. Accessed May 30, 2024. https://emcrit.org/pulmcrit/myxedema/
4. Mason J, Herbert M, Swadron S. Myxedema Coma. EM RAP. Accessed May 30, 2024. https://www.emrap.org/episode/c3thyroid/c3thyroid4
5. Thomas A, Farkas J. IBCC episode 71 - Myxedema Coma. The Internet Book of Critical Care Podcast. Accessed May 30, 2024. https://ibccpodcast.libsyn.com/ibcc-episode-71-myxedema-coma

### Results and tips for successful implementation

This case is best implemented as a high-fidelity scenario with a team of learners from a spectrum of levels of training in a well-equipped simulation facility with adequate resources and staffing to play the role of embedded simulation personnel (ESPs) and evaluators to maximize learner buy-in and to provide detailed feedback on learner performance. Alternatively, one may distribute the case in oral board format to a single learner.

**Figure 1:**
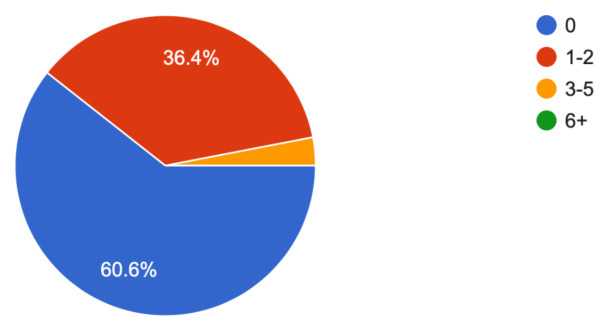
Learner background experience.

**Figure 2:**
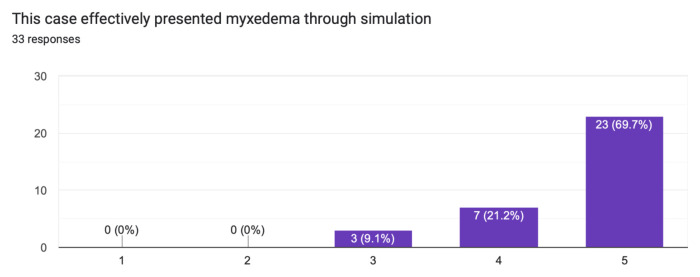
Effectiveness of case representation through simulation.

**Figure 3:**
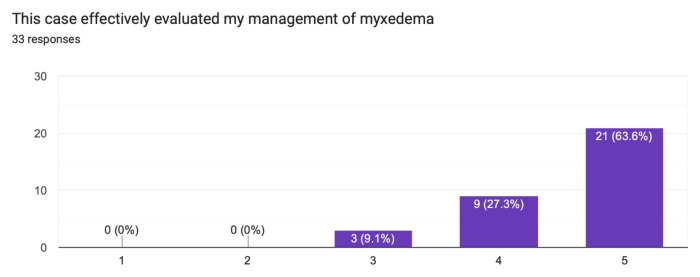
Effectiveness of evaluation through simulation.

**Figure 4:**
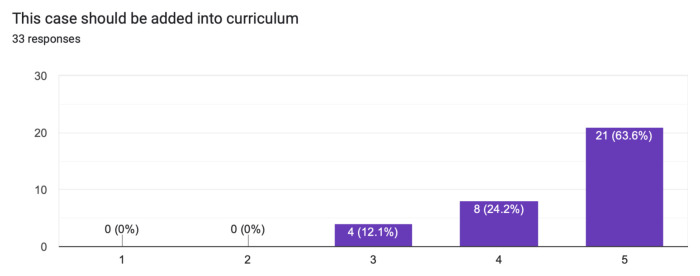
Learner evaluation of suitability of the case in simulation curriculum.

**Figure 5:**
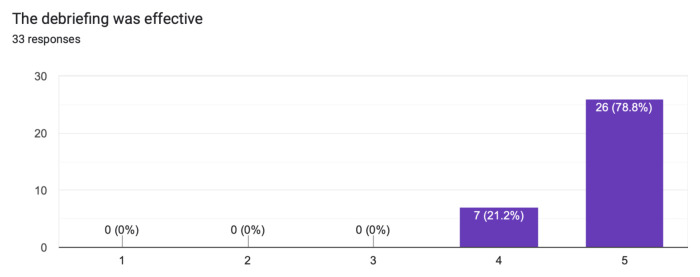
Learner evaluation of the effectiveness of debriefing sessions.

**Figure 6:**
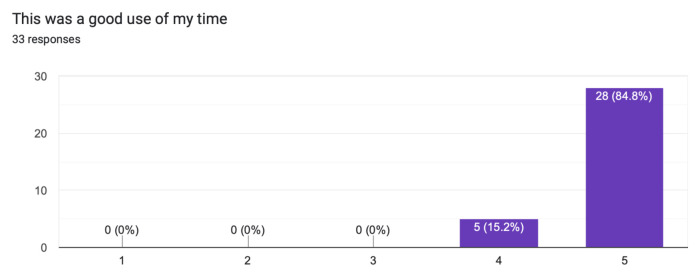
Learner evaluation of general educational value of the simulation.

The case was distributed in a high-fidelity simulation lab to 33 learners from a range of levels of training as indicated in Table 1. The majority of learners (60.6%) had not seen nor treated a patient with myxedema (Figure 1). Effectiveness of the simulation was evaluated by the learners and demonstrated in Figures 2 and 3. The value of the case and suitability for its use in a simulation curriculum is demonstrated in Figure 4. There was strong positive regard toward the effectiveness of the debriefing sessions as depicted in Figure 5. An evaluation of the overall educational value of the simulation experience was evaluated by level of agreement to the statement: “This was a good use of my time,” and the results can be seen in Figure 6.

Learners were given the opportunity to provide narrative feedback regarding their experience. Quotes from learners that demonstrate that the case had appropriate complexity are as follows:

- “This was a great case with many moving parts. Required us to think outside the box.”
- “Excellent case. The blood sugar red herring was great!”
- “Excellent case requiring a broad spectrum of simultaneous management.”
- “Great case, good options for differentials but clear cut and really good that we had to treat empirically. Really good practice. I’ve seen one previously and it was very very similar to this case!”

There were also comments that support the quality of the debriefing session:

- “Excellent debrief”
- “I thought the debriefing went well.”
- “Feedback was very helpful.”

The two highest scoring survey items are quite telling of overall performance:

- “The debriefing was effective,” rated 4.8/5 average on the Likert scale survey, suggesting that the case was designed and executed to convey the learning objectives effectively.
- “This was a good use of my time,” rated 4.5/5 on the Likert scale survey, suggesting educational value as evidenced by the perception of these advanced learners.

### Associated content

A PowerPoint file projected in presentation view format on a large screen in the simulation suite is available to display to the learners during the simulation which contains stimuli such as ECG, diagnostic imaging, and laboratory results. Following the stimuli, there are also slides that guide the debriefing session.

### Ethics

Institutional Review Board (IRB) approval was obtained through the “blinded for peer review.”

## Supplementary Information



## Figures and Tables

**Figure f7-jetem-10-1-s1:**
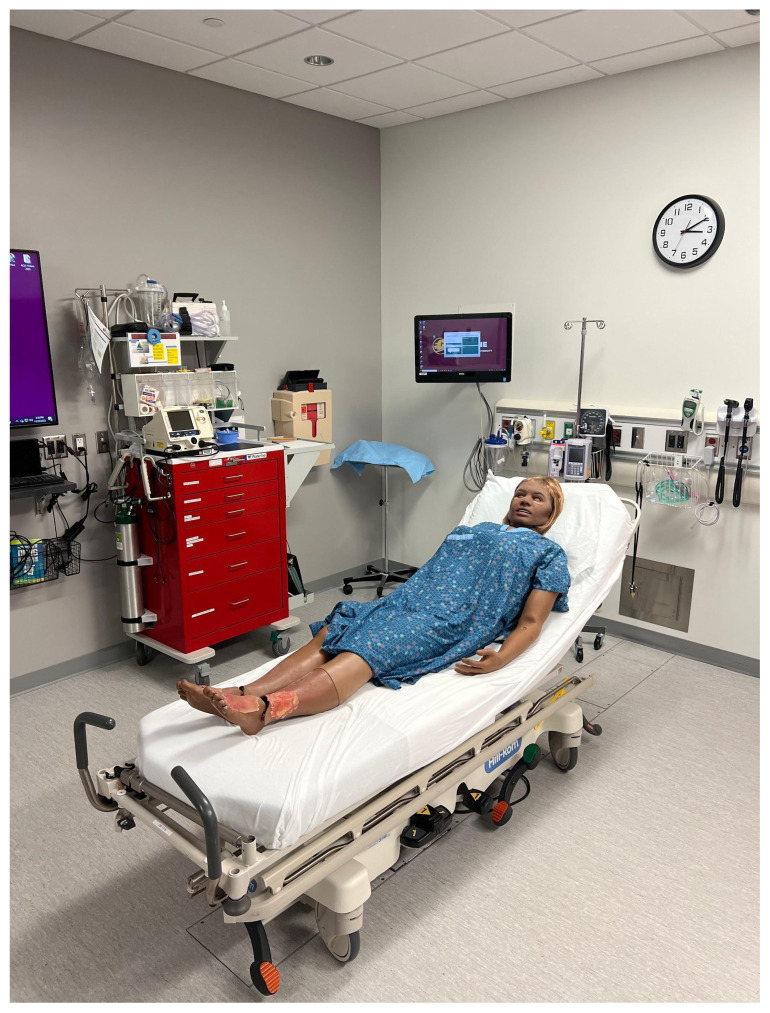
Suggested moulage and simulation set up.

**Figure f8-jetem-10-1-s1:**
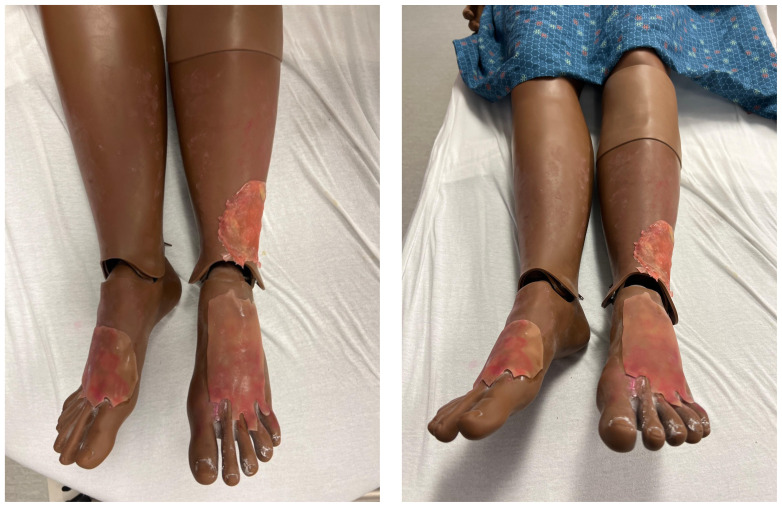
Suggested moulage demonstrating myxedema and scaling skin.

**Figure f9-jetem-10-1-s1:**
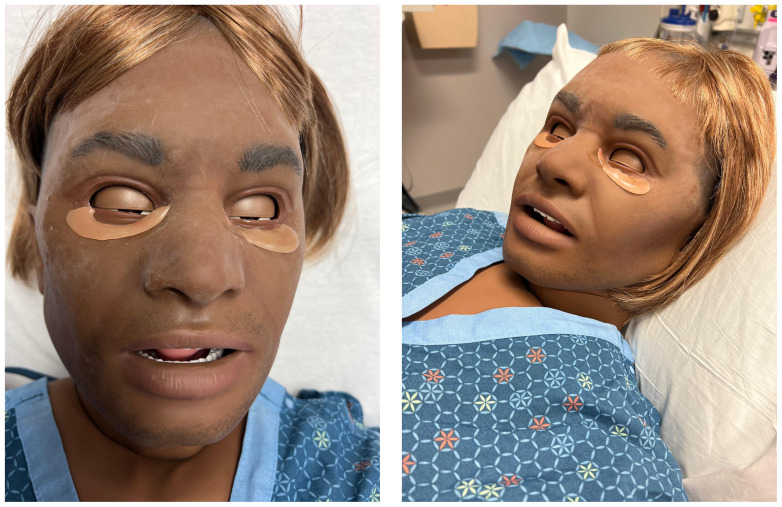
Suggested moulage demonstrating periorbital edema and thinning hair.

**Table t3-jetem-10-1-s1:** 

#1	ECG - Sinus bradycardia
#2	Suggested moulage
#3	Chest X-ray (CXR)
#4	Computed tomography (CT) head
#5	Echocardiography - Global hypokinesia
#6	Arterial blood gas (ABG)
#7	Complete blood count (CBC)
#8	Basic metabolic panel (BMP)
#9	Liver function tests (LFT)
#10	Ethanol level
#11	C-reactive protein (CRP) and Procalcitonin
#12	Thyroid studies
#13	Cortisol level
#14	Ammonia level
#15	Coagulation profile
#16	Osmolality
#17	Lactate
#18	Urinalysis
#19	Urine Drug Screen
#20	Cardiac enzymes
#21	ABG (post-intubation)
#22	Post-intubation CXR

**Table t4-jetem-10-1-s1:** 

**Vitals:**	Heart Rate (HR) 52	Blood Pressure (BP) 95/68
	Respiratory Rate (RR) 12	Temperature (T) 34.1°C
	Oxygen Saturation (O_2_Sat) 85% on room air	

**Figure f10-jetem-10-1-s1:**
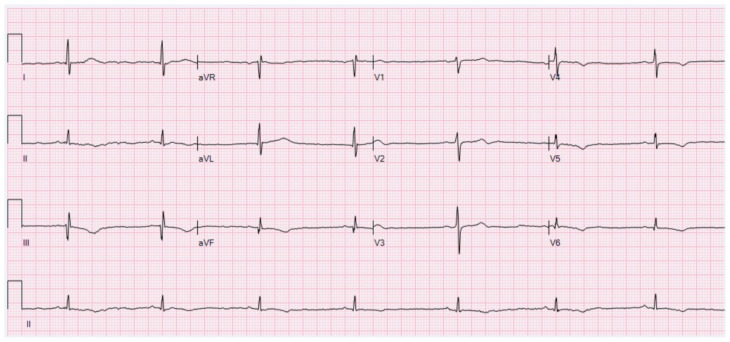


**Figure f11-jetem-10-1-s1:**
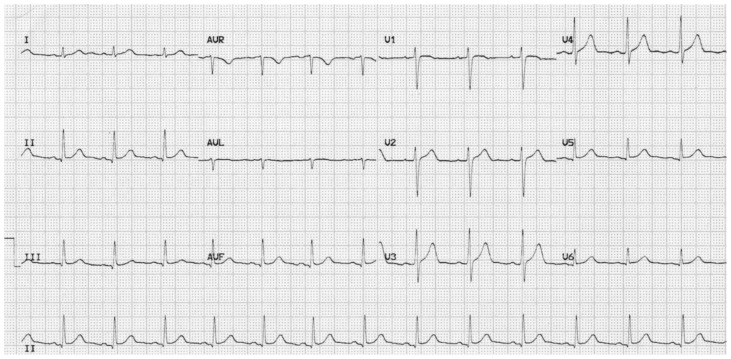


**Figure f12-jetem-10-1-s1:**
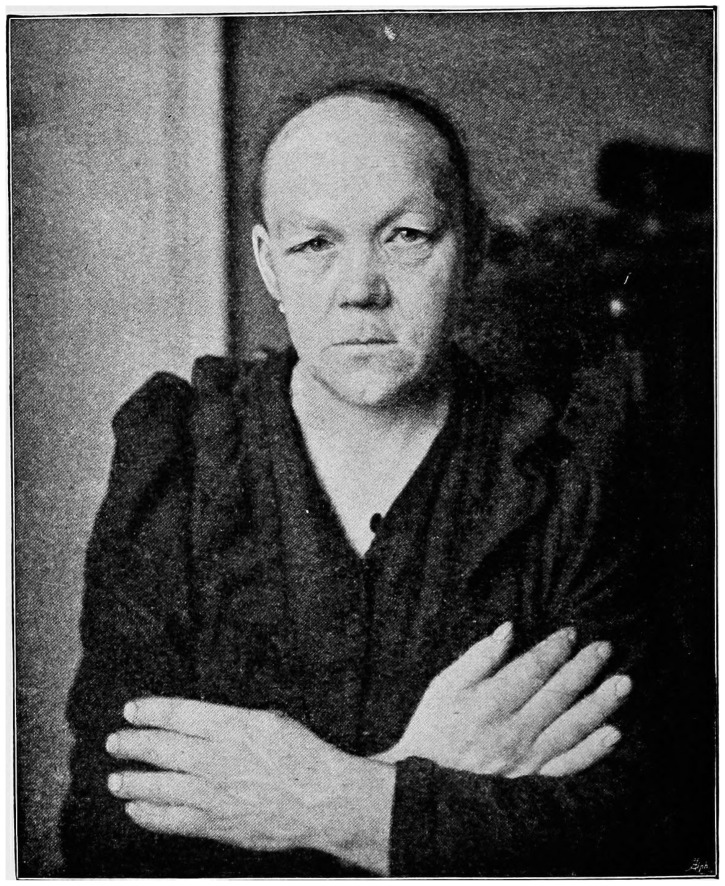


**Figure f13-jetem-10-1-s1:**
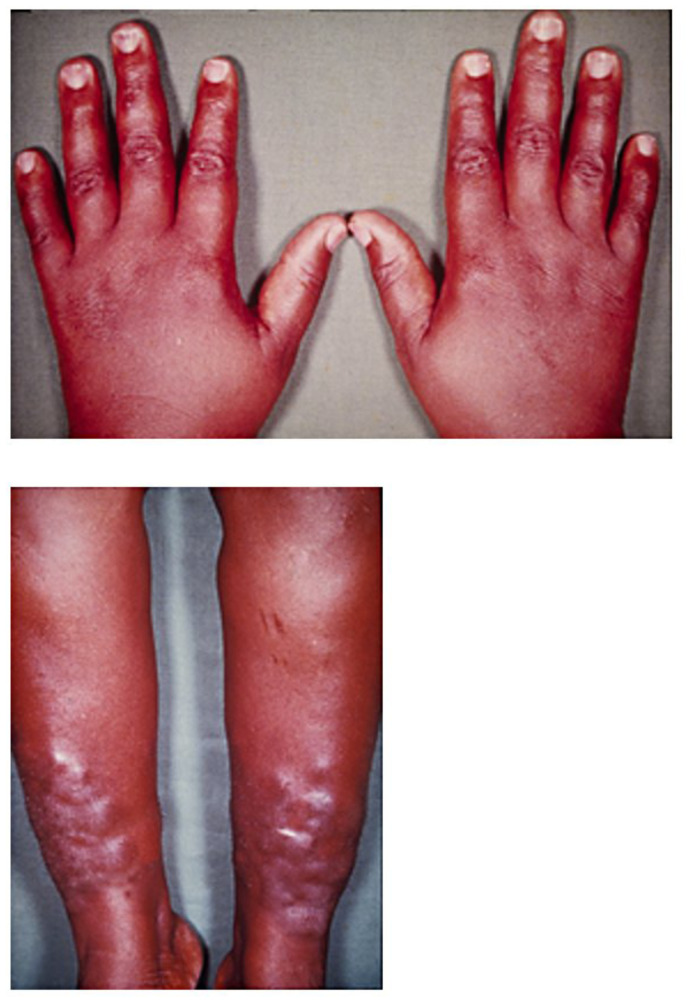


**Figure f14-jetem-10-1-s1:**
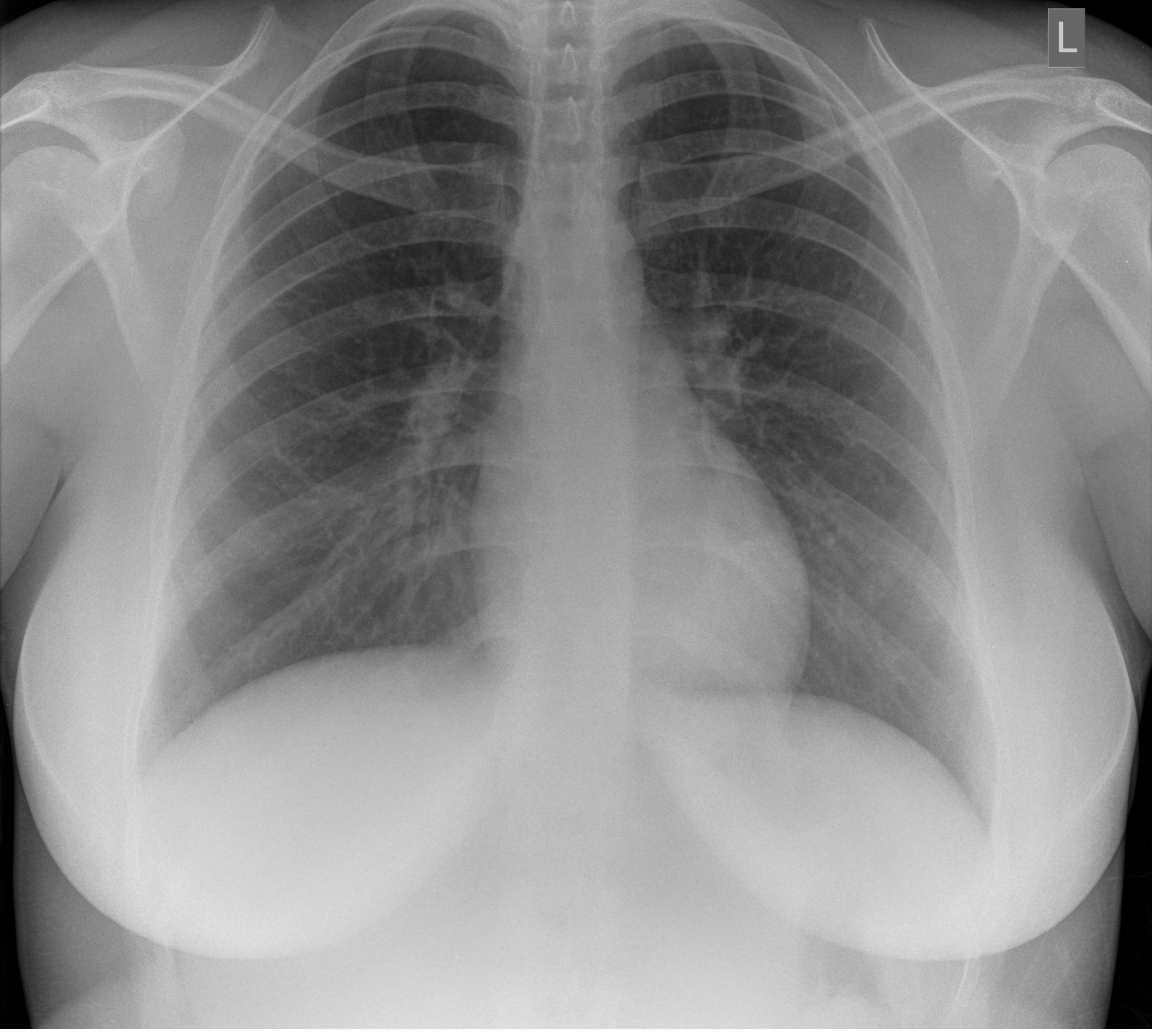


**Figure f15-jetem-10-1-s1:**
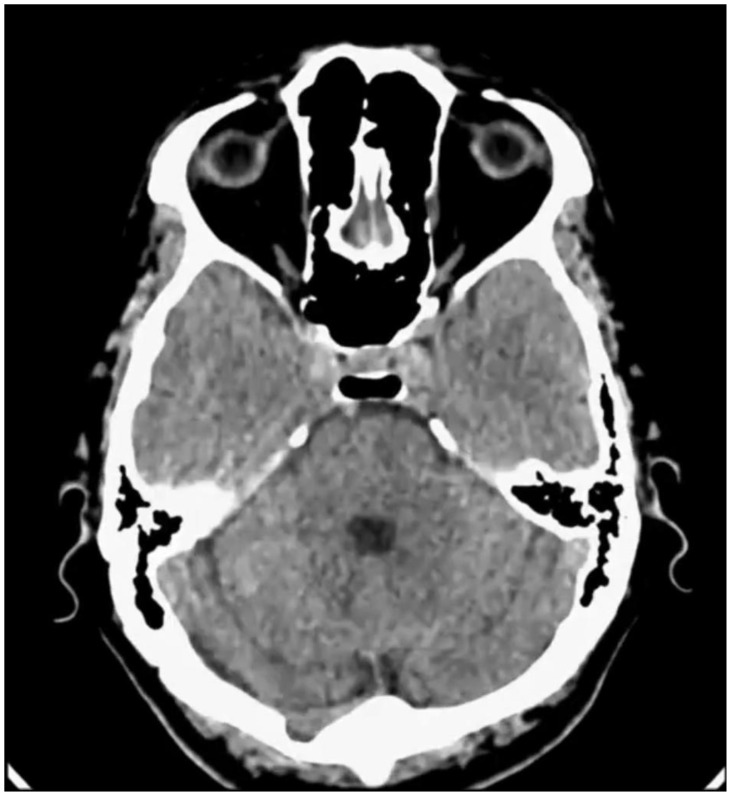


**Figure f16-jetem-10-1-s1:**
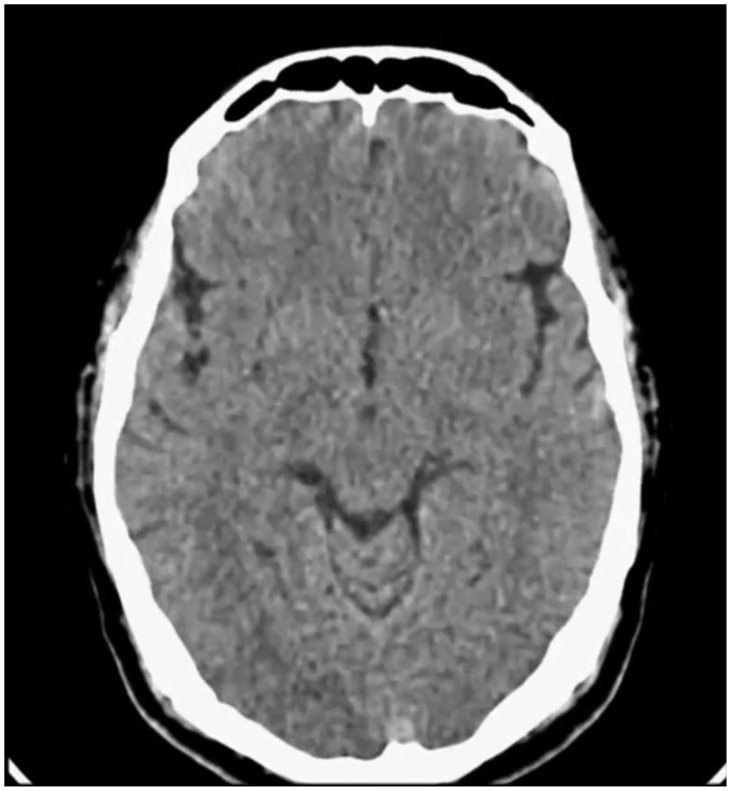


**Figure f17-jetem-10-1-s1:**
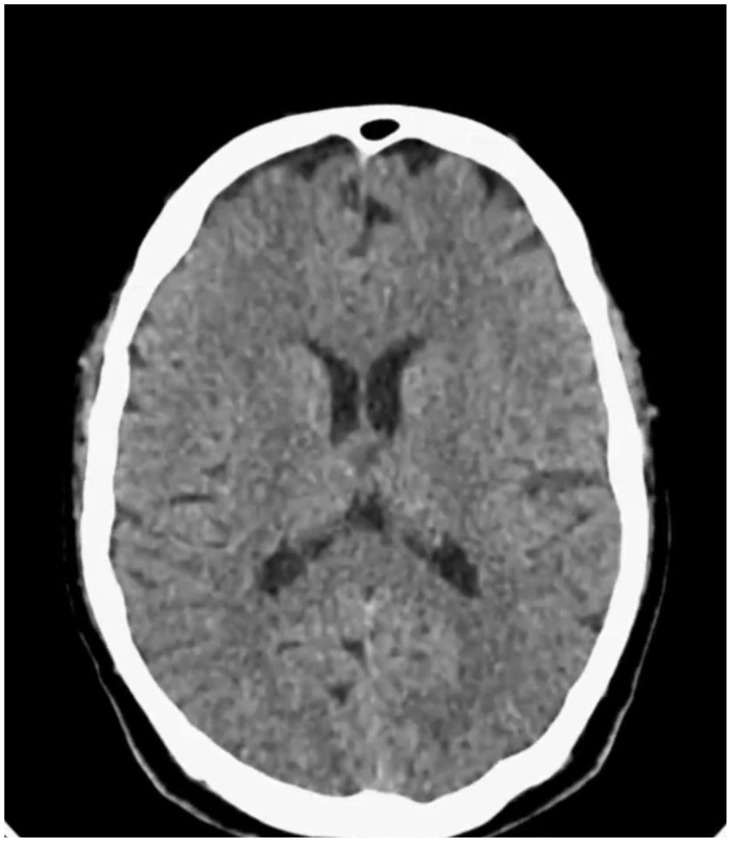


**Figure f18-jetem-10-1-s1:**
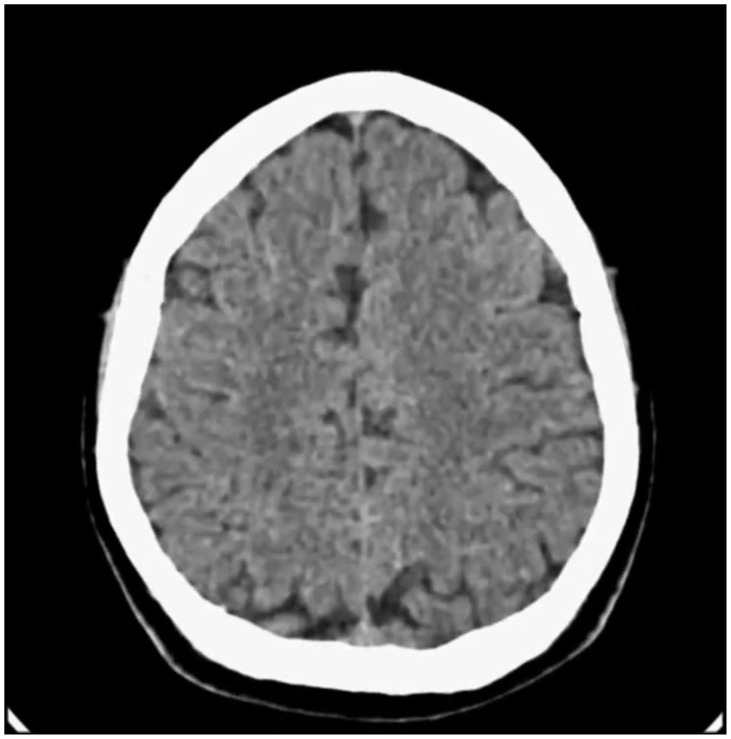


**Table t5-jetem-10-1-s1:** 

*Arterial blood gas (ABG), pre-intubation*	
pH	7.2
paCO2	62 mmHg
paO2	75 mmHg
HCO3	20 mmol/L
SaO2	85%

**Table t6-jetem-10-1-s1:** 

*CBC (Complete Blood Count)*	
White blood count (WBC)	10.0 × 1000/mm^3^
Hemoglobin (Hgb)	11.0 g/dL
Hematocrit (HCT)	33%
Platelet (Plt)	200 × 1000/mm^3^
Mean corpuscular volume (MCV)	80 fL
Mean corpuscular hemoglobin (MCH)	30 pg
Mean corpuscular hemoglobin concentration (MCHC)	32 g/dL

**Table t7-jetem-10-1-s1:** 

*Basic metabolic panel (BMP)*	
Sodium	129 mEq/L
Potassium	4.9 mEq/L
Chloride	100 mEq/L
Bicarbonate (HCO3)	20 mEq/L
Blood Urea Nitrogen (BUN)	20 mg/dL
Creatinine (Cr)	1.2 mg/dL
Glucose	50 mg/dL
Calcium	8.0 mg/dL

**Table t8-jetem-10-1-s1:** 

*Liver Function Tests (LFTs)*	
Total bilirubin	0.9 mg/dL
Direct bilirubin	0.4 mg/dL
Alkaline phosphatase	60 units/L
Aspartate aminotransferase (AST)	40 units/L
Alanine aminotransferase (ALT)	40 units/L

**Table t9-jetem-10-1-s1:** 

*Ethanol (EtOH)*	0.0 mg/dL

**Table t10-jetem-10-1-s1:** 

*Inflammatory markers*	
CRP	3.0 mg/dL
Procalcitonin	0.5 ng/mL

**Table t11-jetem-10-1-s1:** 

*Thyroid Function Tests (TFTs)*	
Thyroid stimulating hormone (TSH)	89.9 mIU/L
Free T4 (fT4)	<0.1 ng/dL
total T3	<1 ng/dL

**Table t12-jetem-10-1-s1:** 

*Cortisol*	10 mcg/dL

**Table t13-jetem-10-1-s1:** 

*Ammonia*	30 mmol/L

**Table t14-jetem-10-1-s1:** 

*Coagulation Profile*	
Prothrombin Time (PT)	12.0 seconds
International Normalized Ratio (INR)	1.0
Partial Thromboplastin Time (PTT)	25.0 seconds

**Table t15-jetem-10-1-s1:** 

*Osmolality*	290

**Table t16-jetem-10-1-s1:** 

*Lactic acid*	4.2 mmol/L

**Table t17-jetem-10-1-s1:** 

*Urinalysis (catheterized)*	
Specific Gravity	1.001
pH	7.0
Protein	5.0
Ketones positive	
Urobilinogen	1.0
Leukocyte esterase	negative
Nitrites	negative
Blood	negative
RBCs	0
WBCs	0
Epithelial cells	none
Bacteria	none
Casts	none
Crystals	none

**Table t18-jetem-10-1-s1:** 

*Urine Drug Screen*	
Amphetamines	negative
Barbiturate	negative
Benzodiazepine	negative
Cannabinoids	negative
Cocaine	negative
Opiates	negative
Methadone	negative
PCP	negative

**Table t19-jetem-10-1-s1:** 

*Cardiac enzymes*	
Troponin	0.01 ng/mL (<0.1 ng/mL)
Creatine kinase (CK)	500 IU/L
CK-MB	1 ng/mL

**Figure f19-jetem-10-1-s1:**
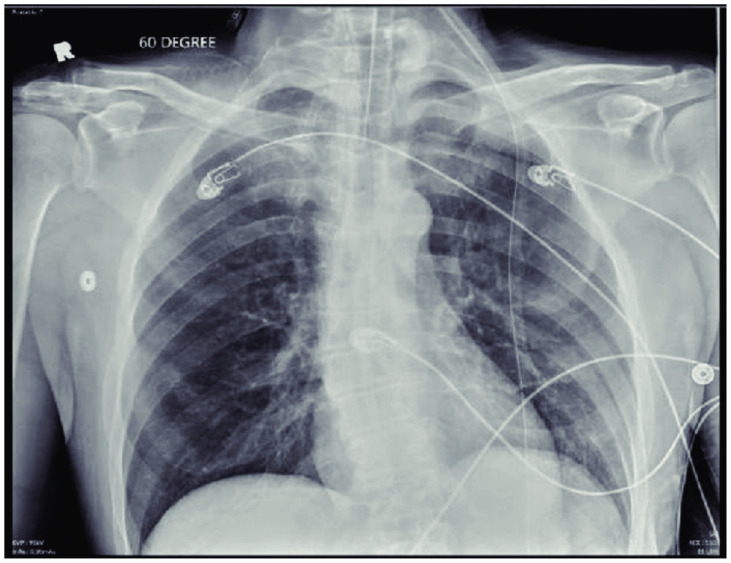


**Table t20-jetem-10-1-s1:** SIMULATION EVENTS TABLE:

Minute (state)	Participant action/ trigger	Patient status (simulator response) & operator prompts	Monitor display (vital signs)
0:00 (Baseline)	Learners enter the room, don PPE and should introduce themselves to the patient, RN and EMS.	The patient is lying in the bed and making moaning noises. Learners should assess ABCs, acquire vital signs including POC blood glucose and obtain a focused history, including medications and history of hypothyroidism. Physical exam findings are consistent with hypothyroidism.	T: 34.1° C (rectal) HR: 52 BP: 95/68 RR: 12 SaO2: 85% on RA Glucose: 55 Glasgow Coma Scale (GCS): 11 (E2 V4 M5)
4:00	Learners should recognize and intervene on abnormal vital signs.	Airway: Administer supplemental O2 Breathing: Consider noninvasive positive pressure ventilation (NIPPV) Circulation: Give IV fluid bolus. Consider addressing bradyarrhythmia with atropine or transcutaneous pacing. Vasopressors. Active or passive rewarming. Disability: Give IV dextrose (D50). Exposure: Address hypothermia with passive rewarming techniques eg, warm blankets. Initiate IV antibiotics for sepsis. Initial investigations including lab studies (CBC, BMP, LFT, Lactate, VBG, Blood cultures, TSH, Tox screen, Troponin, UA), 12-lead ECG, CXR, bedside Echocardiogram.	T: 34.9° C HR: 45 BP: 85/50 RR: 10 SaO2: 90% on O2 (if no O2 given, drop to 75%) Glucose: 80 (if D50 is given) GCS: 8 (E1 V3 M4)
6:00	If ABCDs are not promptly addressed or vocalized by the team, the simulation will proceed as indicated. If the team is appropriately and promptly resuscitating the patient, allow the team to proceed without interruption until branch point 8:00A or 8:00B.	If no O2 is given, SaO2 rapidly declines. If hypoxia is not recognized by the team, the RN will prompt the team to address. If no IV fluids are given, the patient’s blood pressure will rapidly decline. The RN will prompt the team for an updated set of vitals. The BP will be 68/palpable. If no blood glucose measurement is acquired, the RN will prompt the team to obtain a full set of vitals. If the team still does not obtain POC glucose, the RN will ask the team, “Why do you think the patient is so altered?” If the team still does not obtain POC glucose, the RN voices a concern. If naloxone IV is given empirically (regardless of the dose), there will be no change in the patient’s mental status. If the patient’s temperature is not measured, the RN will comment on the patient’s cold skin. If the patient’s temperature is not addressed (i.e., no warming interventions are initiated), the RN will prompt the team to intervene.	HR: 45 BP: 85/50 RR: 10 SaO2: Declines to 75% HR: 45 BP: 68/palpable RR: 10 SaO2: 90% on O2 POC blood glucose: 55 Repeat blood glucose after D50 given: 80
8:00 A	The patient’s condition continues to deteriorate despite appropriate resuscitation efforts. If the patient codes, cardiopulmonary resuscitation (CPR) should be initiated immediately, following ACLS protocol. Patient is intubated. Post-intubation CXR and ABG should be acquired and interpreted. Respiratory therapy (RT) paged. Initiate norepinephrine infusion.	Patient becomes unresponsive with impending respiratory failure despite via non-rebreather mask (NRB). Bag valve mask (BVM) ventilation should be initiated. BP is unresponsive to fluids and vasopressors. Unstable bradycardia is refractory to atropine or transcutaneous pacing. If the team attempts rapid sequence intubation (RSI) without pre-intubation resuscitation with vasopressors, the patient suffers pulseless electrical activity (PEA) cardiac arrest. BP will be unattainable. RN will prompt the team to check pulses. Return of spontaneous circulation (ROSC) obtained after 1 round of CPR and ×1 administration of 1 mg (1:10000) epinephrine IV.	T: 34.9° C HR: 30 BP: 70/50 RR: 8 SaO2: 84% on O2 If push-dose epinephrine is used: HR 65 BP 90/60 RR (no change) SaO2 (slight increase by 2- 5%) If norepin-ephrine infusion is started, HR and BP will gradually increase up to the above parameters over a duration of 90 seconds.
8:00 B	Clinical deterioration with a delay in intubation. If the patient codes, CPR should be initiated immediately, following ACLS protocol. Patient is intubated after achieving ROSC. Post-intubation CXR and ABG should be acquired and interpreted. Respiratory therapy (RT) paged. Address persistent hypotension.	The team does not vocalize a need to intubate or prepare for intubation. The patient becomes apneic. SpO2 sharply declines despite oxygenation via NRB. Patient becomes progressively and rapidly more bradycardic. RN prompts learners and brings their attention to the monitor. If the team does not begin ventilating the patient via BVM promptly, the patient suffers PEA cardiac arrest. If this is unrecognized by the team, the RN should prompt a pulse check. CPR initiated. ROSC obtained after 1 round of CPR and ×1 administration of 1 mg (1:10000) epinephrine IV. The patient should be intubated during code or immediately after. Recycle vital signs. The team should order a post-intubation chest x-ray and repeat ABG. The team should recognize and address hypotension with initiation of vasopressors, eg, norepinephrine infusion. If left unaddressed, RN will prompt team of the refractory hypotension despite IV fluid resuscitation.	T: 34.9° C HR: 20 BP: 70/50 RR: 0 SaO2: 62% on O2 Post-ROSC vitals: T: 34.9° C HR: 35 BP: 70/50 RR: 14 SaO2: 99% on 100% FiO2 (fraction of inspired oxygen) Vitals after initiation of vasopressors: T: 34.9° C HR: 65 BP: 90/60 RR: 14 SaO2: 99% on 100% FiO2
10:00	Labs and imaging return. Confirmation of hypothyroidism based on TSH results.	Laboratory studies return and are presented to the team. Learner is expected to administer treatments for decompensated hypothyroidism including: Levothyroxine 200-400 mcg IV bolus Hydrocortisone 100mg IV If treatments for hypothyroidism are not given, the patient remains hypotensive and requires titrating to incrementally higher doses of vasopressors. Consult endocrinology who may provide advice on appropriate therapies	Post-intubation vitals: T: 34.9° C HR: 50 BP: 90/60 RR: (set to learner’s discretion on ventilator) O2: 99% on 100% FiO2
15:00 (Case Completion)	Treatment for hypothyroidism given. Reevaluate the patient. Update family. Learner presents the case to the intensivist. Disposition to the ICU	The patient will stabilize. Page is returned by critical care. Patient is accepted for admission after learner presents the case	T: 34.9° C HR: 60 BP: 110/60 RR: (set to learner’s discretion on ventilator) SaO2: 99% on 100% FiO2

**Table t21-jetem-10-1-s1:** 

**Critical Actions:** Initiate IV fluid resuscitation Recognize and treat hypoglycemia Intubate for acute hypoxic respiratory failure and deteriorating LOC Recognize myxedema coma Initiate treatment with IV levothyroxine and a glucocorticoid Consult endocrinology Disposition to the ICU	0:00

**Table t22-jetem-10-1-s1:** Milestones assessment:

	Milestone	Did not achieve level 1	Level 1	Level 2	Level 3
1	**Emergency Stabilization (PC1)**	□ Did not achieve Level 1	□ Recognizes abnormal vital signs	□ Recognizes an unstable patient, requiring intervention Performs primary assessment Discerns data to formulate a diagnostic impression/plan	□ Manages and prioritizes critical actions in a critically ill patient Reassesses after implementing a stabilizing intervention
2	**Performance of focused history and physical (PC2)**	□ Did not achieve Level 1	□ Performs a reliable, comprehensive history and physical exam	□ Performs and communicates a focused history and physical exam based on chief complaint and urgent issues	□ Prioritizes essential components of history and physical exam given dynamic circumstances
3	**Diagnostic studies (PC3)**	□ Did not achieve Level 1	□ Determines the necessity of diagnostic studies	□ Orders appropriate diagnostic studies. Performs appropriate bedside diagnostic studies/procedures	□ Prioritizes essential testing Interprets results of diagnostic studies Reviews risks, benefits, contraindications, and alternatives to a diagnostic study or procedure
4	**Diagnosis (PC4)**	□ Did not achieve Level 1	□ Considers a list of potential diagnoses	□ Considers an appropriate list of potential diagnosis May or may not make correct diagnosis	□ Makes the appropriate diagnosis Considers other potential diagnoses, avoiding premature closure
5	**Pharmacotherapy (PC5)**	□ Did not achieve Level 1	□ Asks patient for drug allergies	□ Selects an medication for therapeutic intervention, consider potential adverse effects	□ Selects the most appropriate medication and understands mechanism of action, effect, and potential side effects Considers and recognizes drug-drug interactions
6	**Observation and reassessment (PC6)**	□ Did not achieve Level 1	□ Reevaluates patient at least one time during case	□ Reevaluates patient after most therapeutic interventions	□ Consistently evaluates the effectiveness of therapies at appropriate intervals
7	**Disposition (PC7)**	□ Did not achieve Level 1	□ Appropriately selects whether to admit or discharge the patient	□ Appropriately selects whether to admit or discharge Involves the expertise of some of the appropriate specialists	□ Educates the patient appropriately about their disposition Assigns patient to an appropriate level of care (ICU/Tele/Floor) Involves expertise of all appropriate specialists
9	**General Approach to Procedures (PC9)**	□ Did not achieve Level 1	□ Identifies pertinent anatomy and physiology for a procedure Uses appropriate Universal Precautions	□ Obtains informed consent Knows indications, contraindications, anatomic landmarks, equipment, anesthetic and procedural technique, and potential complications for common ED procedures	□ Determines a back-up strategy if initial attempts are unsuccessful Correctly interprets results of diagnostic procedure
20	**Professional Values (PROF1)**	□ Did not achieve Level 1	□ Demonstrates caring, honest behavior	□ Exhibits compassion, respect, sensitivity and responsiveness	□ Develops alternative care plans when patients’ personal beliefs and decisions preclude standard care
22	**Patient centered communication (ICS1)**	□ Did not achieve level 1	□ Establishes rapport and demonstrates empathy to patient (and family) Listens effectively	□ Elicits patient’s reason for seeking health care	□ Manages patient expectations in a manner that minimizes potential for stress, conflict, and misunderstanding. Effectively communicates with vulnerable populations, (at risk patients and families)
23	**Team management (ICS2)**	□ Did not achieve level 1	□ Recognizes other members of the patient care team during case (nurse, techs)	□ Communicates pertinent information to other healthcare colleagues	□ Communicates a clear, succinct, and appropriate handoff with specialists and other colleagues Communicates effectively with ancillary staff

**Table 1:** Level of training of participants

Level of Training	Number	% of total
Medical student/PGY-1 resident	16	48
PGY-2 resident	6	18
PGY-3 resident	10	30
PGY-4 resident	1	3

## INSTRUCTOR MATERIALS

**Case Title:** A Cold Case: Myxedema Coma

**Case Description & Diagnosis (short synopsis):** A 68-year-old female with a history of hypothyroidism presents to the ED via EMS with altered mental status secondary to myxedema coma precipitated by recent surgery. The patient is hypothermic, bradycardic, and hypotensive on arrival. The patient can provide a limited history; however, her level of consciousness (LOC) and hemodynamic status continue to deteriorate despite intravenous (IV) fluids, necessitating intubation. The unstable bradycardia is resistant to both pharmacological and electrical intervention. Laboratory studies confirm the diagnosis of decompensated hypothyroidism. Further management will involve administration of levothyroxine IV. The team is expected to consult endocrinology and critical care. Ultimate disposition is the ICU.

**Equipment or Props Needed:**

- Computerized adult mannequin
- Cardiac monitor
- IV poles
- Defibrillator with transcutaneous pacing pads
- Airway cart and intubation equipment
- Warming blankets
- IV fluid bags
- Medications including: 50% dextrose in water, 1:10000 epinephrine, 10 mL syringes of normal saline, norepinephrine, atropine, levothyroxine, and hydrocortisone

[Fig f7-jetem-10-1-s1] [Fig f8-jetem-10-1-s1] [Fig f9-jetem-10-1-s1]

**Actors needed:**

- RN
- Daughter of patient
- EMS provider

The RN may assist with acquisition of vital signs in the ED and attach monitor leads and continuous pulse oximetry. The RN will establish IV access if ordered. The RN is responsible for responding to requests and orders from the team leader with closed loop communication including attaching monitoring equipment, drawing labs, and administering requested medications.

The EMS provider is in the room upon arrival of the learner. EMS will provide a brief handover report and vital signs acquired in the field, if requested by the learner.

The daughter is in the room and may provide a detailed background history including medications, if requested.

**Stimulus Inventory:** [Table t3-jetem-10-1-s1]

**Background and brief information:** The patient arrives via EMS to a well-resourced emergency department in winter. Daughter is present and may provide additional history.

**Initial presentation:** Patient is a 68-year-old female brought to the ED via EMS for altered mental status. The patient was found by her daughter and was confused and cold. Her daughter states she has been depressed for the past few weeks, and it’s unclear if she has been taking her medications. She is concerned that her mother has not been taking care of herself. The heat was off and it was quite cold in the home on EMS arrival to scene.

- Past medical history: hypothyroidism, hypertension, hypercholesterolemia, chronic constipation, and major depression.
- Past surgical history: appendectomy
- Allergies: none
- Medications: levothyroxine, simvastatin, sertraline, metoprolol
- Social history: lives alone, daughter lives nearby, social drinker, never smoker
- Family history: noncontributory
- Vital signs:
  - Heart rate (HR) - 52
  - Respiratory rate (RR) - 12, laboured
  - Blood Pressure (BP) - 95/68
  - Temperature - 34.1°C (core)
  - Oxygen saturation - 85% on room air
  - Weight - 85kg

Assessment: Lying supine on the gurney. The patient is confused and lethargic. Oriented to person, but not to time or place.

**How the scene unfolds:** The patient presents lethargic and confused. The learner should recognize and intervene on the abnormal vital signs early in the case. The patient remains hypotensive and bradycardic despite fluid resuscitation and appropriate interventions. The patient will require intubation for impending respiratory failure. The patient will remain hemodynamically unstable despite IV fluids and vasopressor support until levothyroxine is administered, after which the patient’s vital signs will improve. The case will end once the learner has stabilized the patient and arranged for disposition to the ICU.

**Critical actions:**

- Initiate IV fluid resuscitation
- Recognize and treat hypoglycemia
- Intubate for acute hypoxic respiratory failure and deteriorating LOC
- Recognize myxedema coma
- Initiate treatment with levothyroxine
- Consult endocrinology
- Disposition to the ICU

**Case Title:** A Cold Case: Myxedema Coma

**Chief Complaint:** Patient is a 68-year-old female brought to the ED via EMS for altered mental status.[Table t4-jetem-10-1-s1]

**General Appearance:** Obese, unwell, and lethargic

**Primary Survey:**

- **Airway:** Patent
- **Breathing:** Bradypnea, shallow and labored respirations
- **Circulation:** Bradycardic, pulses thready and equal bilaterally, capillary refill time 4 seconds, cool/dry extremities

**History:**

- **History of present illness:** Per EMS, the patient’s daughter found the patient at her home, confused and lethargic, and called 911. The interior of the house was quite cold, and she noticed that the heating system was turned off. The case takes place in Michigan in the month of January. For the past few weeks, the patient has seemed depressed and has not been out shopping, which is atypical for her according to her family. The daughter is unsure if she has been taking her medications. She does have a history of hypothyroidism, hypertension, hypercholesterolemia, and major depression.
- **Past medical history:** hypothyroidism, hypertension, hypercholesterolemia, and major depression.
- **Past surgical history:** appendectomy
- **Patient’s medications:** levothyroxine, simvastatin, sertraline, metoprolol
- **Allergies:** no known drug allergies (NKDA)
- **Social history:** lives alone, adult daughter lives nearby, retired shopkeeper. Drinks wine occasionally. Never smoked.
- **Family history:** noncontributory

**Secondary Survey/Physical Examination:**

- **General appearance:** Obese, unwell and lethargic
- **HEENT:**
  ○ **Head:** Thinning and coarse hair
  ○ **Eyes:** Bilateral periorbital edema
  ○ **Ears:** Normal
  ○ **Nose:** Normal
  ○ **Mouth/Throat:** Macroglossia
- **Neck:** Normal
- **Heart:** Bradycardic, S1 and S2 heart sounds audible, no murmurs
- **Lungs:** Bradypnea, shallow respirations, air entry equal bilaterally with no added sounds
- **Abdominal/GI:** Protuberant, soft and non-tender to palpation, reduced bowel sounds
- **Genitourinary:** Normal
- **Rectal:** Normal
- **Extremities:** Cool peripheries
- **Back:** Normal
- **Neuro:** Eyes closed, responds to verbal stimuli, confused and oriented to person only, anxious, increasingly obtunded
- **Skin:** Cold and dry, bilateral, non-pitting, pretibial myxedema
- **Lymph:** Normal
- **Psych:** Depressed

*ECG: Sinus Bradycardia*

Ewingdo, CC. ECG Sinus Bradycardia. In: Wikimedia Commons.

https://commons.wikimedia.org/wiki/File:ECG_Sinus_Bradycardia_43_bpm.jpg. Published November 14, 2020. Accessed August 10, 2022. BY-SA 4.0[Fig f10-jetem-10-1-s1]

*Previous ECG*

Burns E, Buttner R. Normal Sinus Rhythm. In: Life in the Fast Lane. https://litfl.com/normal-sinus-rhythm-ecg-library/Published Mar 11, 2021. Accessed August 10, 2022. CC BY-NC-SA 4.0 DEED[Fig f11-jetem-10-1-s1]

*Suggested Moulage*

Cabot Richard C. ed, Salinger, Julius L, tr. Image: Aged Fifty-two; Advanced Myxedema *Diseases of Metabolism and of the Blood, Animal Parasites, Toxicology*. 1909; p. 202. New York and London: D. Appleton and Company.

https://www.flickr.com/photos/internetarchivebookimages/20532661680/ [Fig f12-jetem-10-1-s1]

Fred HL, van Dijk HA. Pretibial myxedema and thyroid acropachy accompanying hyperthyroidism. This 33-year-old woman presented with painless swelling of her fingers and lower legs of about four months’ duration.

https://commons.wikimedia.org/wiki/File:Myxedema.jpg

This work is licensed under a Creative Commons Attribution License (CC-BY 2.0).[Fig f13-jetem-10-1-s1]

*Pre-intubation CXR*

Bickle, I. Normal chest radiograph - female. In: Radiopaedia.org.

https://radiopaedia.org/cases/33225. Published January 13, 2015. Accessed August 10, 2022. rID: 33225. CC BY-NC-SA 3.0 DEED[Fig f14-jetem-10-1-s1]

*CT head without contrast*

CT Head: Author’s own image (Dr. Andrew Bazakis)[Fig f15-jetem-10-1-s1][Fig f16-jetem-10-1-s1][Fig f17-jetem-10-1-s1][Fig f18-jetem-10-1-s1]

*Echocardiographies (video)*

Bowra J, Kaynama, K. In: The POCUS Atlas. Severe Left Ventricular Failure.

- https://www.thepocusatlas.com/left-ventricular-dysfunction/x6hpkvwop5rxfzlr7mdayugpkkzlwy. Accessed November 11, 2024. CC BYNC 4.0.
- https://images.squarespacecdn.com/content/v1/58118909e3df282037abfad7/1515766726272-5OZFEY1E473WFDB1PKGM/ezgif.com-optimize+%2820%29.gif?format=750w

Laboratory Results:[Table t5-jetem-10-1-s1][Table t6-jetem-10-1-s1][Table t7-jetem-10-1-s1][Table t8-jetem-10-1-s1][Table t9-jetem-10-1-s1][Table t10-jetem-10-1-s1][Table t11-jetem-10-1-s1][Table t12-jetem-10-1-s1][Table t13-jetem-10-1-s1][Table t14-jetem-10-1-s1][Table t15-jetem-10-1-s1][Table t16-jetem-10-1-s1][Table t17-jetem-10-1-s1][Table t18-jetem-10-1-s1][Table t19-jetem-10-1-s1]

*ABG, post-intubation*

pH-7.21
paCO2-50 mmHg
paO2-100 mmHg
HCO3-20 mmol/L
SaO2-100%

*Post-intubation CXR*

Shah S, Pitroda P, Patel K, Chandak R, Ford T. Polymorphic ventricular tachycardia secondary to subarachnoid haemorrhage: A rare occurrence in the setting of normal QTc. In: *Cardiology Research*. 8. 232–235. 10.14740/cr574w

Published October 2017. Accessed August 10, 2022. CC BY-NC 4.0.

https://www.researchgate.net/publication/320679189_Polymorphic_Ventricular_Tachycardia_Secondary_to_Subarachnoid_Haemorrhage_A_Rare_Occurrence_in_the_Setting_of_Normal_QTc.[Fig f19-jetem-10-1-s1]

[Table t20-jetem-10-1-s1]

## OPERATOR MATERIALS

**Diagnosis:**

Myxedema Coma

**Disposition:**

Admission to the intensive care unit (ICU)

## DEBRIEFING AND EVALUATION PEARLS

### Myxedema Coma

**Case Summary:**

- Myxedema coma with acute respiratory failure and hemodynamic instability

**Learning objectives:**

- Develop a broad differential diagnosis for a patient with altered mental status and direct an appropriate workup [Medical Knowledge].
- Rapidly assess and initiate resuscitation of a critically ill patient including airway management, ventilatory support, volume repletion and vasopressor support [Medical Knowledge].
- Appraise physical examination and history findings to reach diagnosis of myxedema coma and to assemble treatment plan for the definitive treatment [Medical Knowledge].
- Demonstrate effective leadership of a treatment team in the emergency department setting; use of effective closed-loop communication [Interpersonal and Communication Skills, Professionalism].
- Direct proper disposition to the ICU and appropriate consultation with endocrinology [Systems-Based Practice].

**Learning points:**

1. Recognize the hemodynamically unstable patient and perform a rapid primary assessment including point of care blood glucose and a rectal temperature.
2. Address and intervene on vital sign abnormalities.
3. Demonstrate the clinical suspicion for myxedema coma amidst a broad differential diagnosis for a patient with altered mental status.
4. Obtain key elements of the history in a patient with altered mental status from collateral sources such as the paramedics and family members.
5. Note physical examination features in myxedema coma include: hypothermia, bradycardia, periorbital swelling, macroglossia, dry skin, coarse and brittle hair, alopecia, non-pitting edema.

**Pearls:**

Myxedema coma is often associated with an underlying precipitating factor, such as: infection eg, pneumonia (most common), environmental exposures, infarction/ischemia, drugs, medication non-adherence, toxicological exposures, trauma, electrolyte abnormalities.

Clinical features associated with myxedema coma (based on system):

- HEENT: hoarseness, periorbital swelling, broad nose, macroglossia
- CNS: lethargy, confusion
- Skin: Dry skin, coarse and brittle hair, alopecia, non-pitting edema
- CVS: Bradycardia
- Respiratory: Bradypnea, respiratory failure, pleural effusion
- GI: Constipation
- Reproductive: amenorrhea, menorrhagia
- Hematology: coagulopathy
- Metabolic: hyponatremia, hypoglycemia

Differential diagnoses to consider with bradycardia, hypotension, and altered mental status:

- Infection/sepsis
- Drug overdose
  — Beta blocker
  — Calcium channel blocker
  — Digoxin
- Toxicological ingestion
- Ethanol toxicity
- Opioid overdose
- Environmental exposure
- Intracranial pathology/elevated intracranial pressure
- Neurogenic shock
- Primary bradyarrhythmia
- Adrenal crisis

Principles of resuscitation

- Airway:
  - Anticipate a difficult airway; patients with myxedema coma can have macroglossia and pharyngeal edema.^1^ Emphasis is placed on preparing for a difficult airway by having adjuncts at the bedside, including suction, bougie, video-assisted laryngoscopy, and a surgical airway kit.
- Breathing:
  - Preoxygenation technique includes bag-valve-mask ventilation with nasal cannula
- Circulation:
  - In the hypotensive patient with impending respiratory failure in need of a definitive airway, consider use of vasopressors prior to intubation including push-dose epinephrine or norepinephrine infusion
  - Learners should be educated on the method of formulating push-dose epinephrine at the bedside using: i) 1:10000 epinephrine and ii) a 10 mL syringe of normal saline. Method:^2^
    - Draw 1 mL of 1:10000 which equates to 0.1 mg (100 mcg) of epinephrine
    - Discard 1 mL of a 10 mL syringe of normal saline
    - Insert 1 mL of 1:10000 epinephrine into the syringe containing 9 mL of normal saline
      - The result is 10 mL containing 100 mcg of epinephrine, i.e., 10 mcg per mL

ECG: ECG findings in hypothyroidism are non-specific and commonly reveal sinus bradycardia, low-voltage QRS complexes and widespread T-wave inversions. These findings are due to i) reduced sympathetic drive, and ii) direct effect of low thyroxine on the myocardium, namely reduced inotropy and chronotropy.^3^

Laboratory findings:

- Common electrolyte abnormalities in myxedema coma:
  - hyponatremia - due to poor free water clearance
  - hypoglycemia - due to downregulation
- The diagnosis of myxedema coma is confirmed by thyroid function studies. However, often in the emergent setting, laboratory results may not return in a timely fashion and thus the diagnosis of myxedema coma should be made clinically in the ED.^4^ Nevertheless, it is important to order these studies to guide appropriate patient care.
- Interpretation of thyroid function tests:
  - Primary hypothyroidism (inadequate production of thyroid hormone from the thyroid gland): high serum thyroid stimulating hormone (TSH), low free T4 (fT4)
  - Secondary hypothyroidism (pituitary dysfunction): low serum TSH, low fT4

Diagnosis of myxedema coma can also be supported by using a scoring system developed by Chiong *et al* which utilizes criteria including Glasgow coma scale (GCS), TSH, temperature, heart rate, and precipitating event.^5^ Learners are encouraged to consider using this tool if there is suspicion for myxedema coma to help support their decision making.

Empiric treatment of decompensated hypothyroidism:

- Thyroid hormone replacement
  - T4 (levothyroxine) 200-400 mcg IV (loading dose) 6
    - T4 is the standard of care for the treatment of hypothyroidism and should be given rapidly. Patient factors that should prompt the clinician to consider smaller doses of T4 include: small stature, elder, or history of coronary artery disease or cardiac dysrhythmia.^7^
    - Subsequent doses of levothyroxine are 1.6 mcg/kg body weight per day p.o. (or 75% if given IV).
  - T4 and T3 (liothyronine) combination therapy. Combination therapy has not shown substantial evidence to be efficacious over T4 monotherapy and is not recommended.^8^
- Steroids
  - Hydrocortisone 100 mg IV q8h
    - Given as a stress-dose steroid due to the high likelihood of accompanying adrenal insufficiency. Hydrocortisone is the preferred agent due to the combined glucocorticoid and mineralocorticoid properties.^6^

**Other debriefing points:**

Highlight qualities of a high-functioning team in a resuscitation:

- Assignment and clear definition of roles
- Closed loop communication amongst the team and the RN
- Fostering a supportive team environment
- Effective team leadership

Key physical features of the high-fidelity moulage that suggest hypothyroidism:

- macroglossia
- a wig with thinning hair/alopecia
- bilateral periorbital edema
- scaling skin
- bilateral pretibial myxedema

Discussion with consultants

- If the learners call the appropriate consultant i.e., endocrinology, they may be provided with treatment recommendations including dosing for levothyroxine and hydrocortisone.

## SIMULATION ASSESSMENT

*Myxedema Coma*

Learner: _________________________________________

***Assessment Timeline***

This timeline is to help observers assess their learners. It allows observer to make notes on when learners performed various tasks, which can help guide debriefing discussion.[Table t21-jetem-10-1-s1]

**Critical Actions:**

□ Initiate IV fluid resuscitation
□ Recognize and treat hypoglycemia
□ Intubate for acute hypoxic respiratory failure and deteriorating LOC
□ Recognize myxedema coma
□ Initiate treatment with IV levothyroxine and a glucocorticoid
□ Consult endocrinology
□ Disposition to the ICU

**Summative and formative comments:**

**Milestones assessment:** [Table t22-jetem-10-1-s1]
