# Supplementary material for: A Cold Case: Myxedema Coma
Source: J Educ Teach Emerg Med. 2025 Jan 31;10(1):S1–S42. doi: 10.21980/J8VM0J (PMC11801491; doi:10.21980/J8VM0J)
Supplement: Supplementary file 1 [file 10-1-S1-supp1.pptx]

## Slide 1
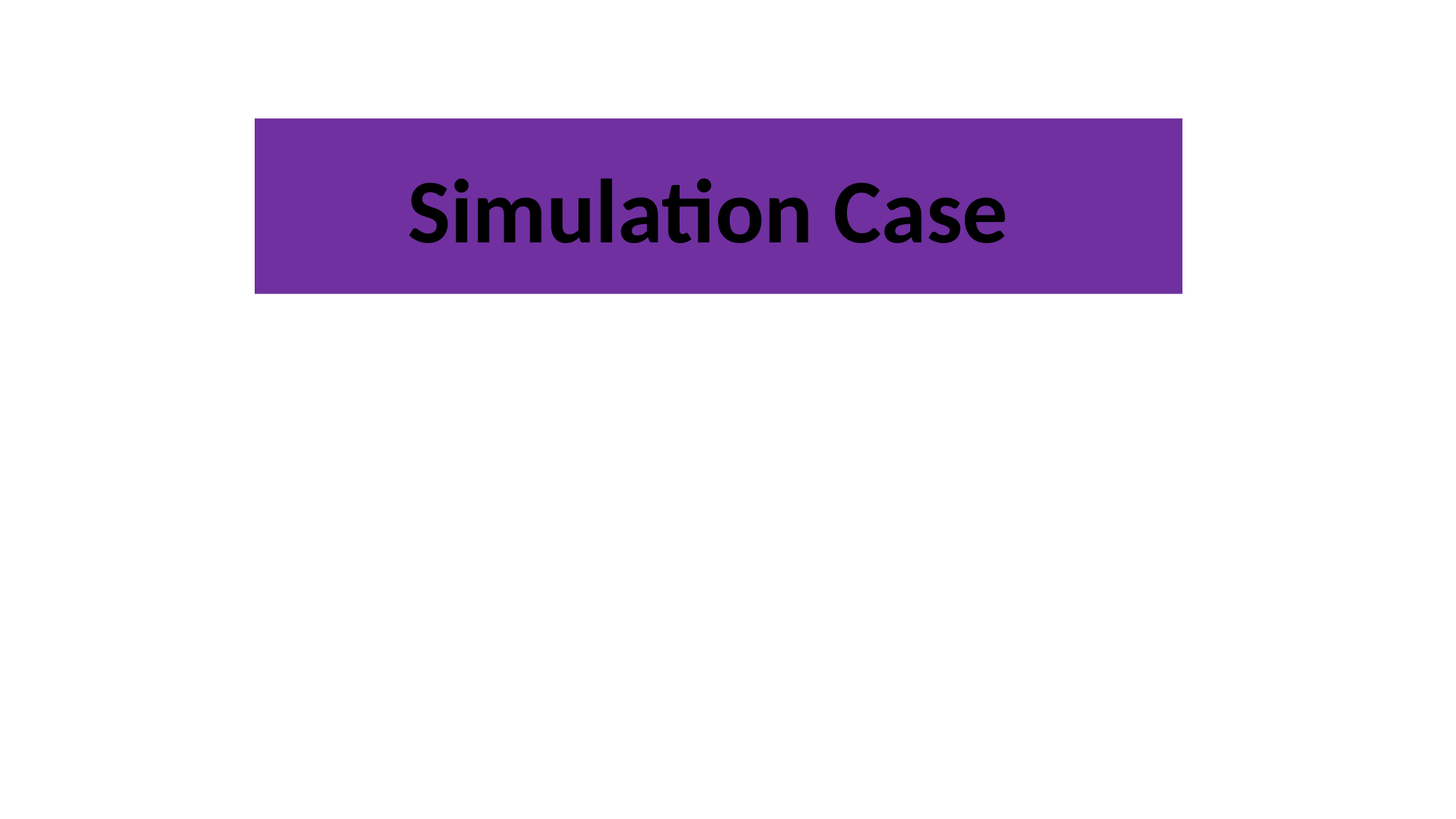

# Simulation Case

## Slide 2
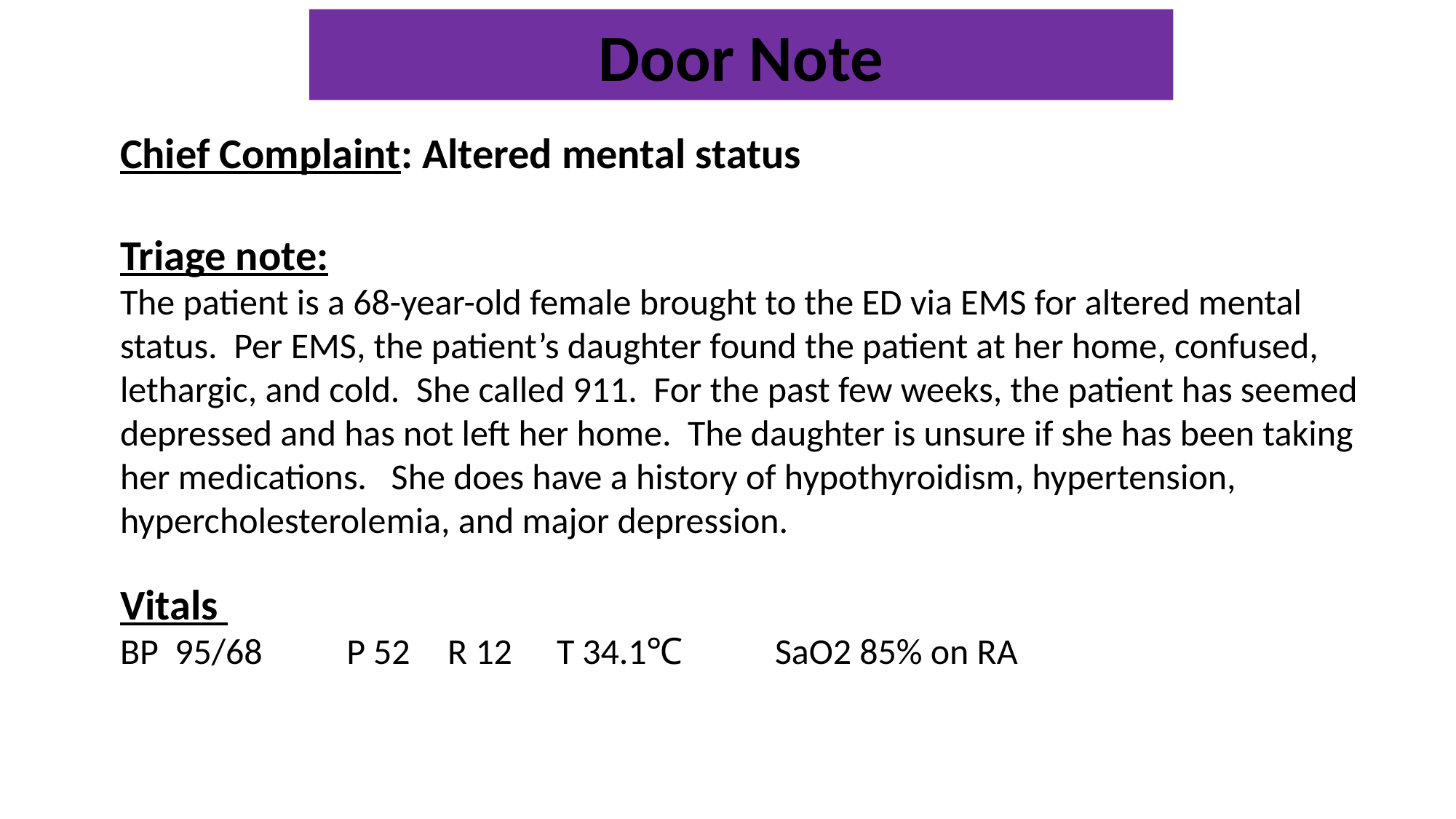

# Door Note
Chief Complaint: Altered mental status
Triage note:
The patient is a 68-year-old female brought to the ED via EMS for altered mental status. Per EMS, the patient’s daughter found the patient at her home, confused, lethargic, and cold. She called 911. For the past few weeks, the patient has seemed depressed and has not left her home. The daughter is unsure if she has been taking her medications. She does have a history of hypothyroidism, hypertension, hypercholesterolemia, and major depression.
Vitals
BP 95/68	 P 52	R 12	T 34.1℃	SaO2 85% on RA

## Slide 3
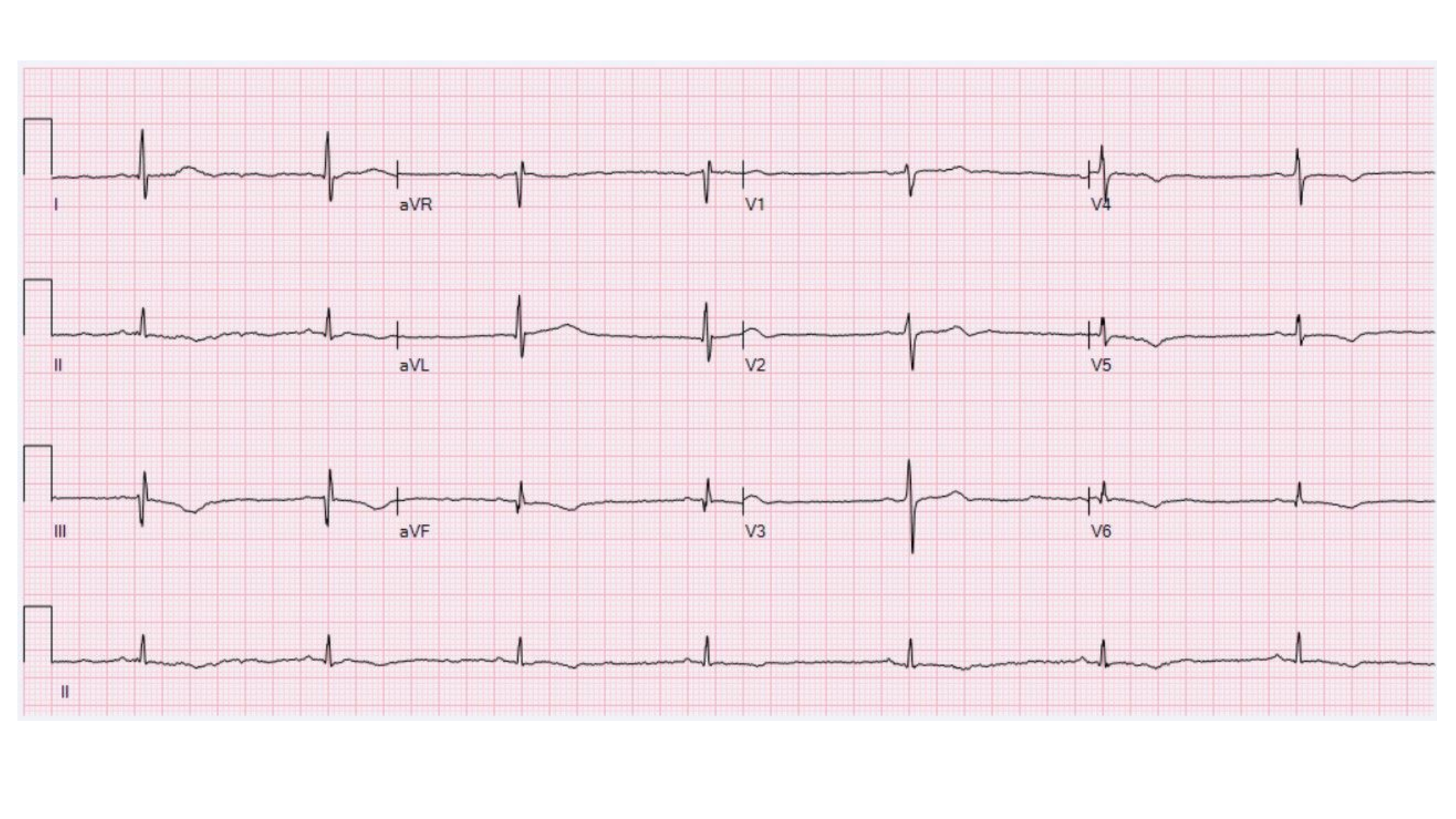

## Slide 4
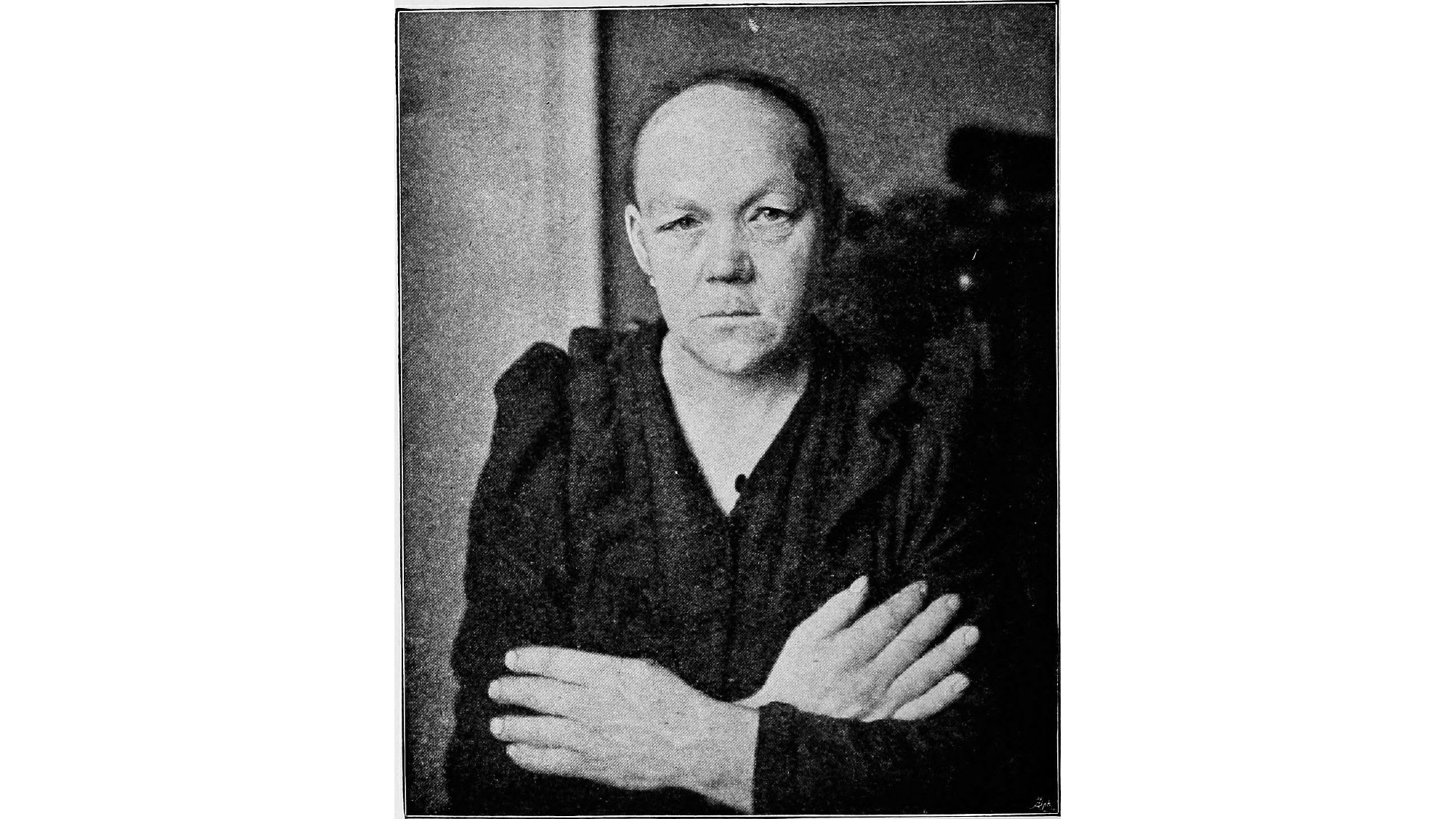

## Slide 5
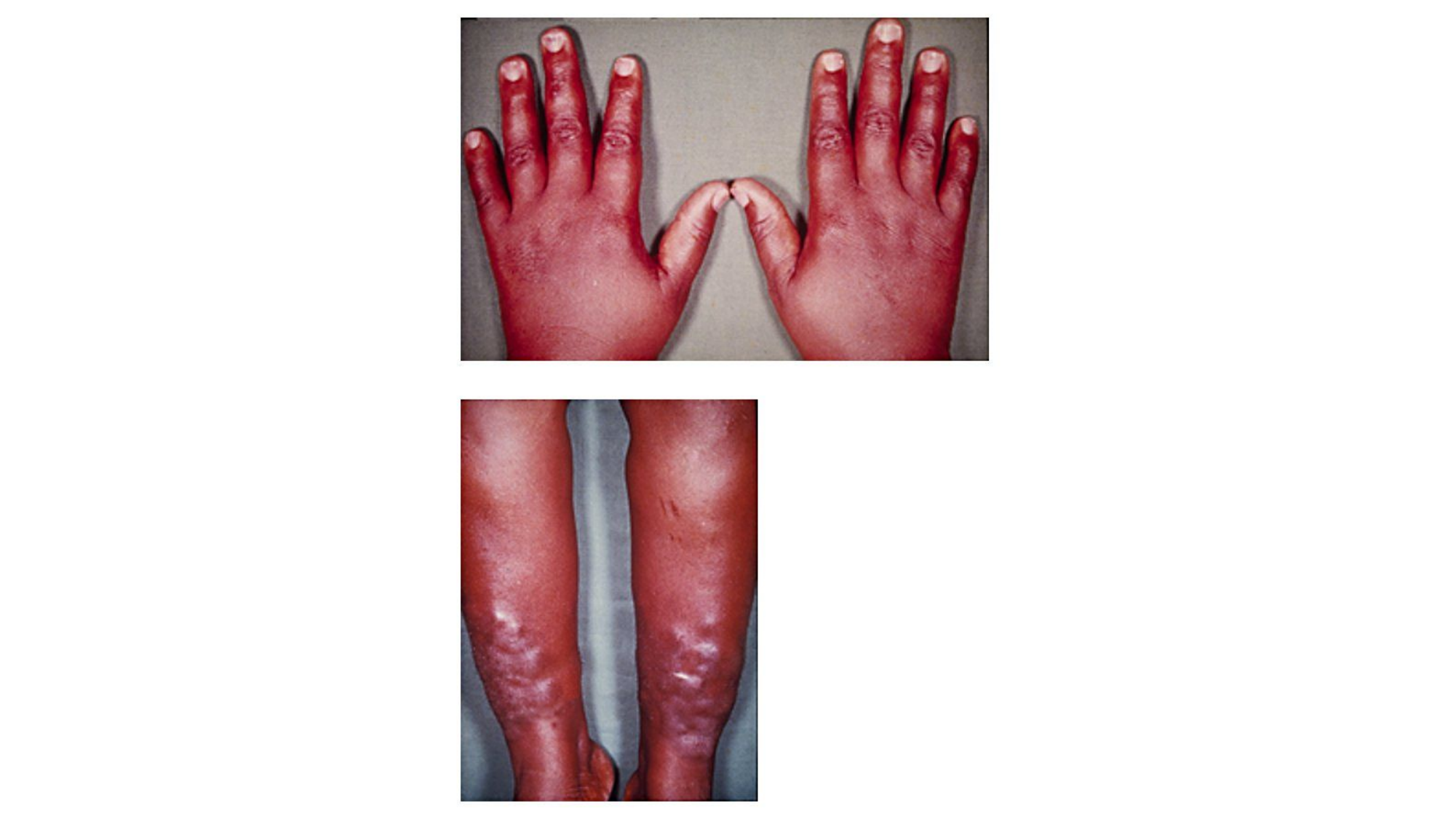

## Slide 6
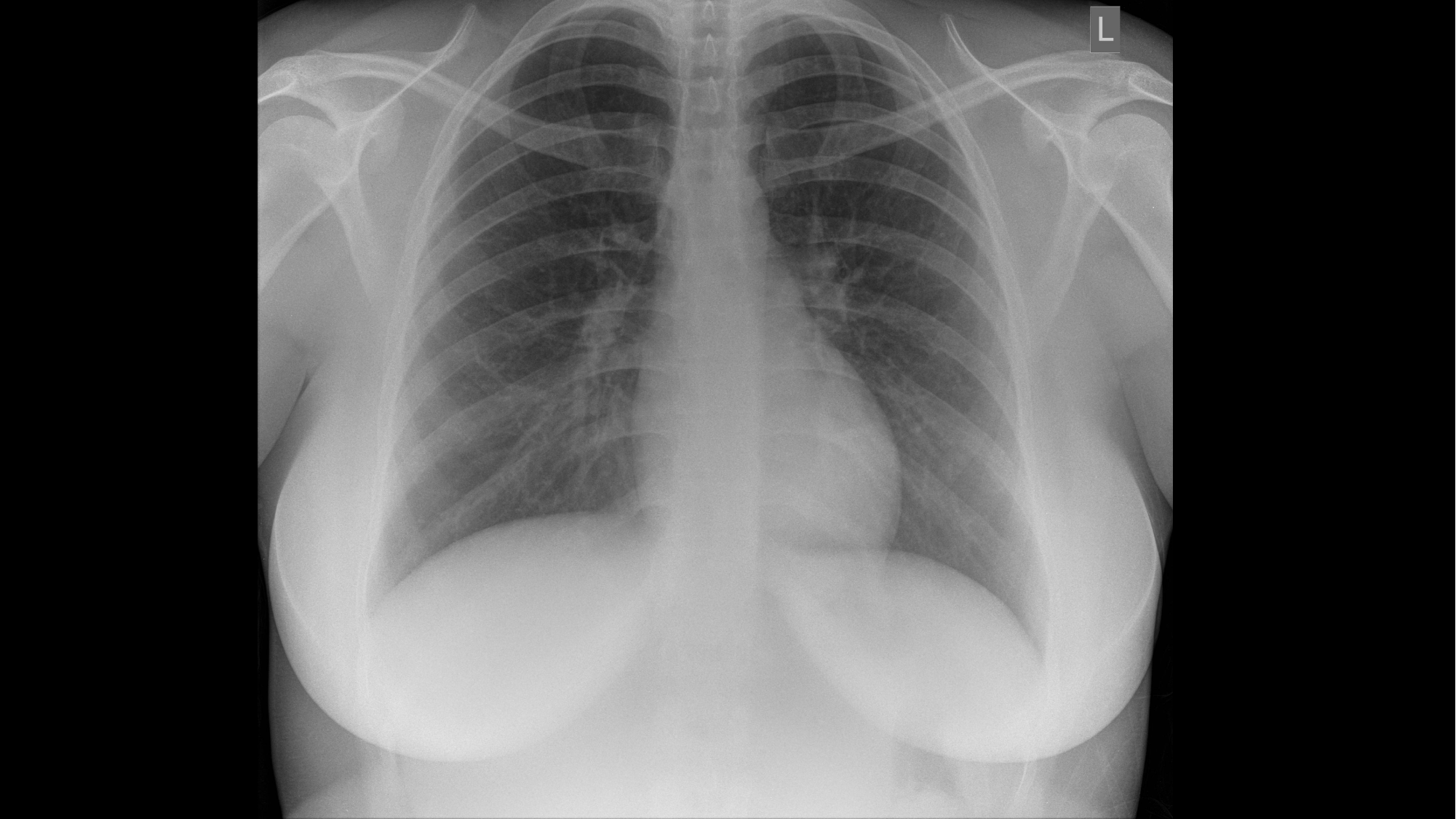

## Slide 7
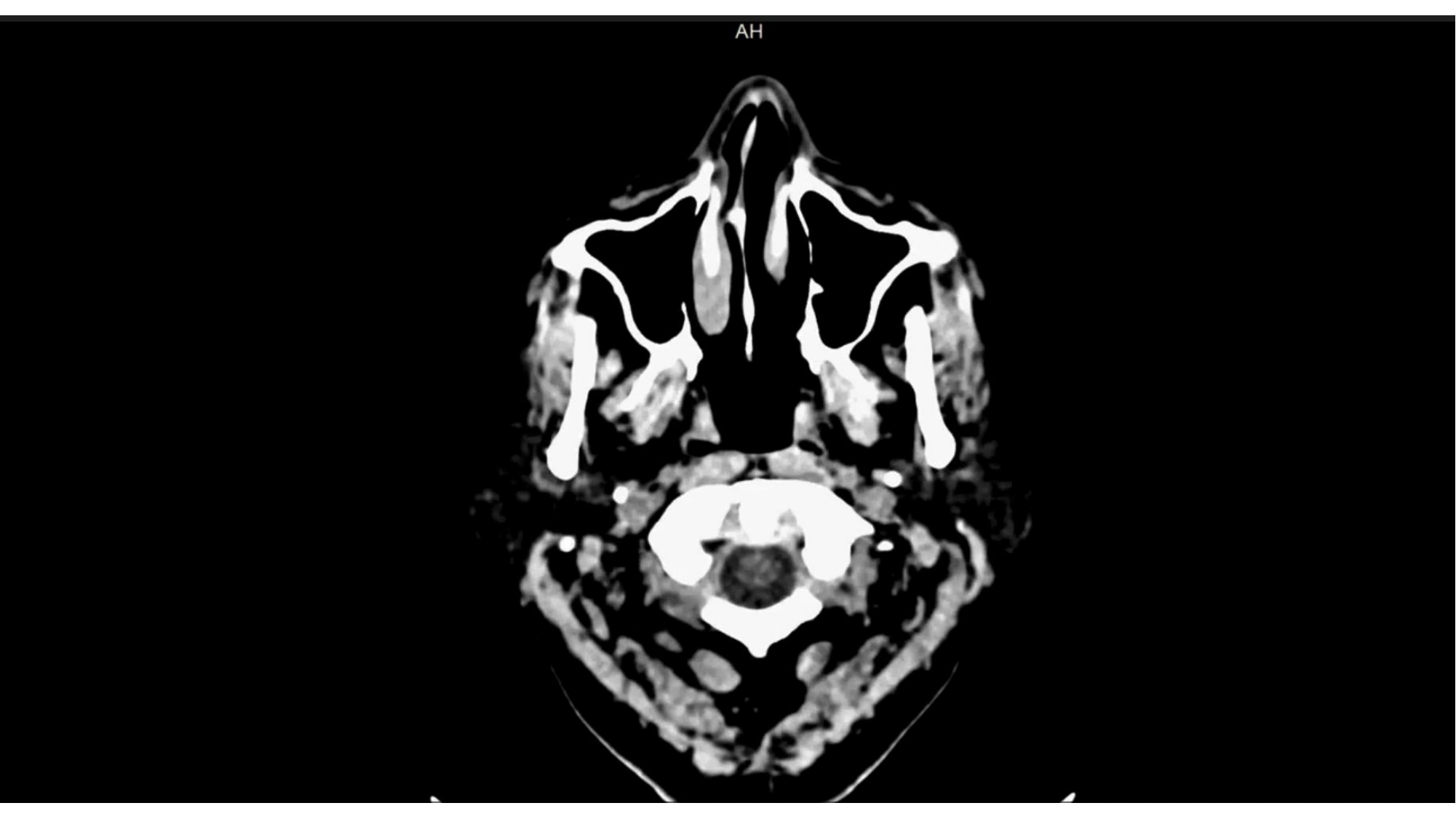

## Slide 8
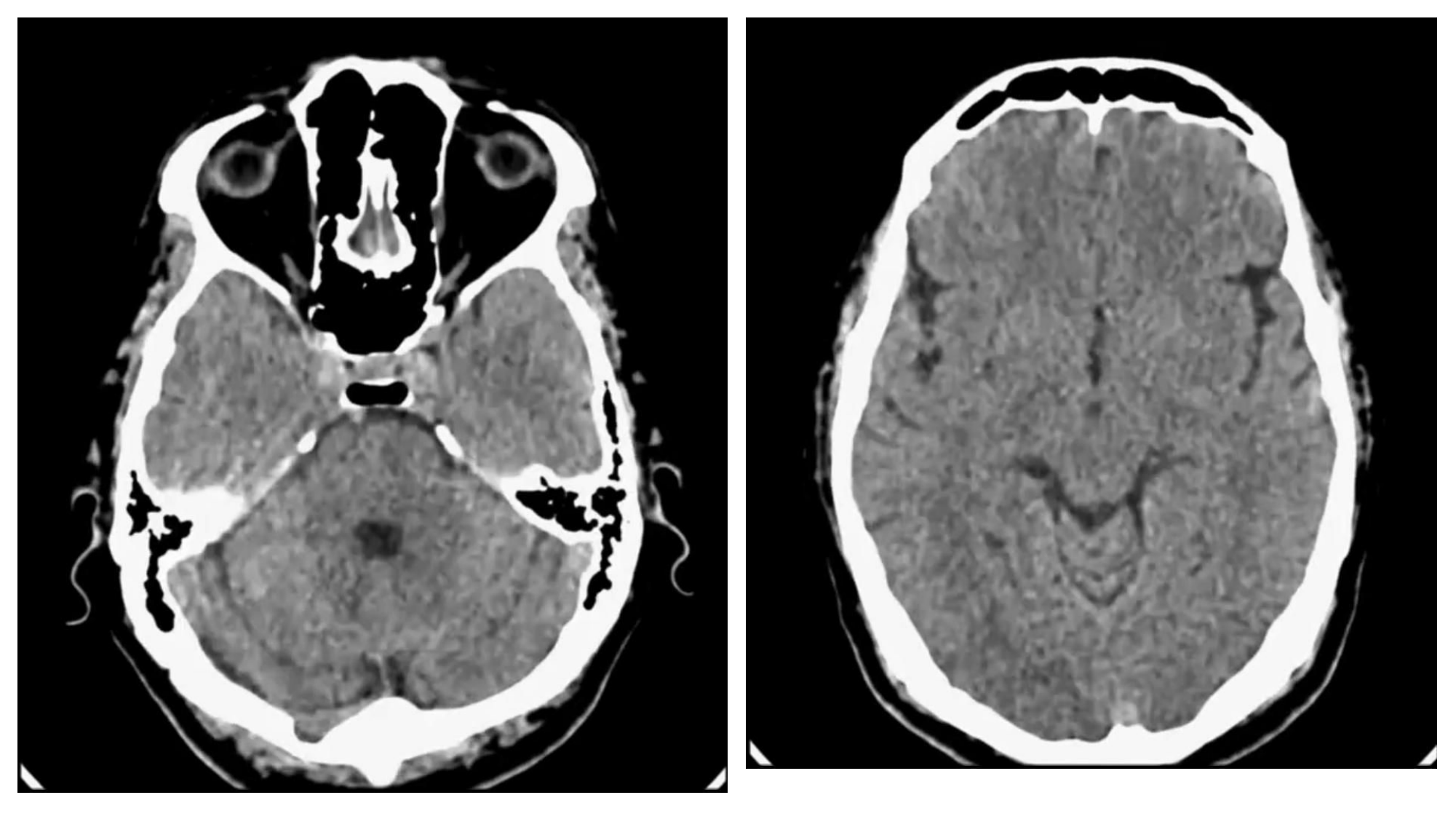

## Slide 9
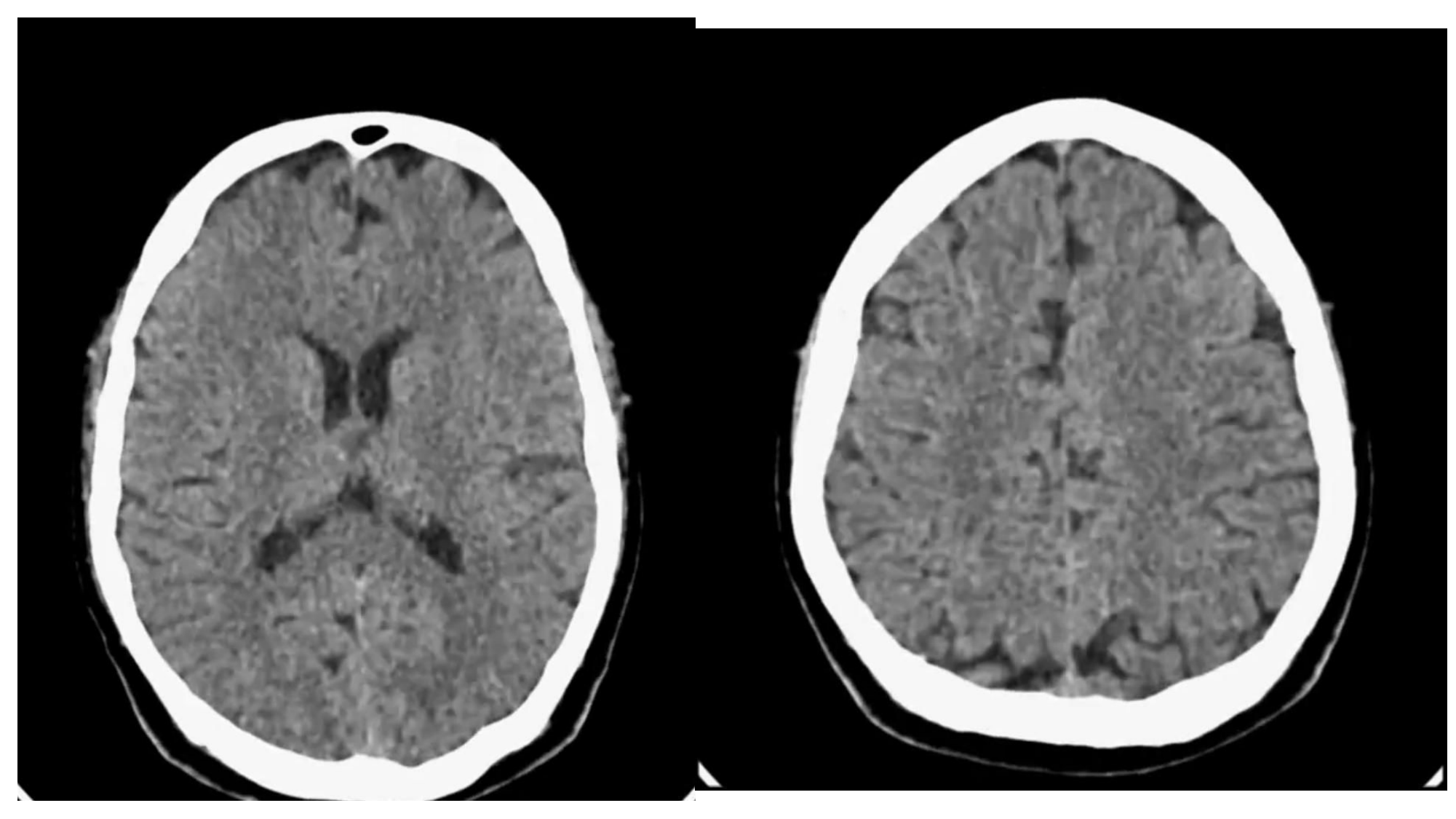

## Slide 10
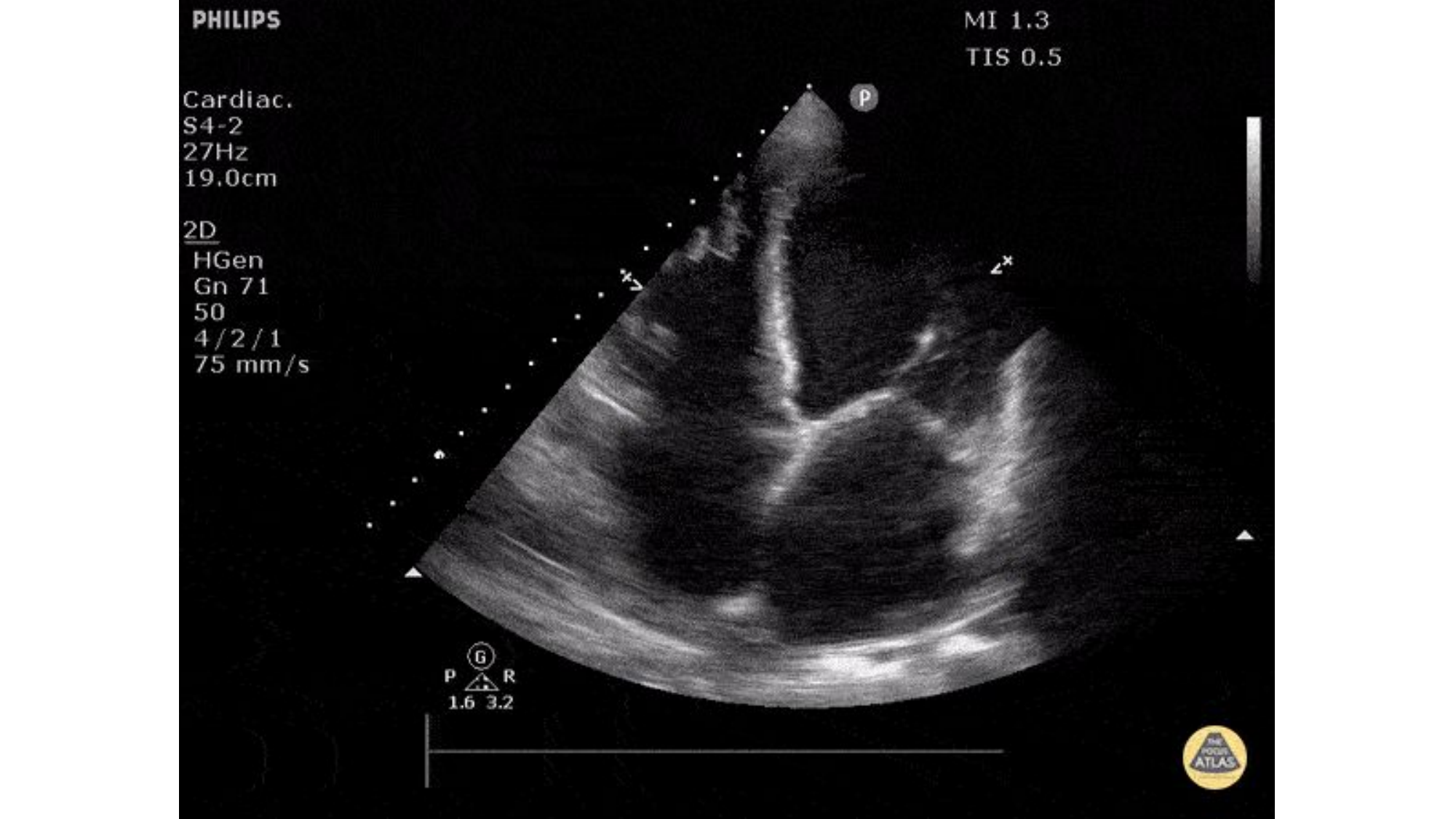

## Slide 11
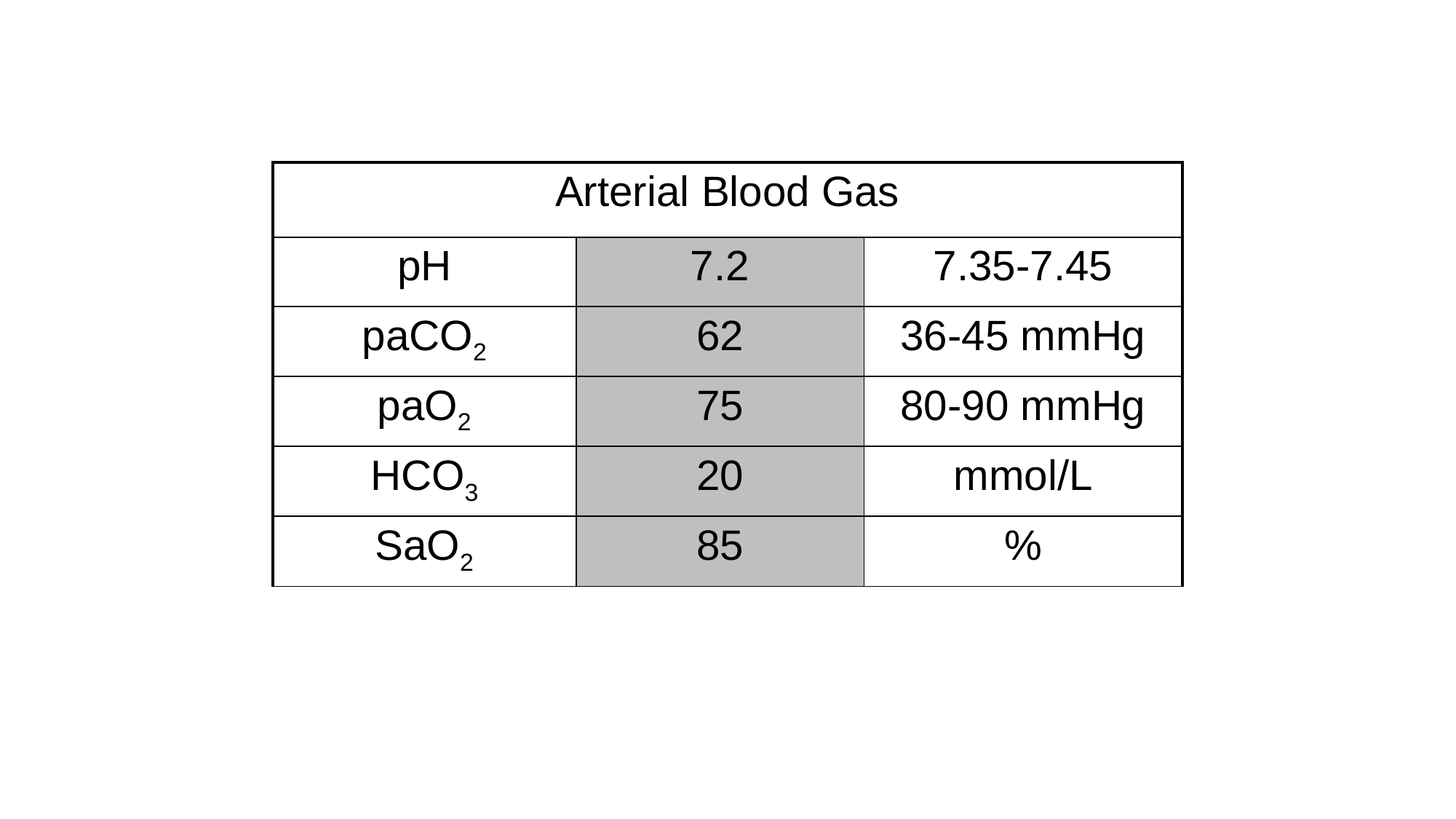

| Arterial Blood Gas | | |
| --- | --- | --- |
| pH | 7.2 | 7.35-7.45 |
| paCO2 | 62 | 36-45 mmHg |
| paO2 | 75 | 80-90 mmHg |
| HCO3 | 20 | mmol/L |
| SaO2 | 85 | % |

## Slide 12
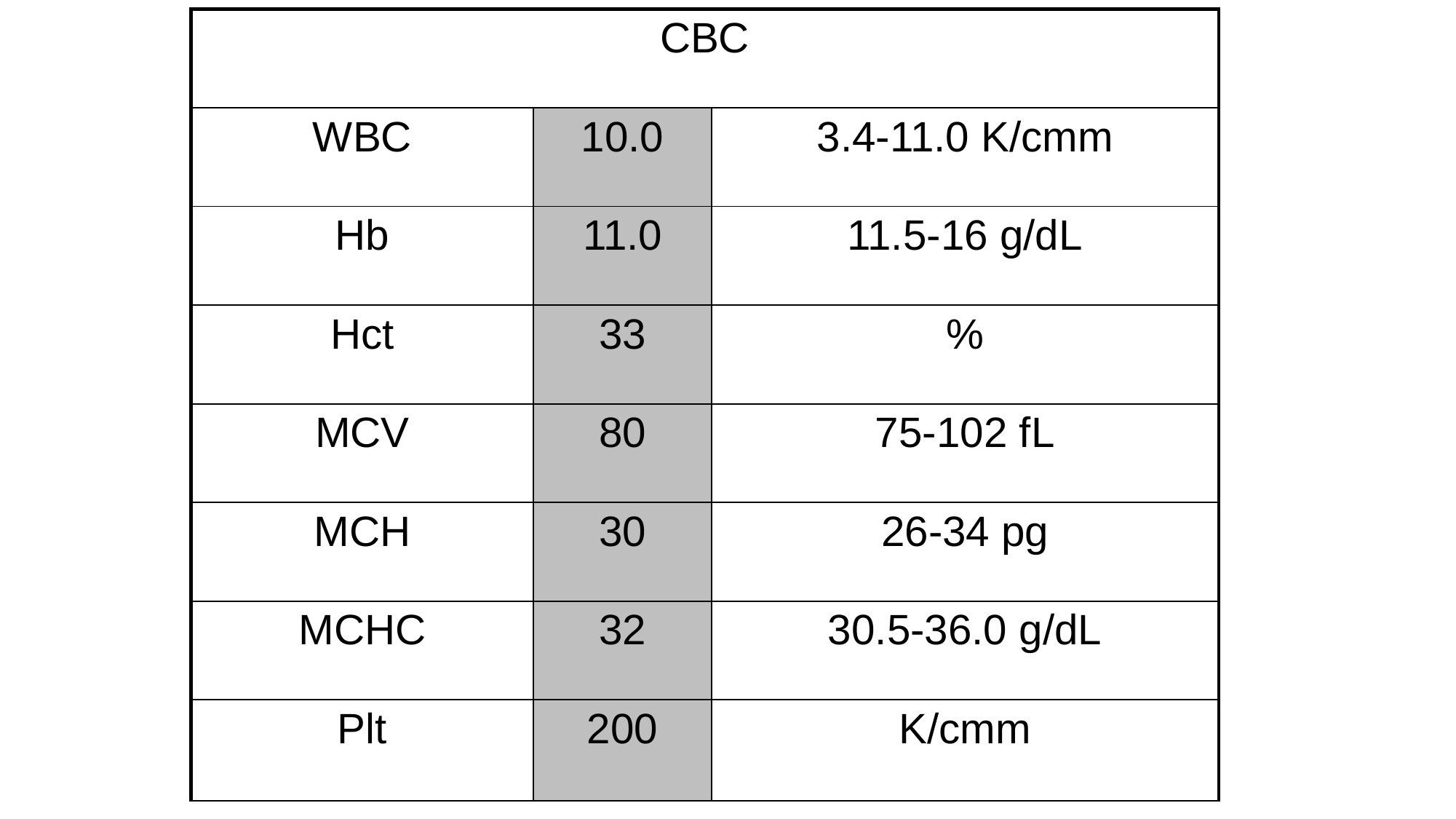

| CBC | | |
| --- | --- | --- |
| WBC | 10.0 | 3.4-11.0 K/cmm |
| Hb | 11.0 | 11.5-16 g/dL |
| Hct | 33 | % |
| MCV | 80 | 75-102 fL |
| MCH | 30 | 26-34 pg |
| MCHC | 32 | 30.5-36.0 g/dL |
| Plt | 200 | K/cmm |
#

## Slide 13
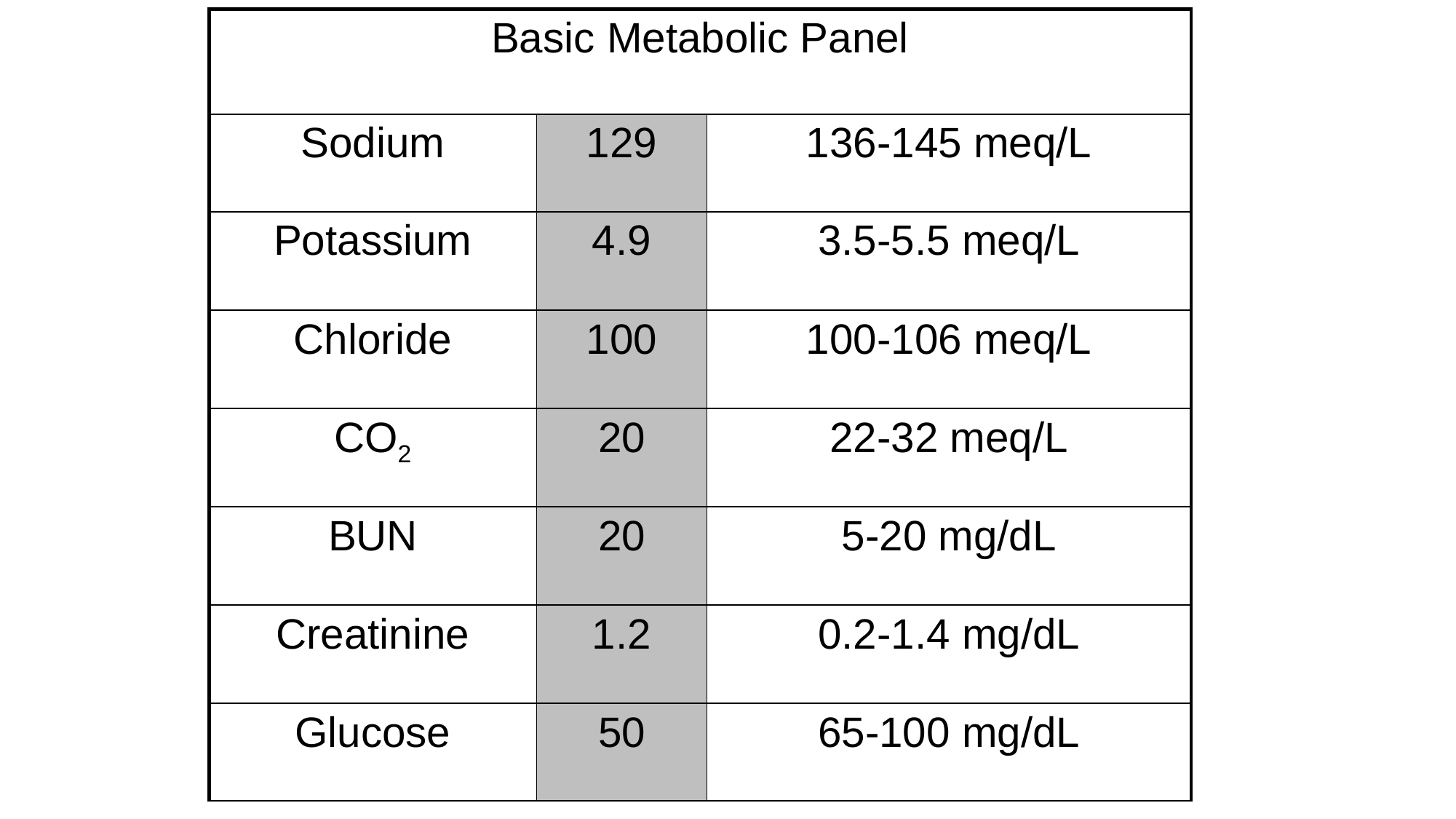

| Basic Metabolic Panel | | |
| --- | --- | --- |
| Sodium | 129 | 136-145 meq/L |
| Potassium | 4.9 | 3.5-5.5 meq/L |
| Chloride | 100 | 100-106 meq/L |
| CO2 | 20 | 22-32 meq/L |
| BUN | 20 | 5-20 mg/dL |
| Creatinine | 1.2 | 0.2-1.4 mg/dL |
| Glucose | 50 | 65-100 mg/dL |
#

## Slide 14
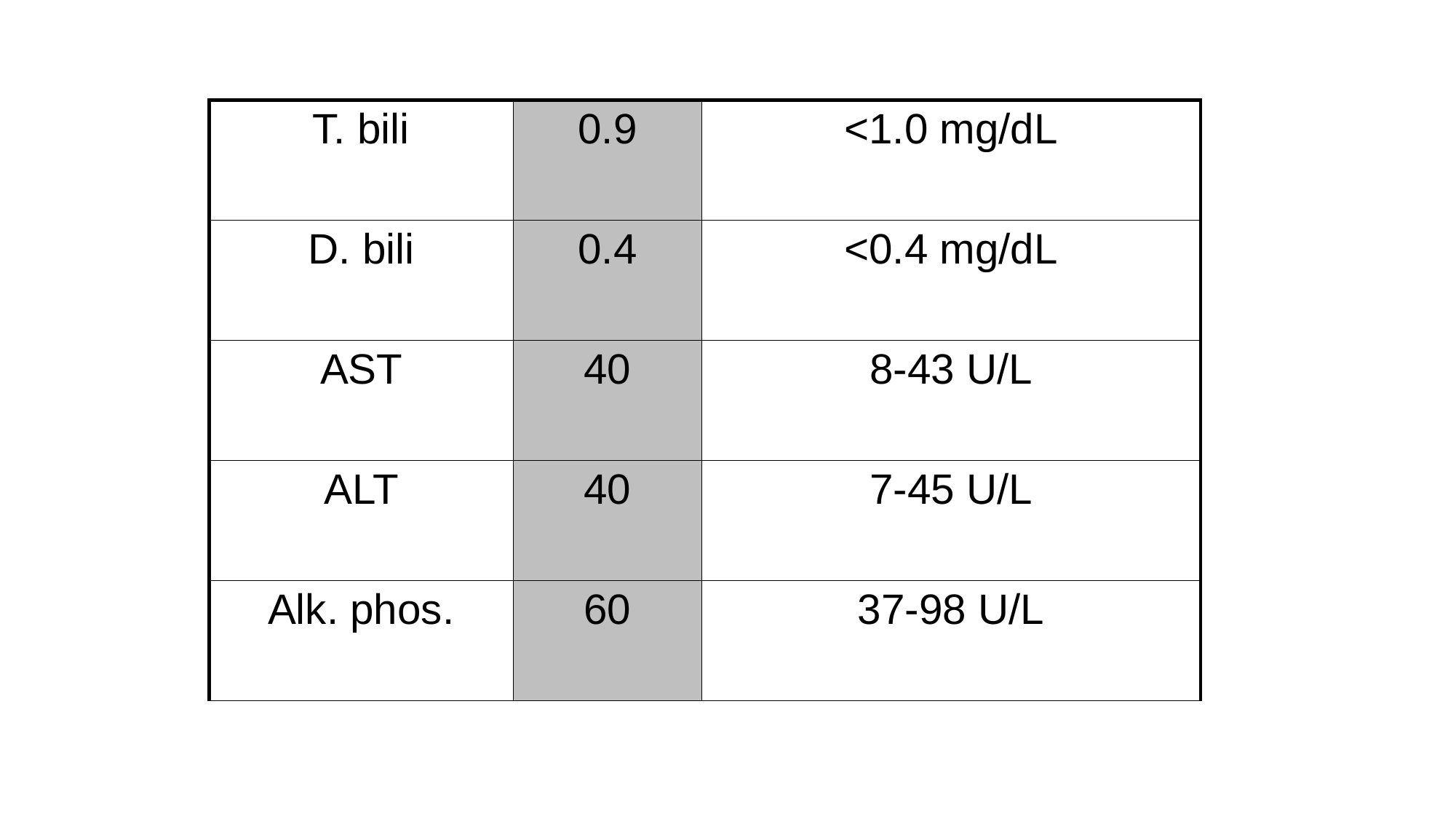

| T. bili | 0.9 | <1.0 mg/dL |
| --- | --- | --- |
| D. bili | 0.4 | <0.4 mg/dL |
| AST | 40 | 8-43 U/L |
| ALT | 40 | 7-45 U/L |
| Alk. phos. | 60 | 37-98 U/L |

## Slide 15
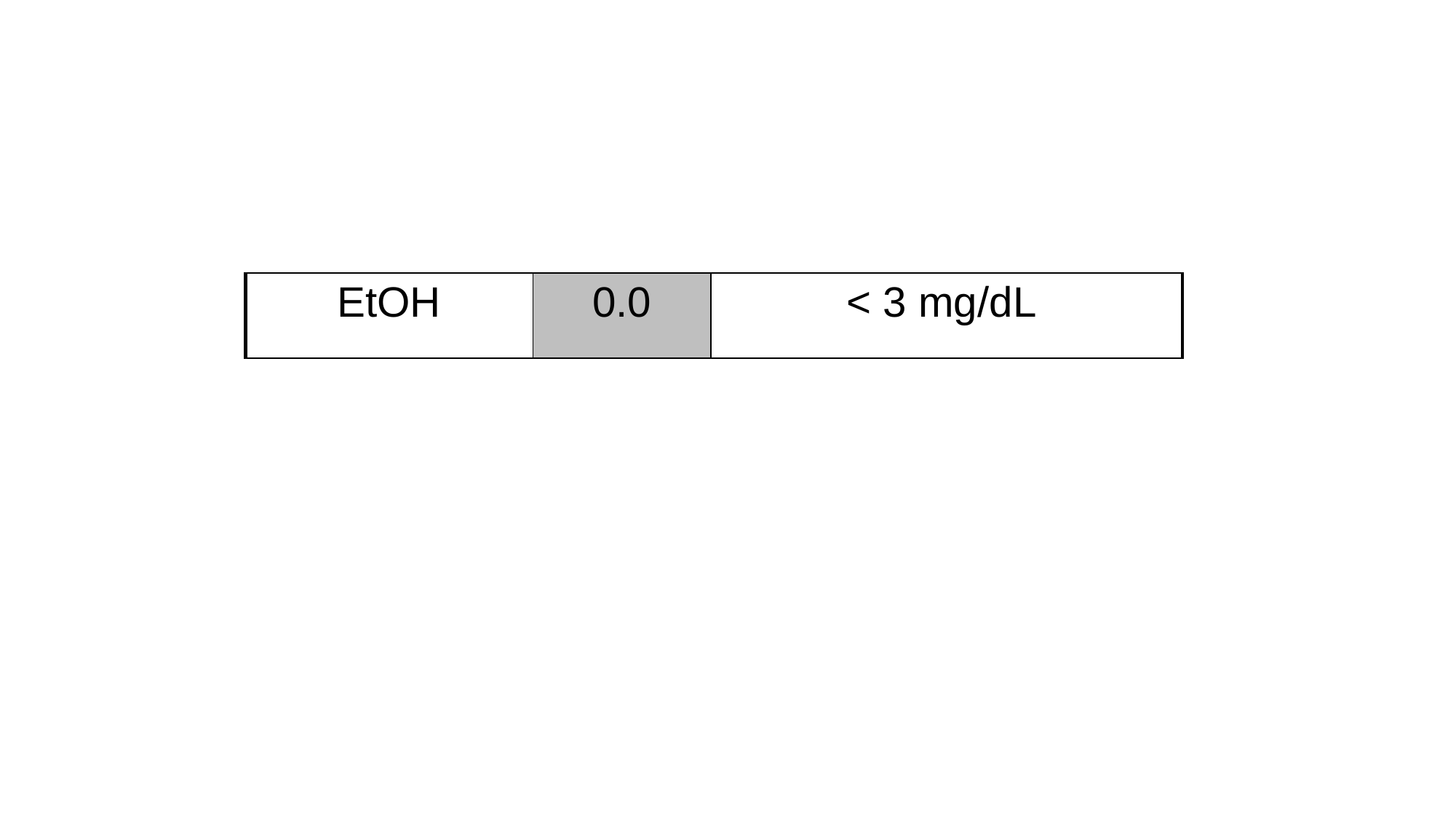

| EtOH | 0.0 | < 3 mg/dL |
| --- | --- | --- |

## Slide 16
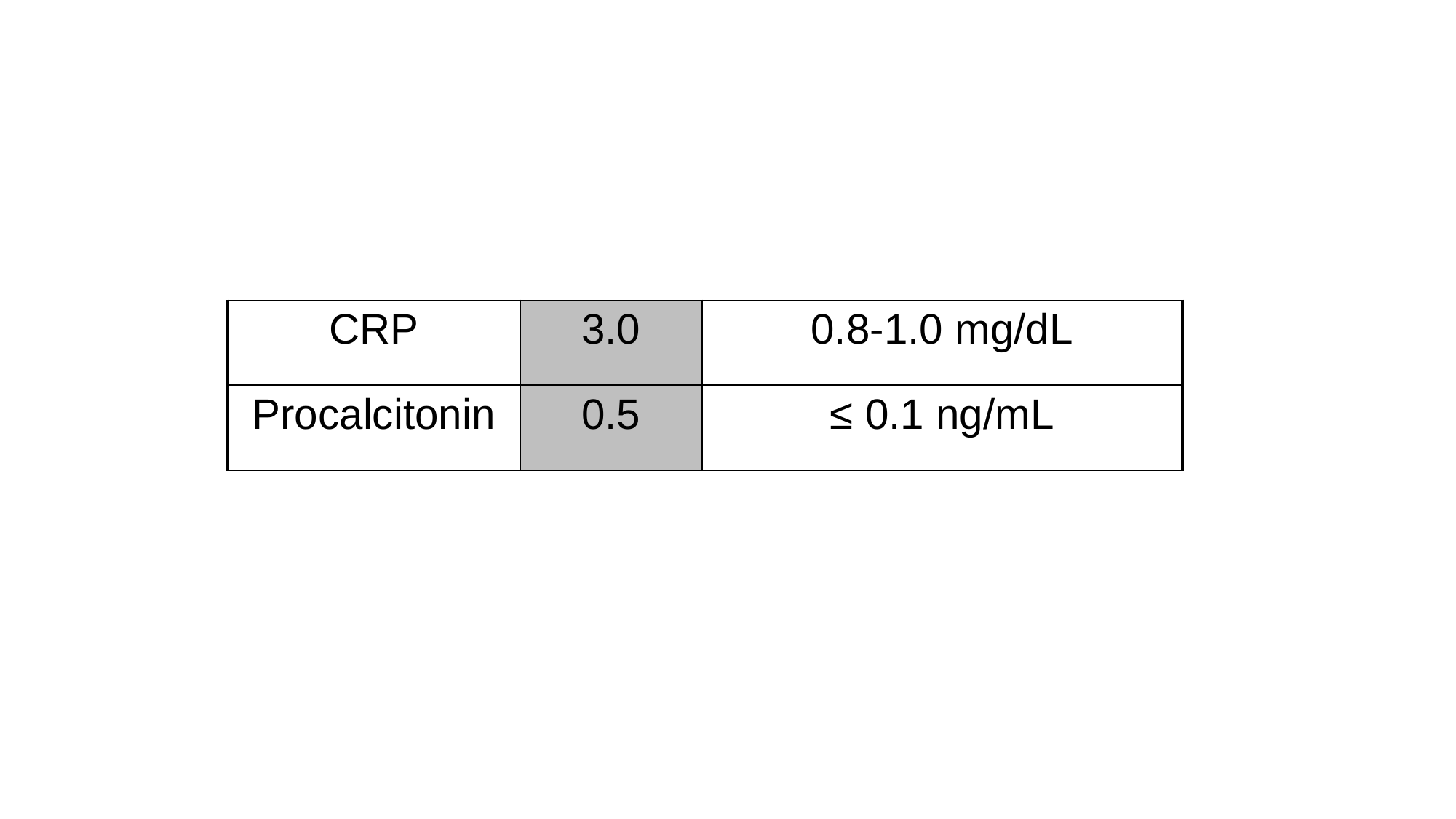

| CRP | 3.0 | 0.8-1.0 mg/dL |
| --- | --- | --- |
| Procalcitonin | 0.5 | ≤ 0.1 ng/mL |

## Slide 17
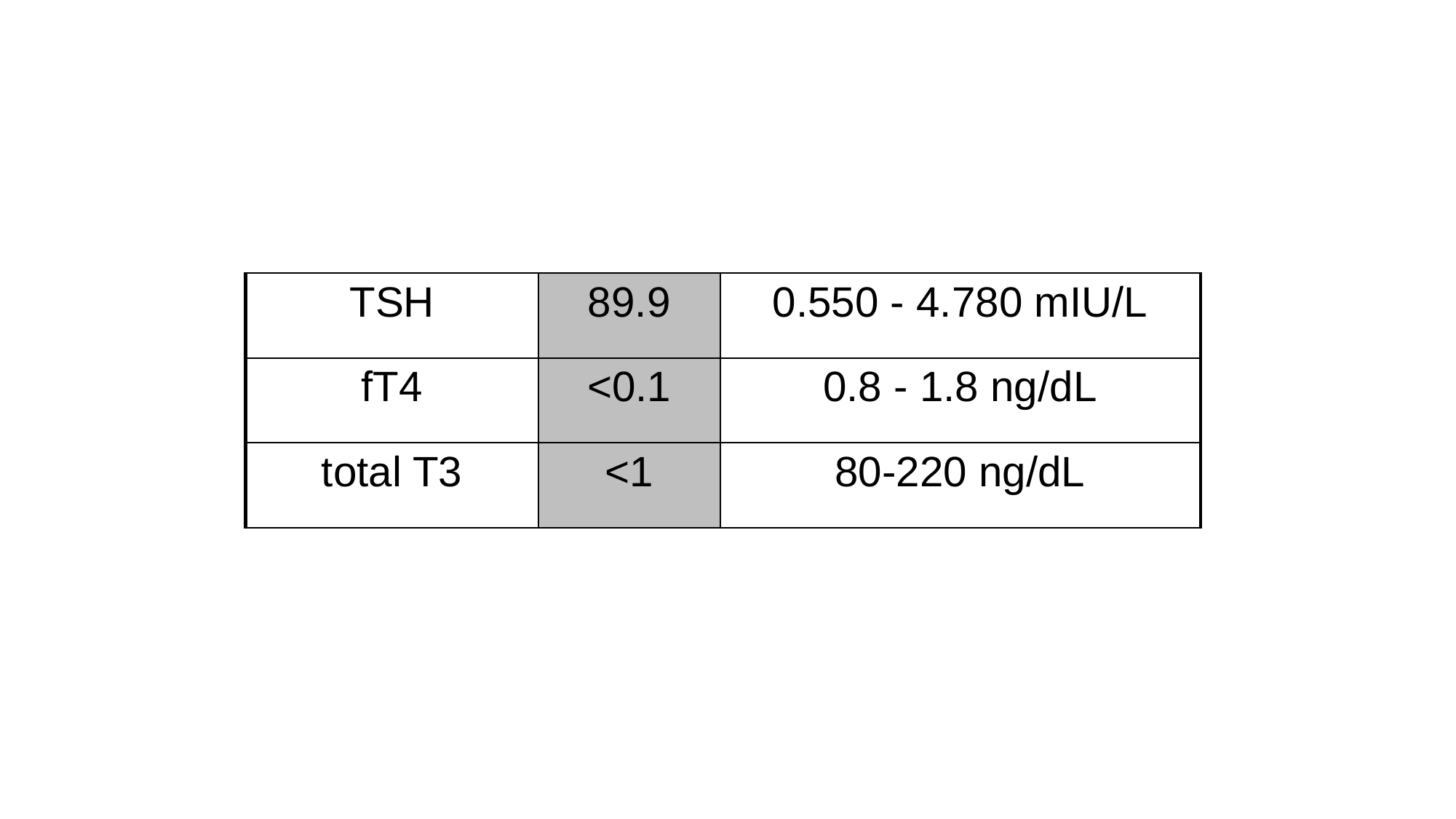

| TSH | 89.9 | 0.550 - 4.780 mIU/L |
| --- | --- | --- |
| fT4 | <0.1 | 0.8 - 1.8 ng/dL |
| total T3 | <1 | 80-220 ng/dL |

## Slide 18
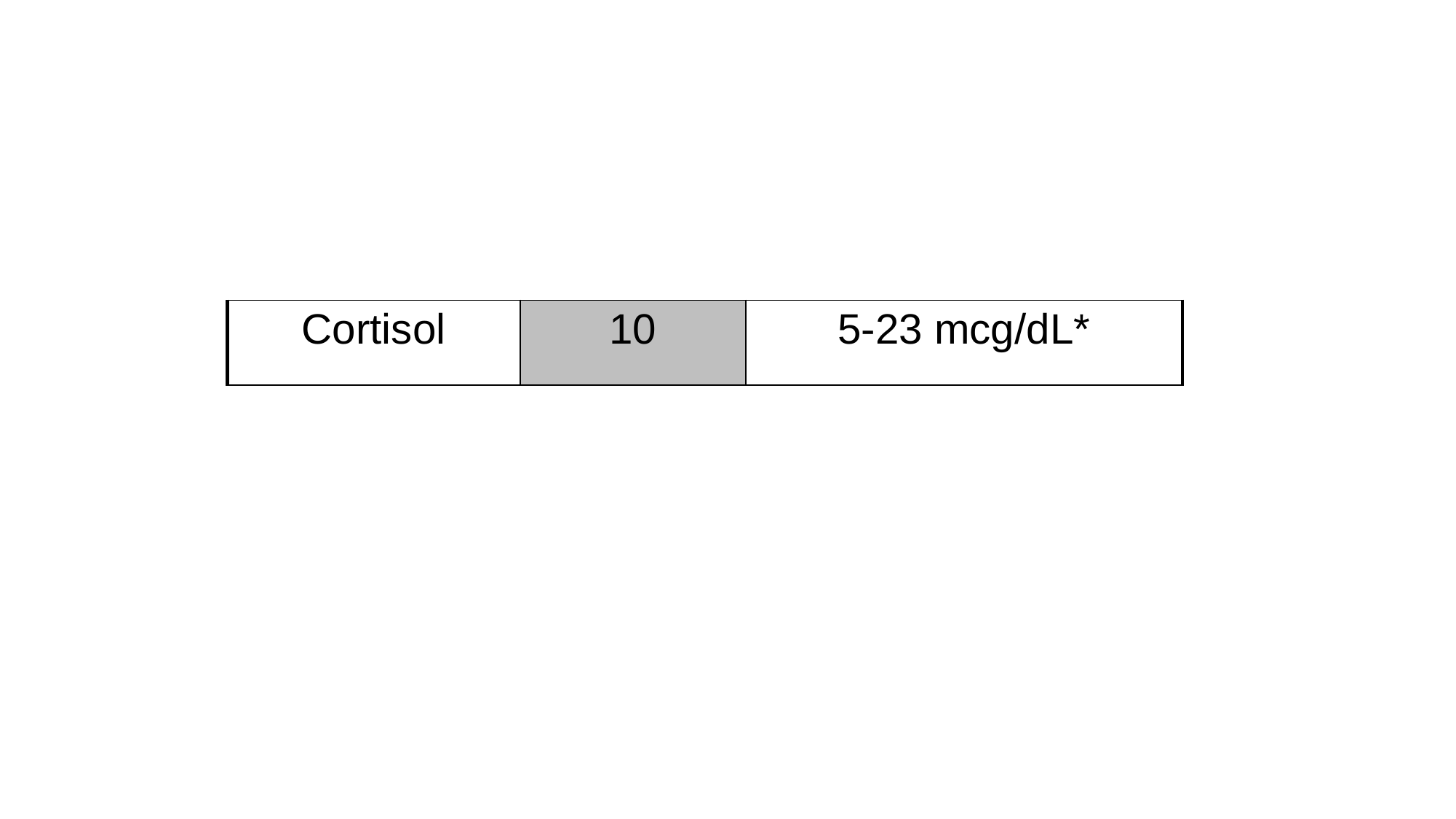

| Cortisol | 10 | 5-23 mcg/dL\* |
| --- | --- | --- |

## Slide 19
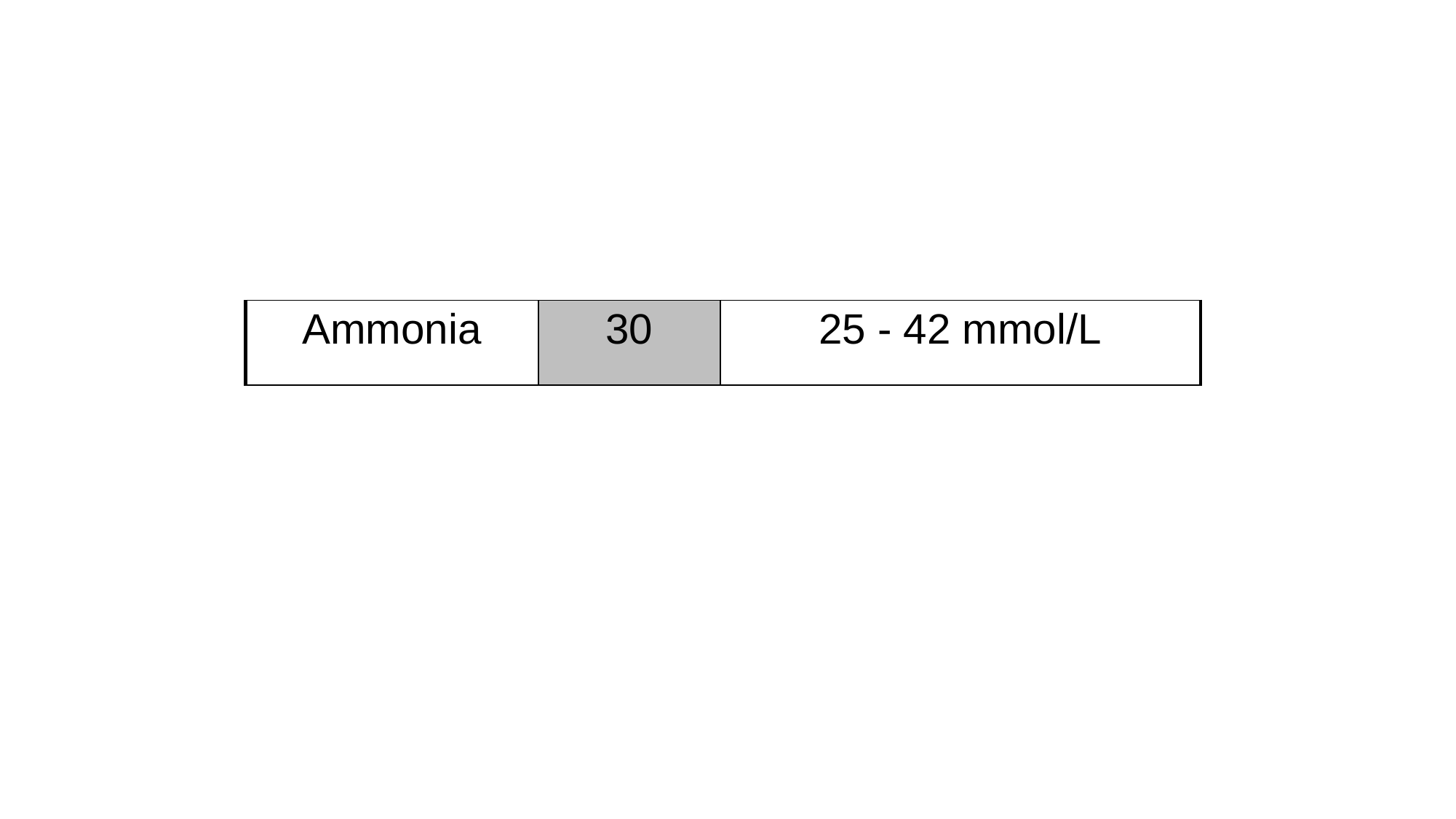

| Ammonia | 30 | 25 - 42 mmol/L |
| --- | --- | --- |

## Slide 20
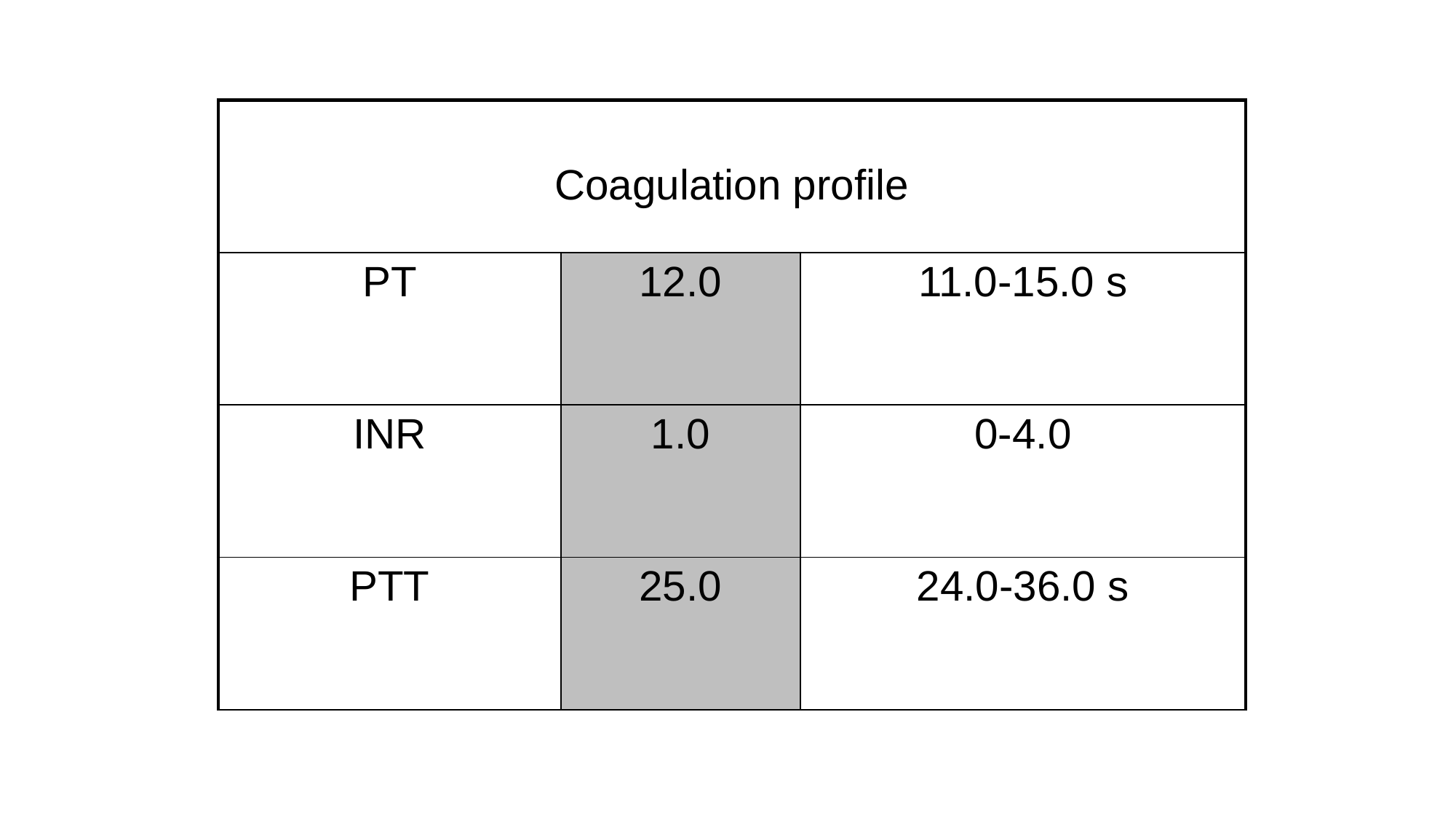

| Coagulation profile | | |
| --- | --- | --- |
| PT | 12.0 | 11.0-15.0 s |
| INR | 1.0 | 0-4.0 |
| PTT | 25.0 | 24.0-36.0 s |

## Slide 21
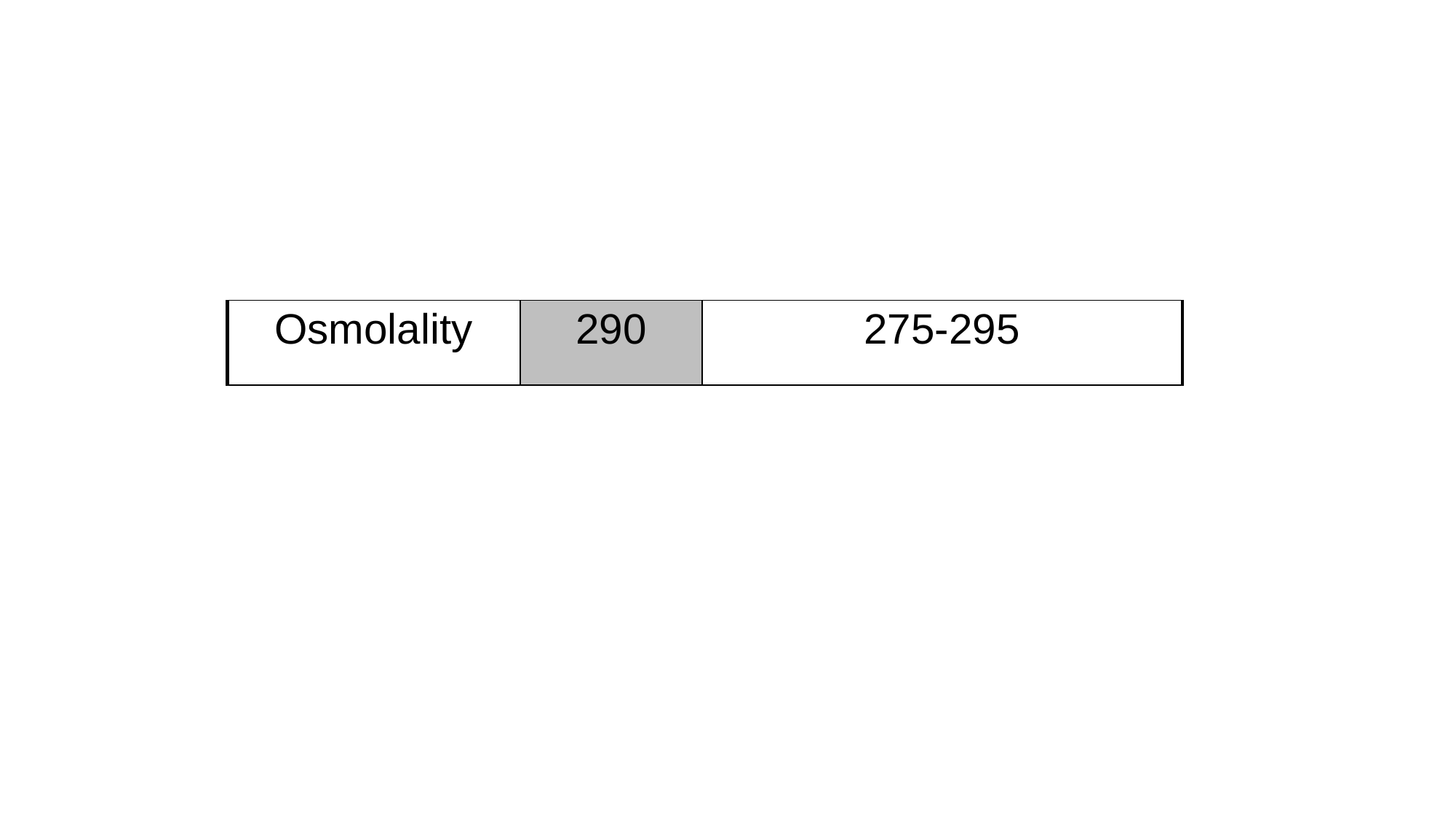

| Osmolality | 290 | 275-295 |
| --- | --- | --- |

## Slide 22
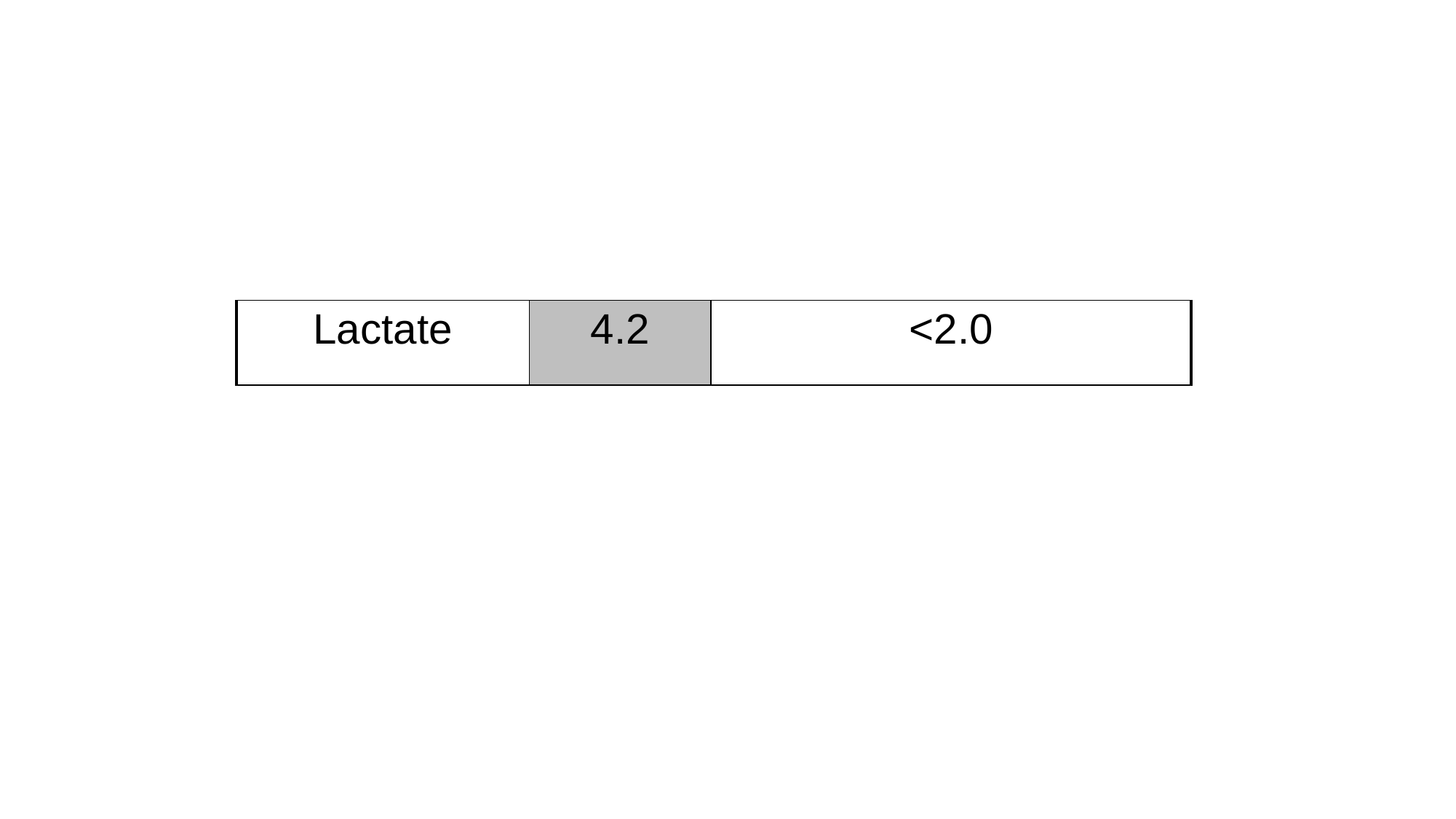

| Lactate | 4.2 | <2.0 |
| --- | --- | --- |

## Slide 23
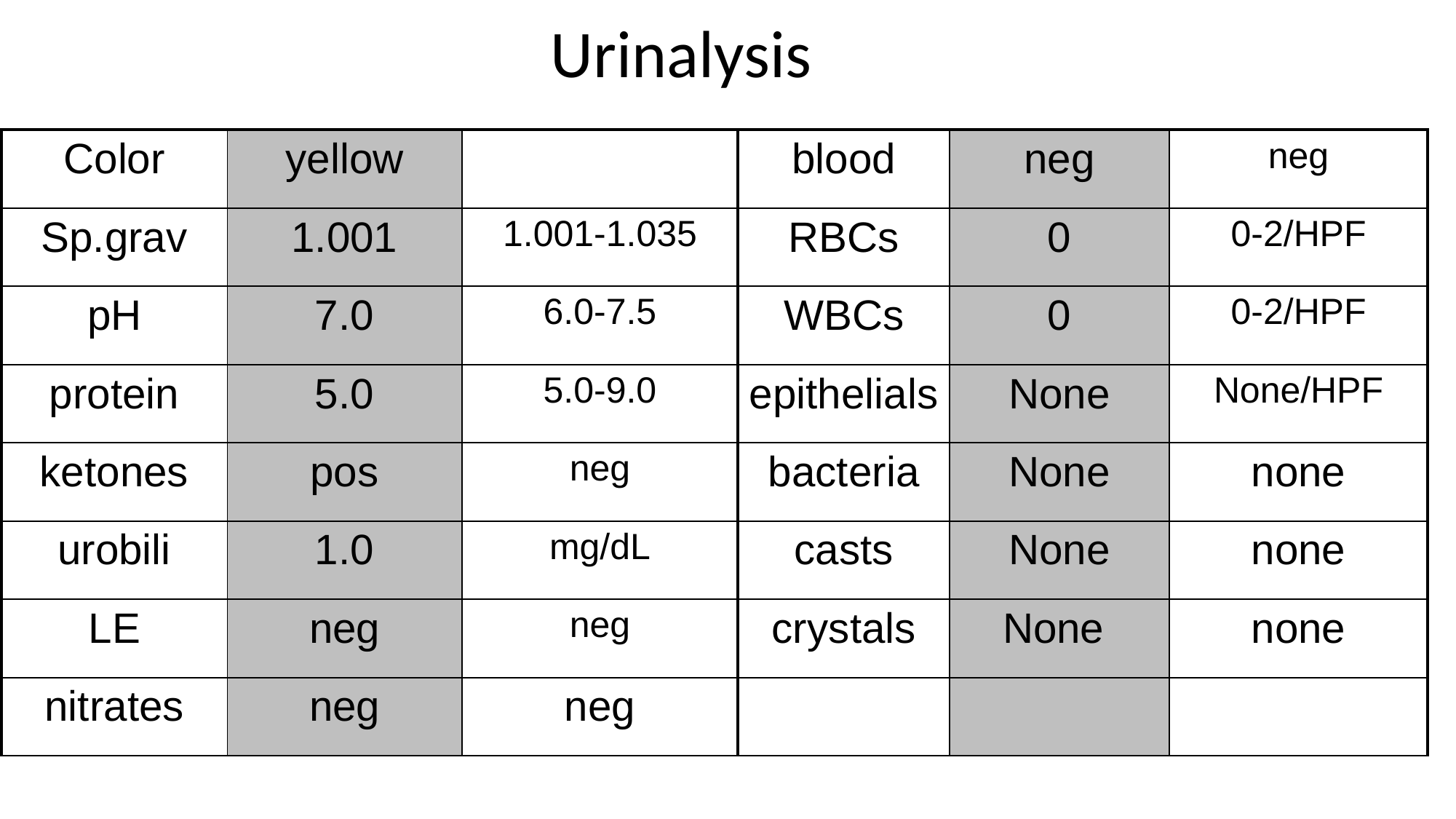

Urinalysis
| Color | yellow | |
| --- | --- | --- |
| Sp.grav | 1.001 | 1.001-1.035 |
| pH | 7.0 | 6.0-7.5 |
| protein | 5.0 | 5.0-9.0 |
| ketones | pos | neg |
| urobili | 1.0 | mg/dL |
| LE | neg | neg |
| nitrates | neg | neg |
| blood | neg | neg |
| --- | --- | --- |
| RBCs | 0 | 0-2/HPF |
| WBCs | 0 | 0-2/HPF |
| epithelials | None | None/HPF |
| bacteria | None | none |
| casts | None | none |
| crystals | None | none |
| | | |

## Slide 24
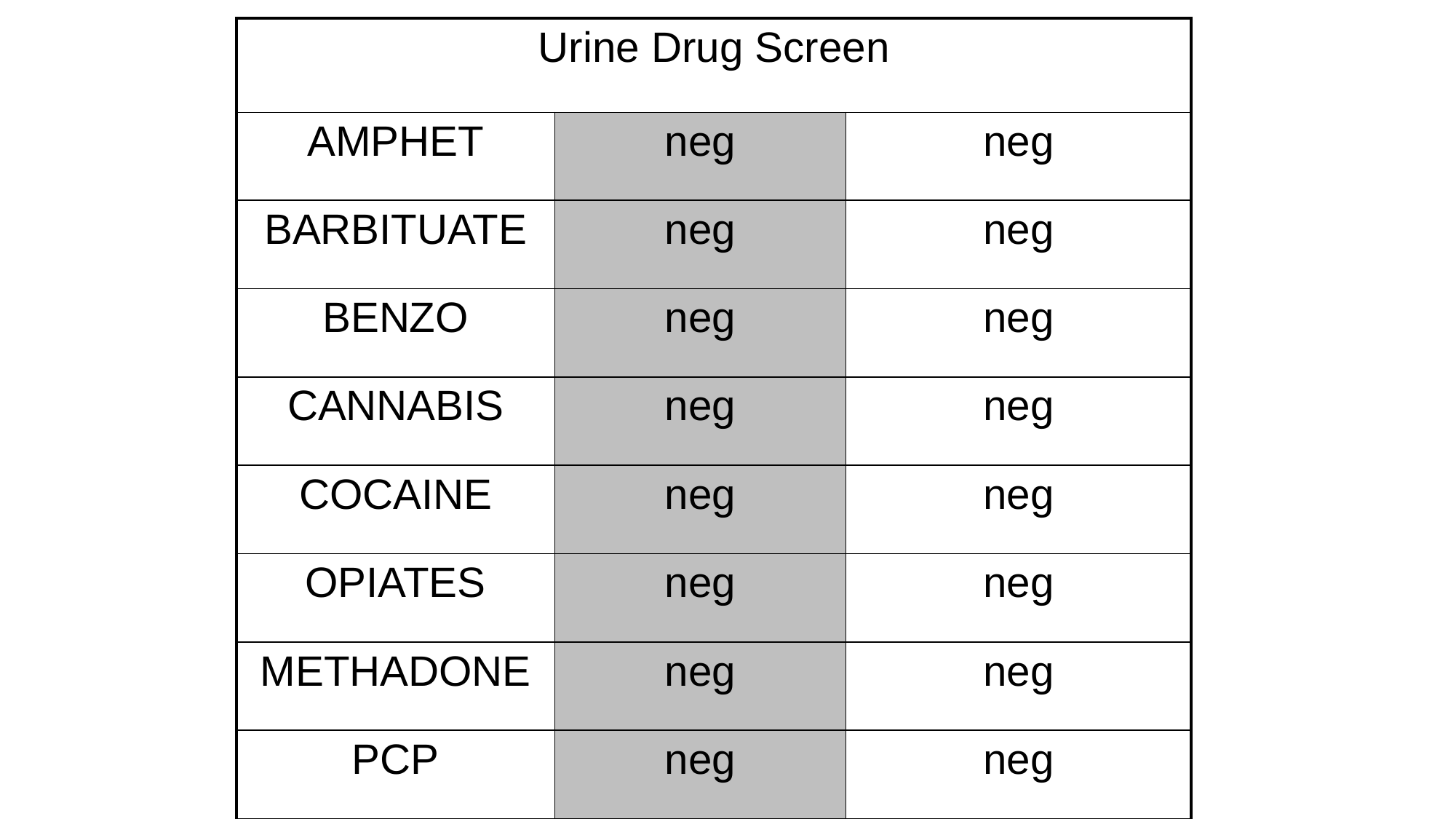

| Urine Drug Screen | | |
| --- | --- | --- |
| AMPHET | neg | neg |
| BARBITUATE | neg | neg |
| BENZO | neg | neg |
| CANNABIS | neg | neg |
| COCAINE | neg | neg |
| OPIATES | neg | neg |
| METHADONE | neg | neg |
| PCP | neg | neg |

## Slide 25
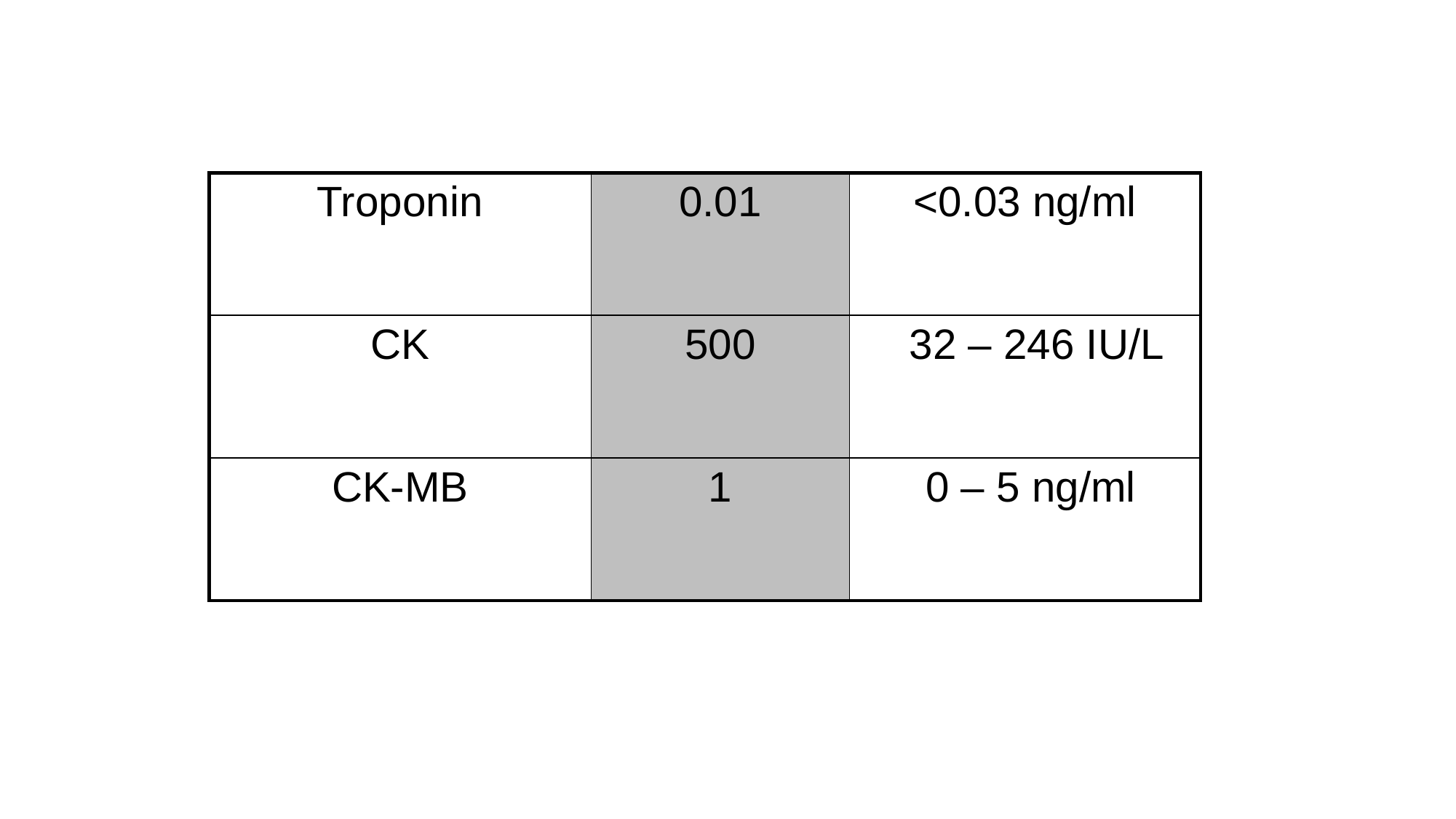

| Troponin | 0.01 | <0.03 ng/ml |
| --- | --- | --- |
| CK | 500 | 32 – 246 IU/L |
| CK-MB | 1 | 0 – 5 ng/ml |

## Slide 26
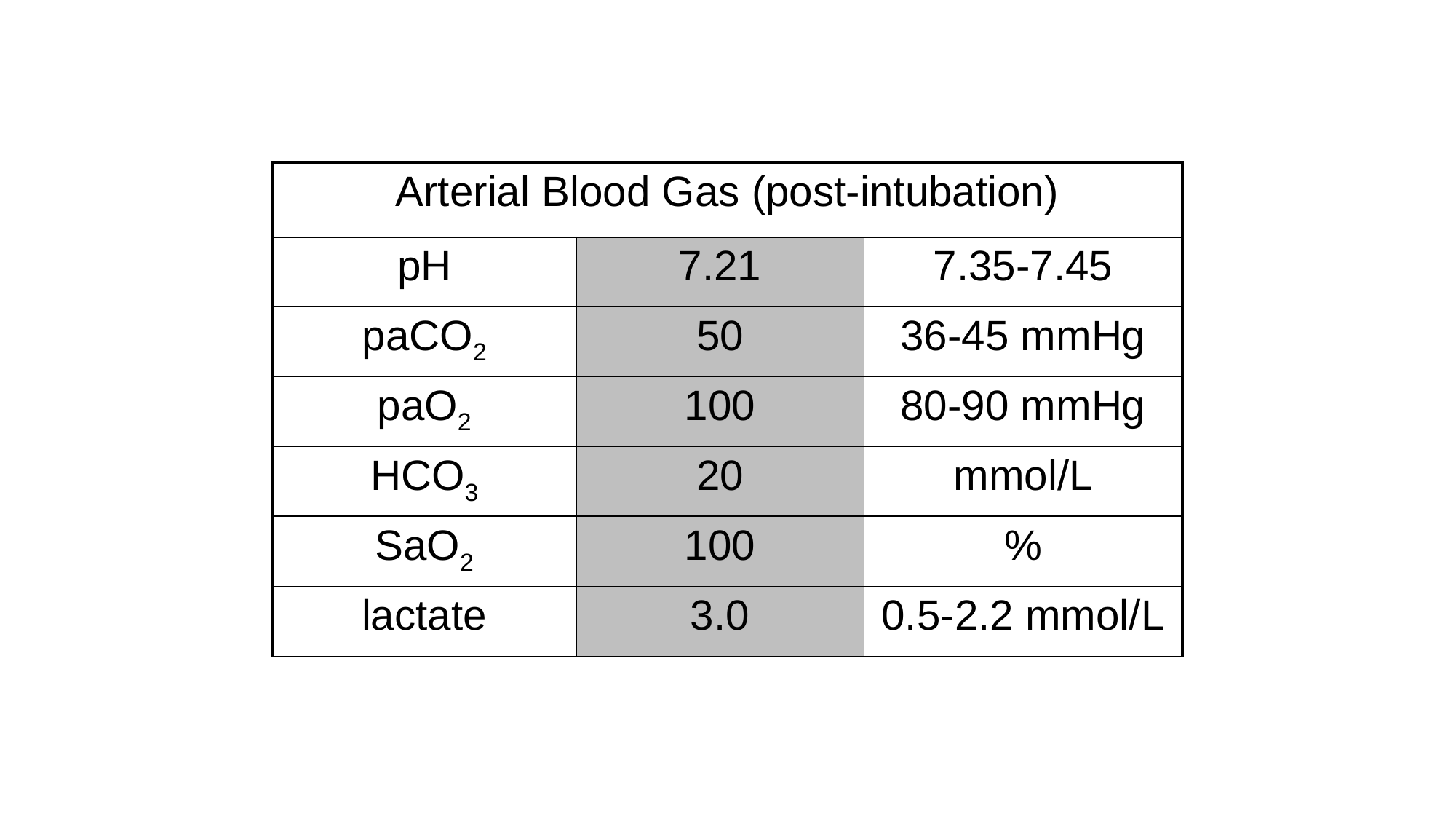

| Arterial Blood Gas (post-intubation) | | |
| --- | --- | --- |
| pH | 7.21 | 7.35-7.45 |
| paCO2 | 50 | 36-45 mmHg |
| paO2 | 100 | 80-90 mmHg |
| HCO3 | 20 | mmol/L |
| SaO2 | 100 | % |
| lactate | 3.0 | 0.5-2.2 mmol/L |

## Slide 27
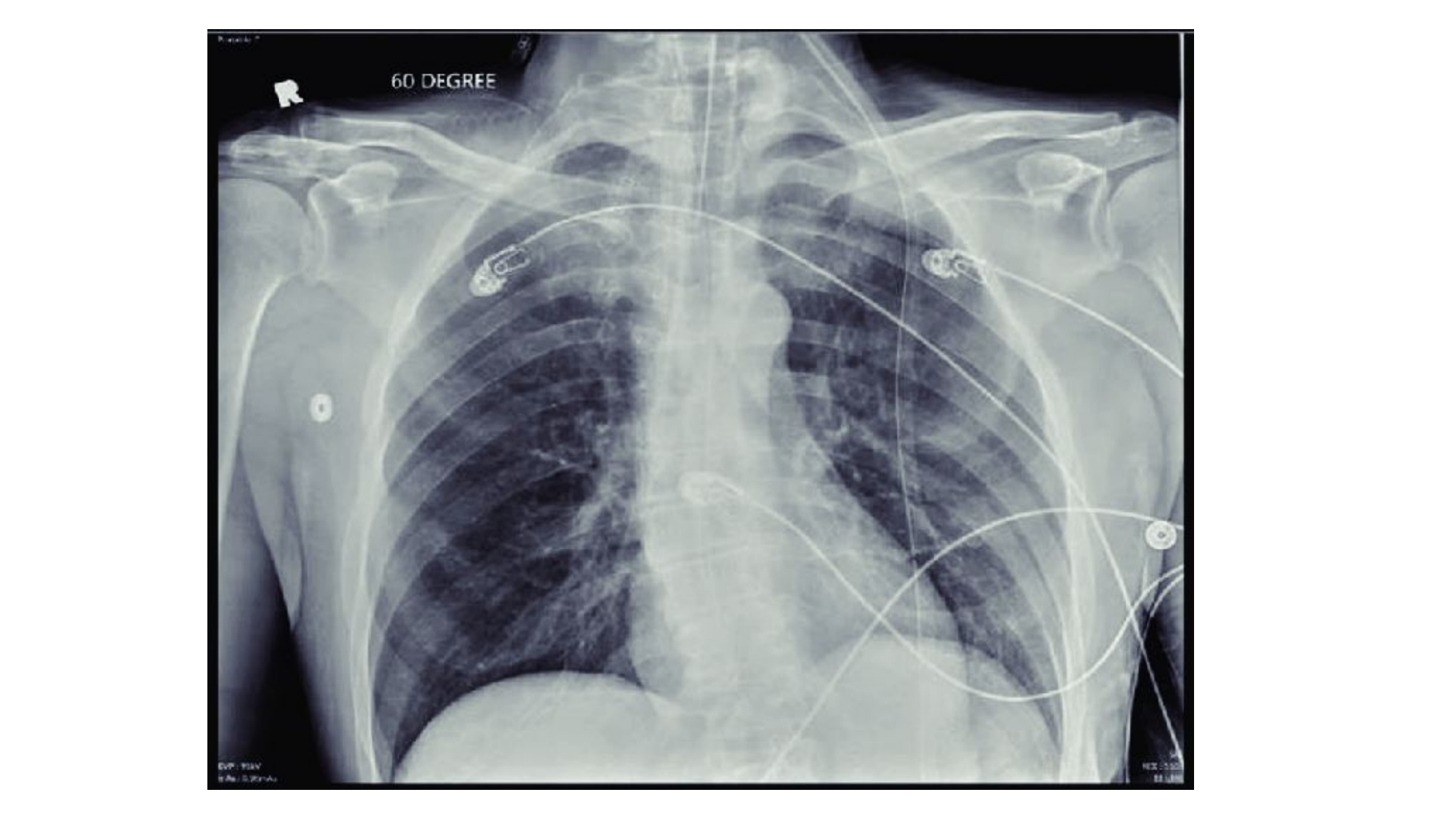

## Slide 28
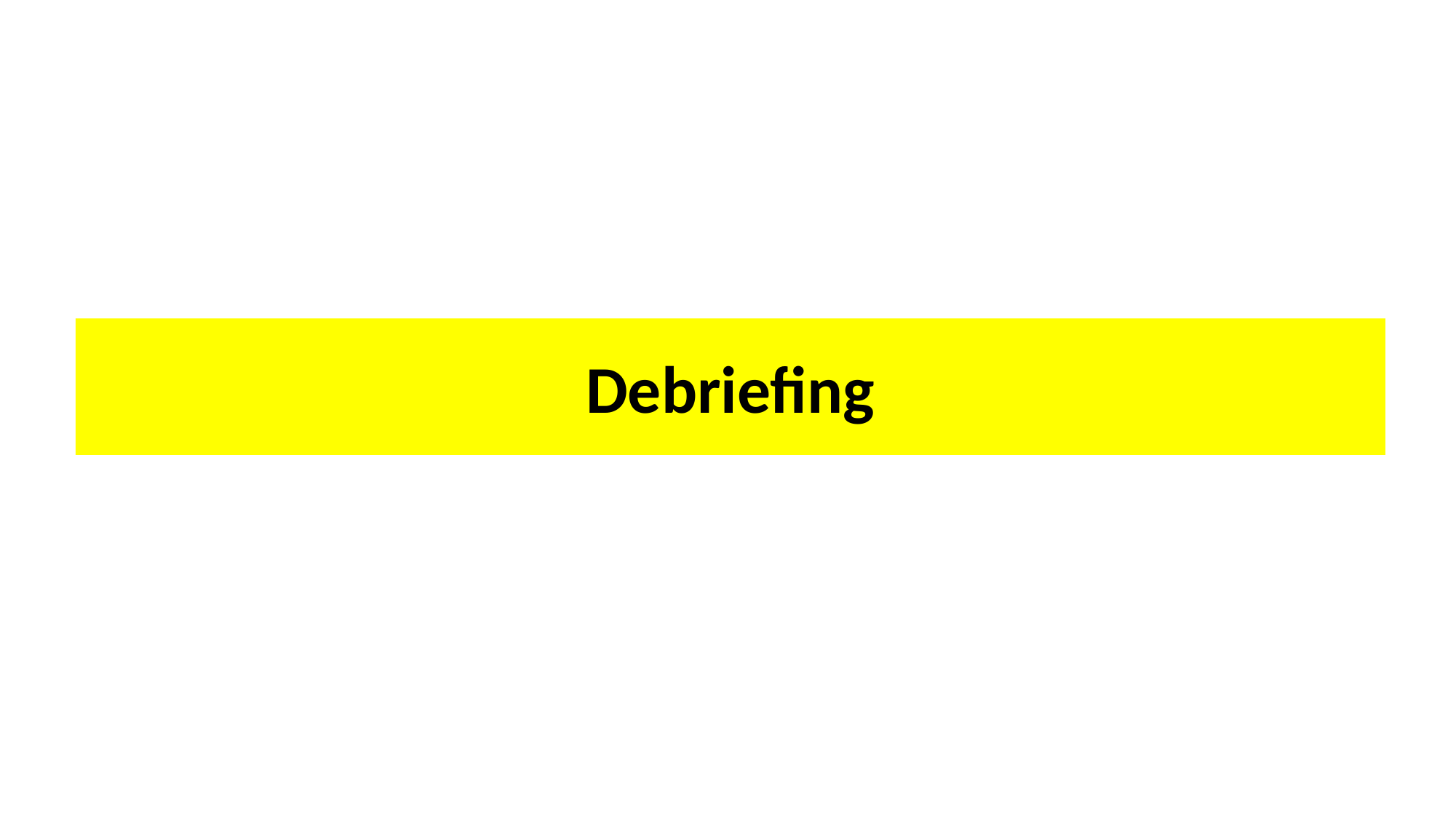

# Debriefing

## Slide 29
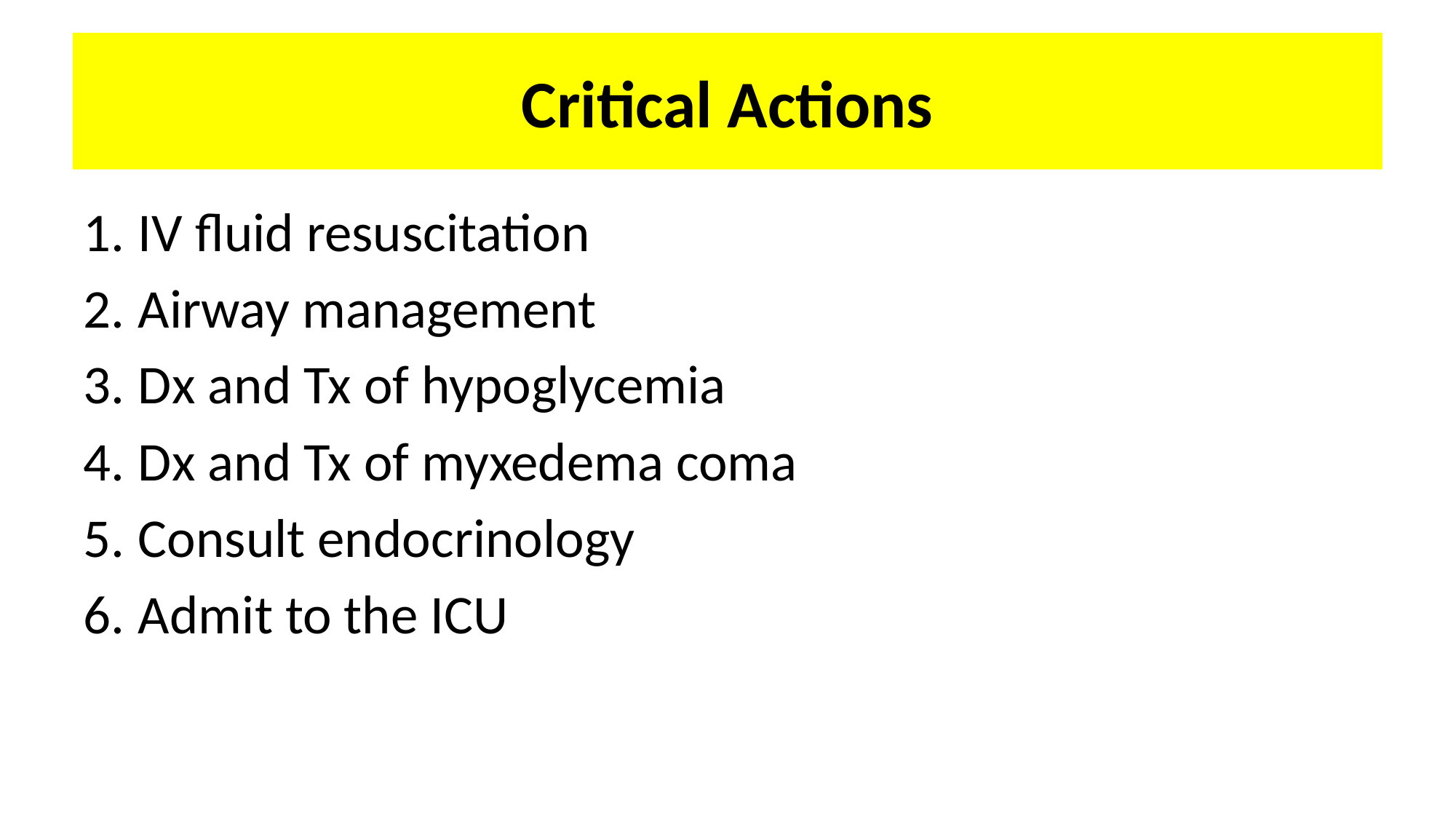

# Critical Actions
IV fluid resuscitation
Airway management
Dx and Tx of hypoglycemia
Dx and Tx of myxedema coma
Consult endocrinology
Admit to the ICU

## Slide 30
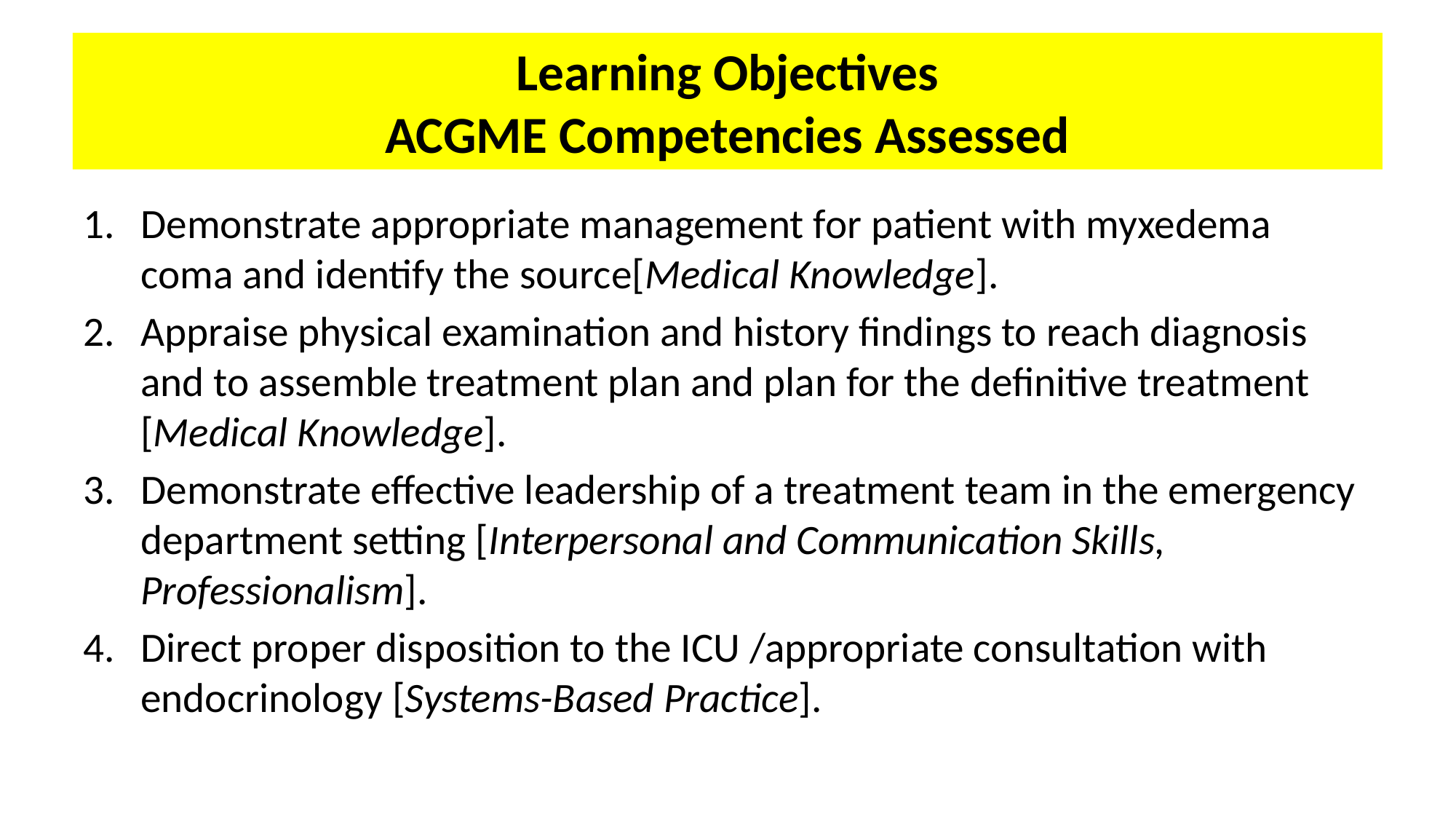

# Learning Objectives ACGME Competencies Assessed
Demonstrate appropriate management for patient with myxedema coma and identify the source[Medical Knowledge].
Appraise physical examination and history findings to reach diagnosis and to assemble treatment plan and plan for the definitive treatment [Medical Knowledge].
Demonstrate effective leadership of a treatment team in the emergency department setting [Interpersonal and Communication Skills, Professionalism].
Direct proper disposition to the ICU /appropriate consultation with endocrinology [Systems-Based Practice].

## Slide 31
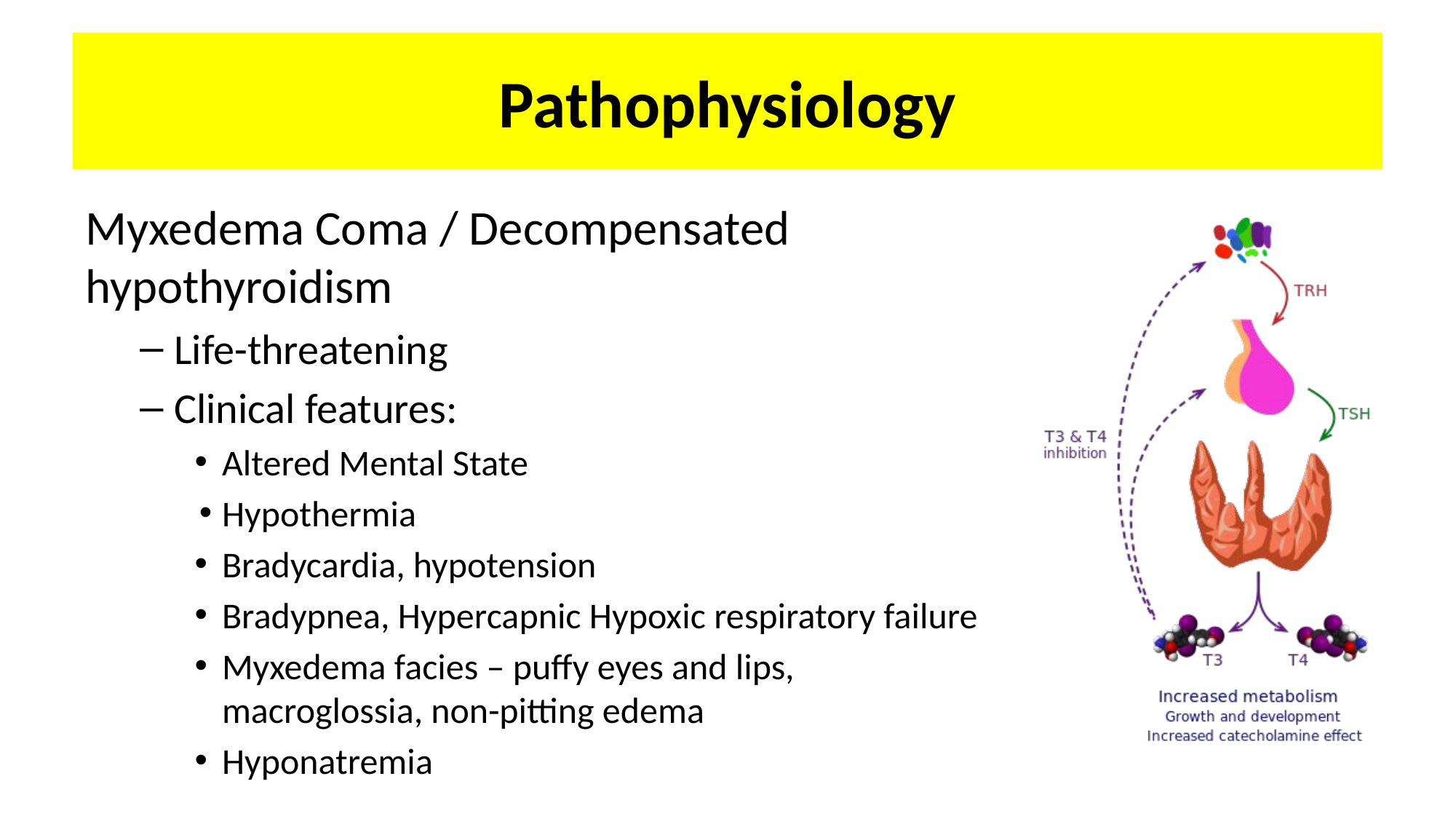

# Pathophysiology
Myxedema Coma / Decompensated hypothyroidism
Life-threatening
Clinical features:
Altered Mental State
Hypothermia
Bradycardia, hypotension
Bradypnea, Hypercapnic Hypoxic respiratory failure
Myxedema facies – puffy eyes and lips, macroglossia, non-pitting edema
Hyponatremia

## Slide 32
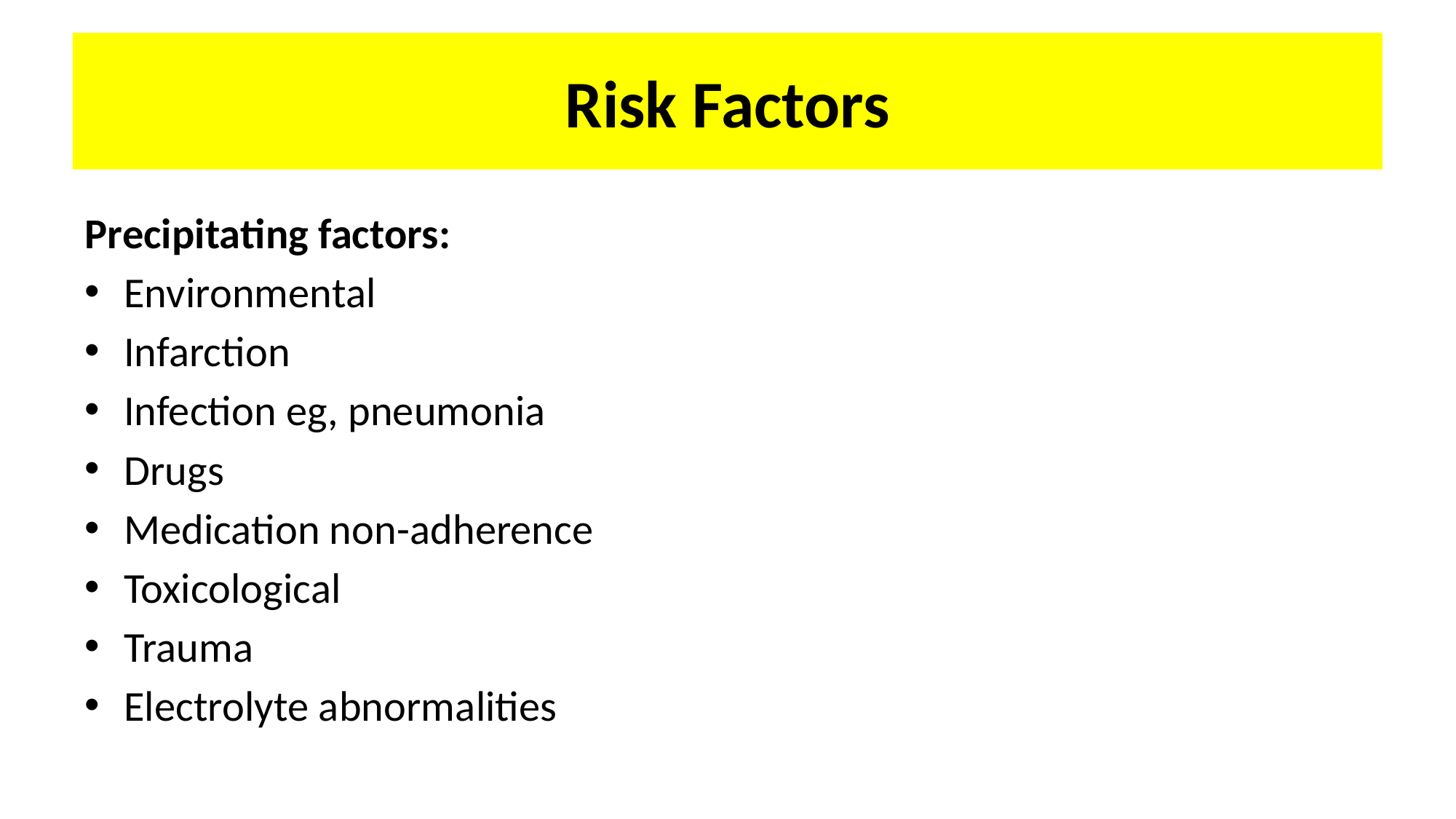

# Risk Factors
Precipitating factors:
Environmental
Infarction
Infection eg, pneumonia
Drugs
Medication non-adherence
Toxicological
Trauma
Electrolyte abnormalities

## Slide 33
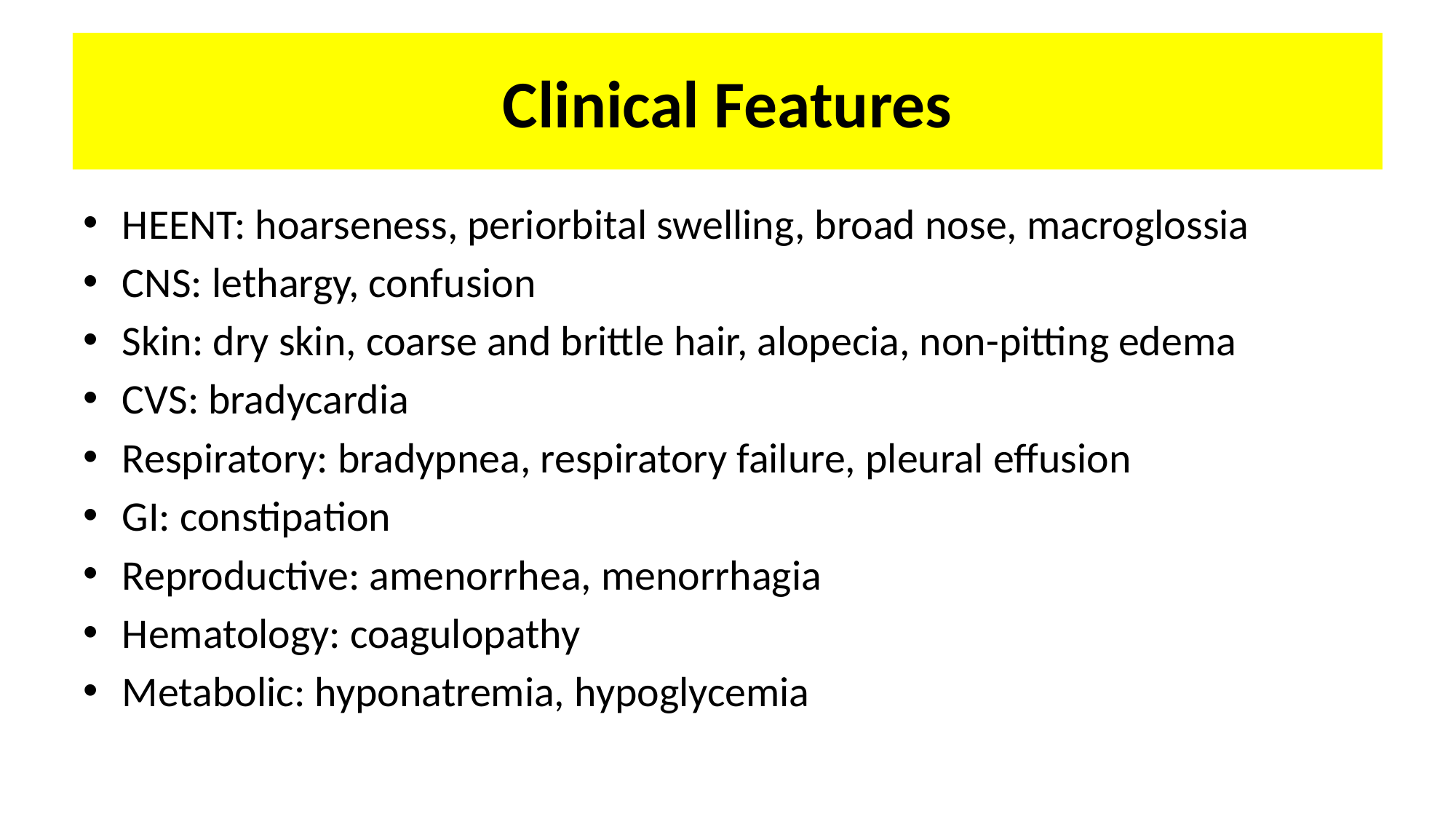

# Clinical Features
HEENT: hoarseness, periorbital swelling, broad nose, macroglossia
CNS: lethargy, confusion
Skin: dry skin, coarse and brittle hair, alopecia, non-pitting edema
CVS: bradycardia
Respiratory: bradypnea, respiratory failure, pleural effusion
GI: constipation
Reproductive: amenorrhea, menorrhagia
Hematology: coagulopathy
Metabolic: hyponatremia, hypoglycemia

## Slide 34
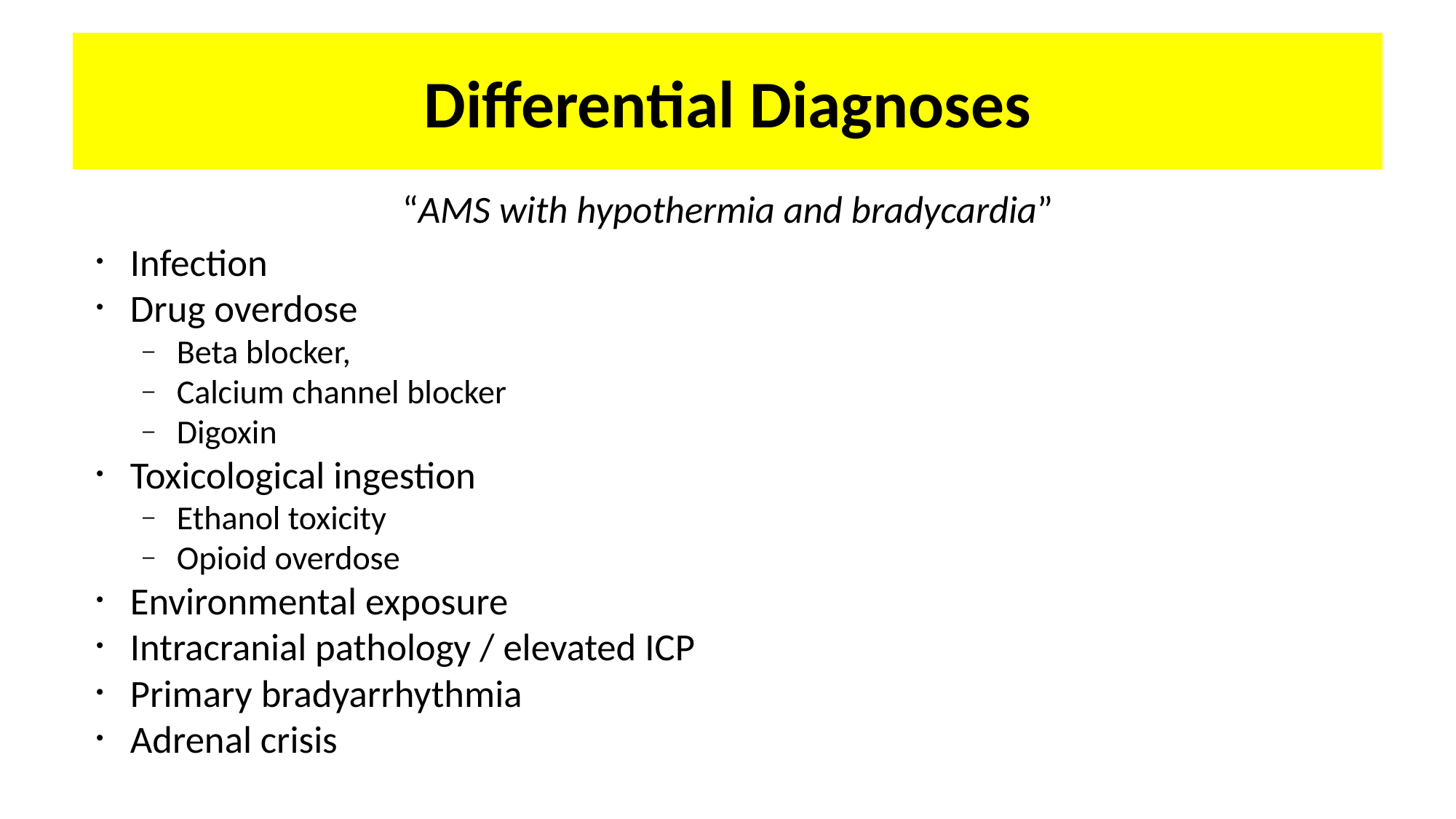

# Differential Diagnoses
“AMS with hypothermia and bradycardia”
Infection
Drug overdose
Beta blocker,
Calcium channel blocker
Digoxin
Toxicological ingestion
Ethanol toxicity
Opioid overdose
Environmental exposure
Intracranial pathology / elevated ICP
Primary bradyarrhythmia
Adrenal crisis

## Slide 35
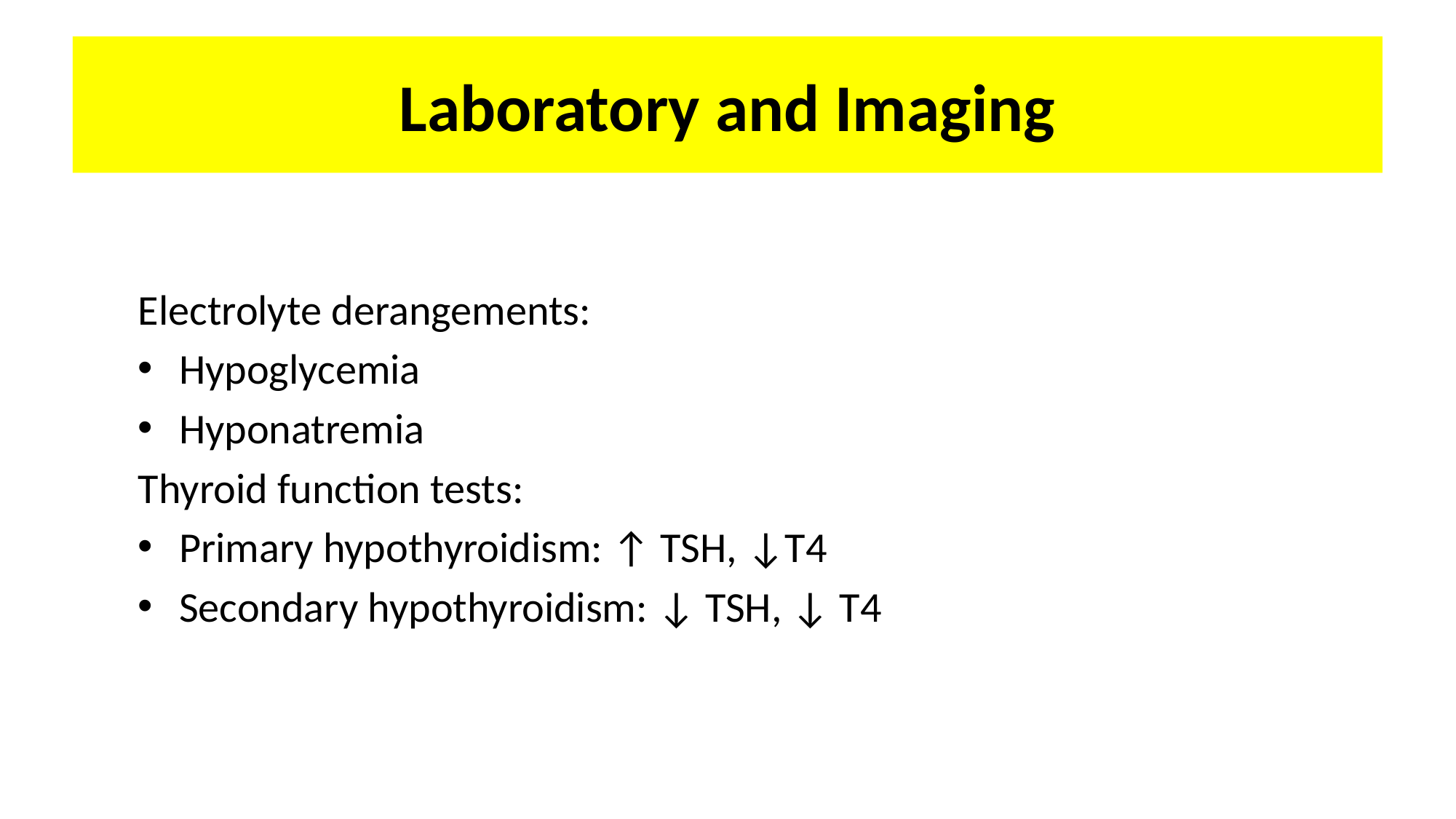

# Laboratory and Imaging
Electrolyte derangements:
Hypoglycemia
Hyponatremia
Thyroid function tests:
Primary hypothyroidism: ↑ TSH, ↓T4
Secondary hypothyroidism: ↓ TSH, ↓ T4

## Slide 36
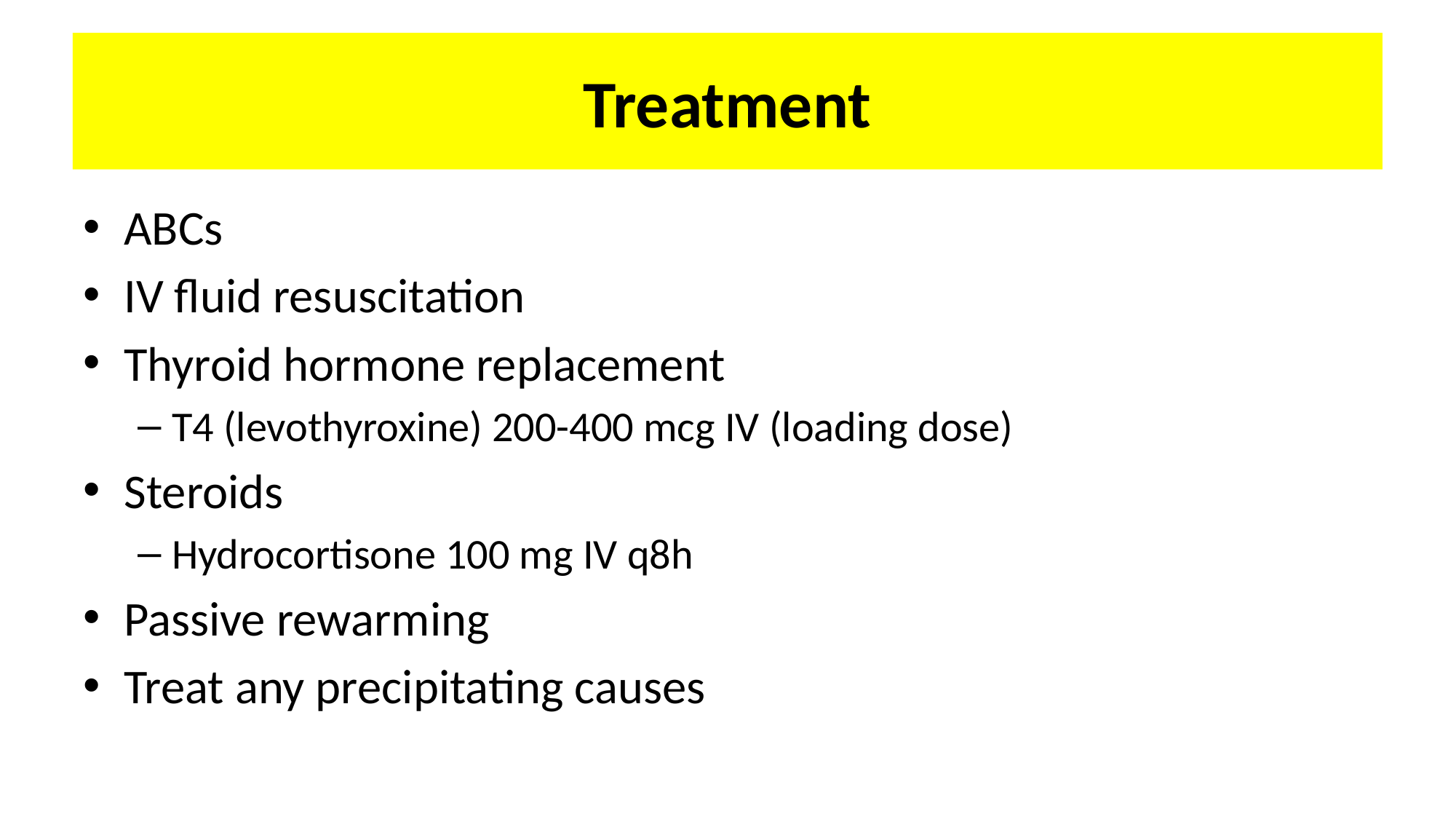

# Treatment
ABCs
IV fluid resuscitation
Thyroid hormone replacement
T4 (levothyroxine) 200-400 mcg IV (loading dose)
Steroids
Hydrocortisone 100 mg IV q8h
Passive rewarming
Treat any precipitating causes

## Slide 37
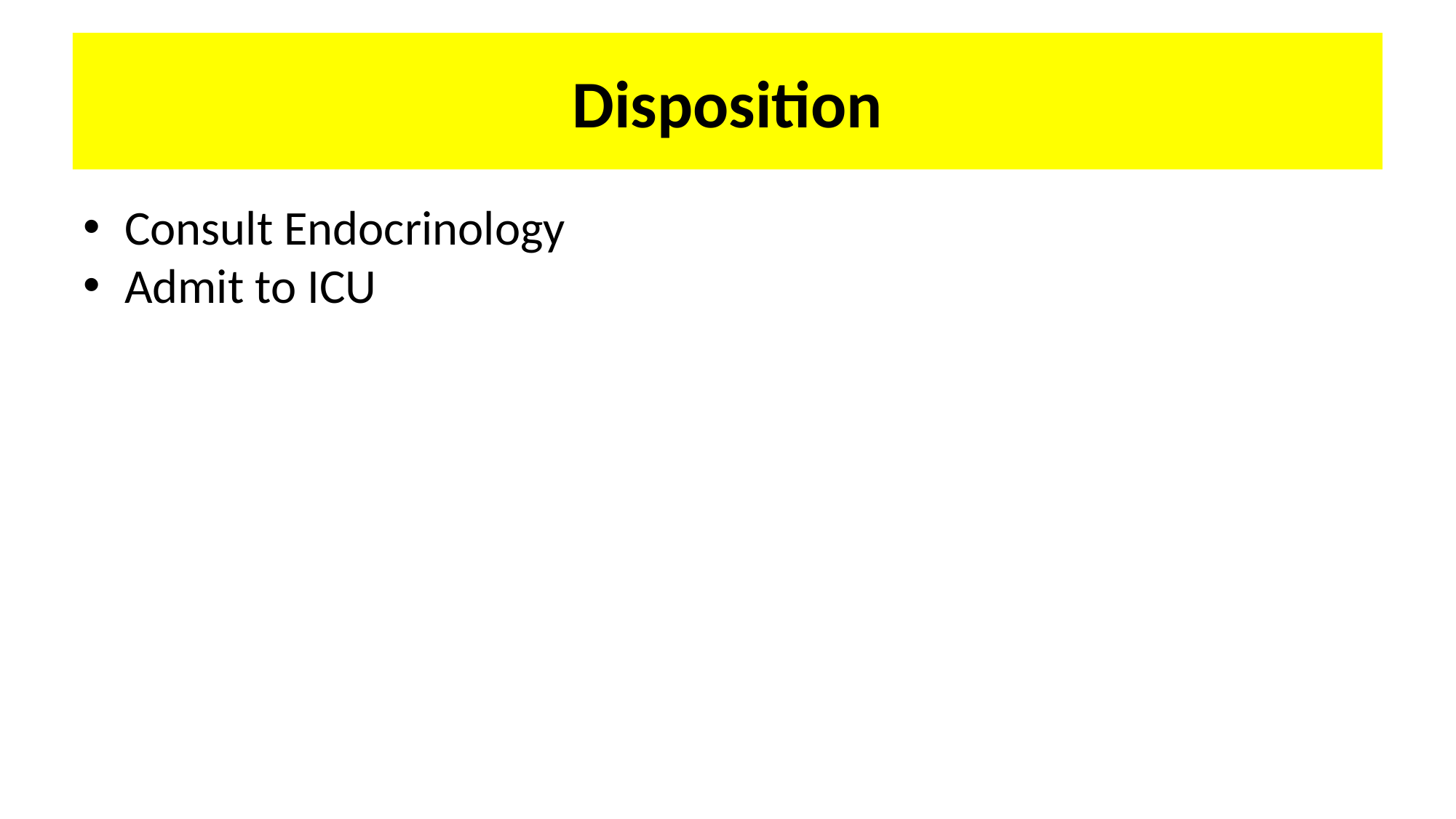

# Disposition
Consult Endocrinology
Admit to ICU

## Slide 38
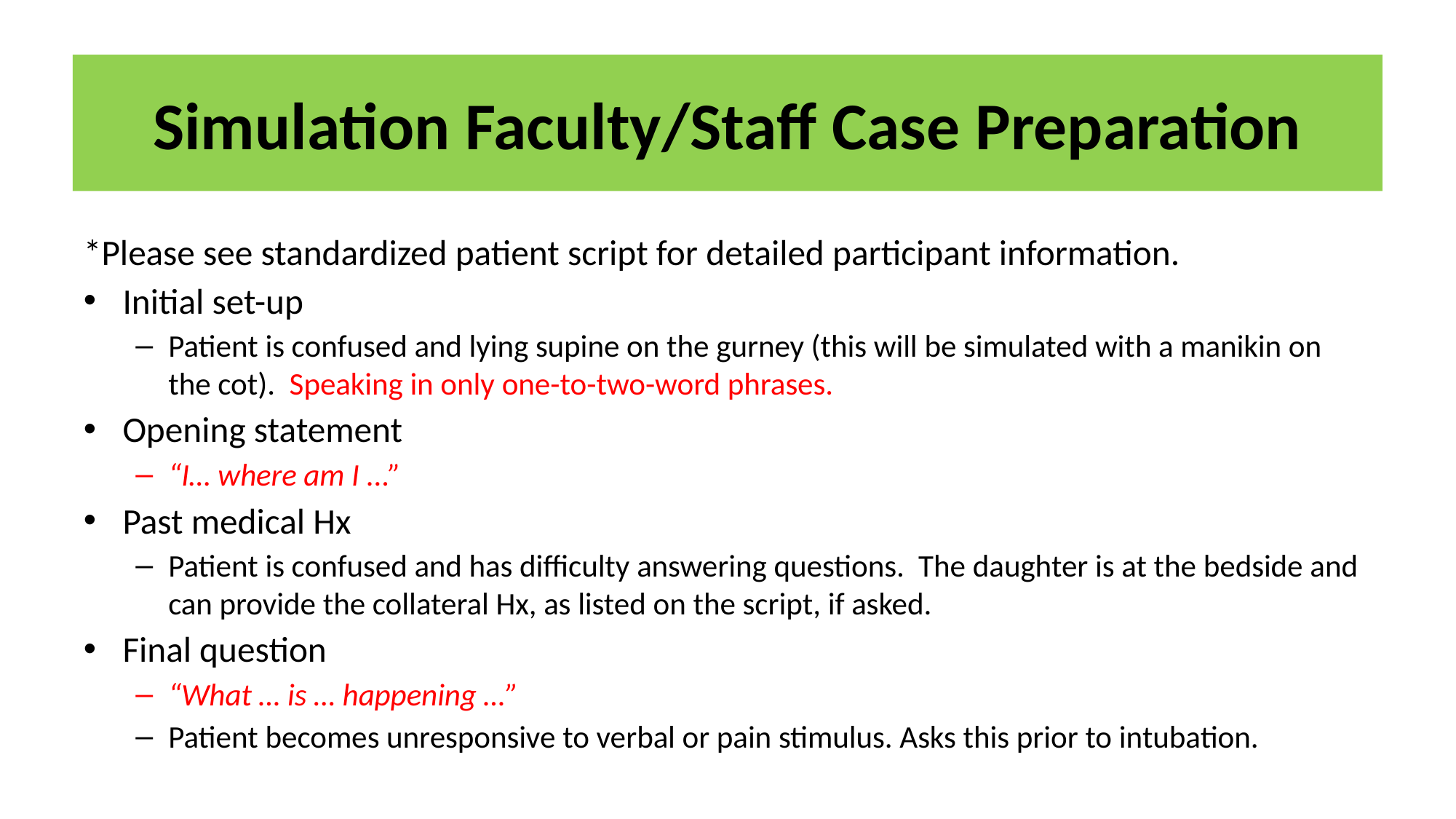

# Simulation Faculty/Staff Case Preparation
*Please see standardized patient script for detailed participant information.
Initial set-up
Patient is confused and lying supine on the gurney (this will be simulated with a manikin on the cot). Speaking in only one-to-two-word phrases.
Opening statement
“I… where am I ...”
Past medical Hx
Patient is confused and has difficulty answering questions. The daughter is at the bedside and can provide the collateral Hx, as listed on the script, if asked.
Final question
“What … is … happening …”
Patient becomes unresponsive to verbal or pain stimulus. Asks this prior to intubation.

## Slide 39
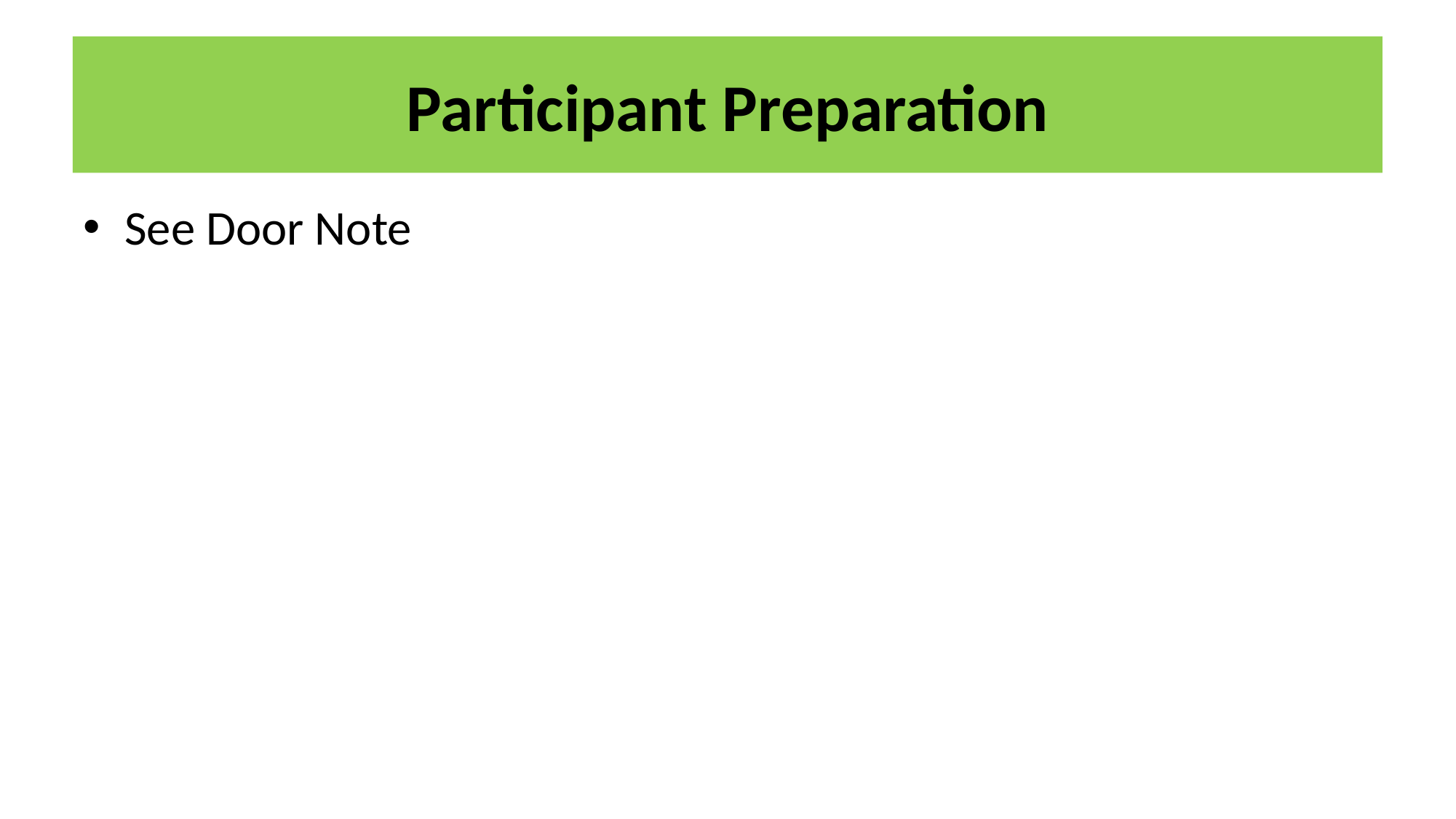

# Participant Preparation
See Door Note

## Slide 40
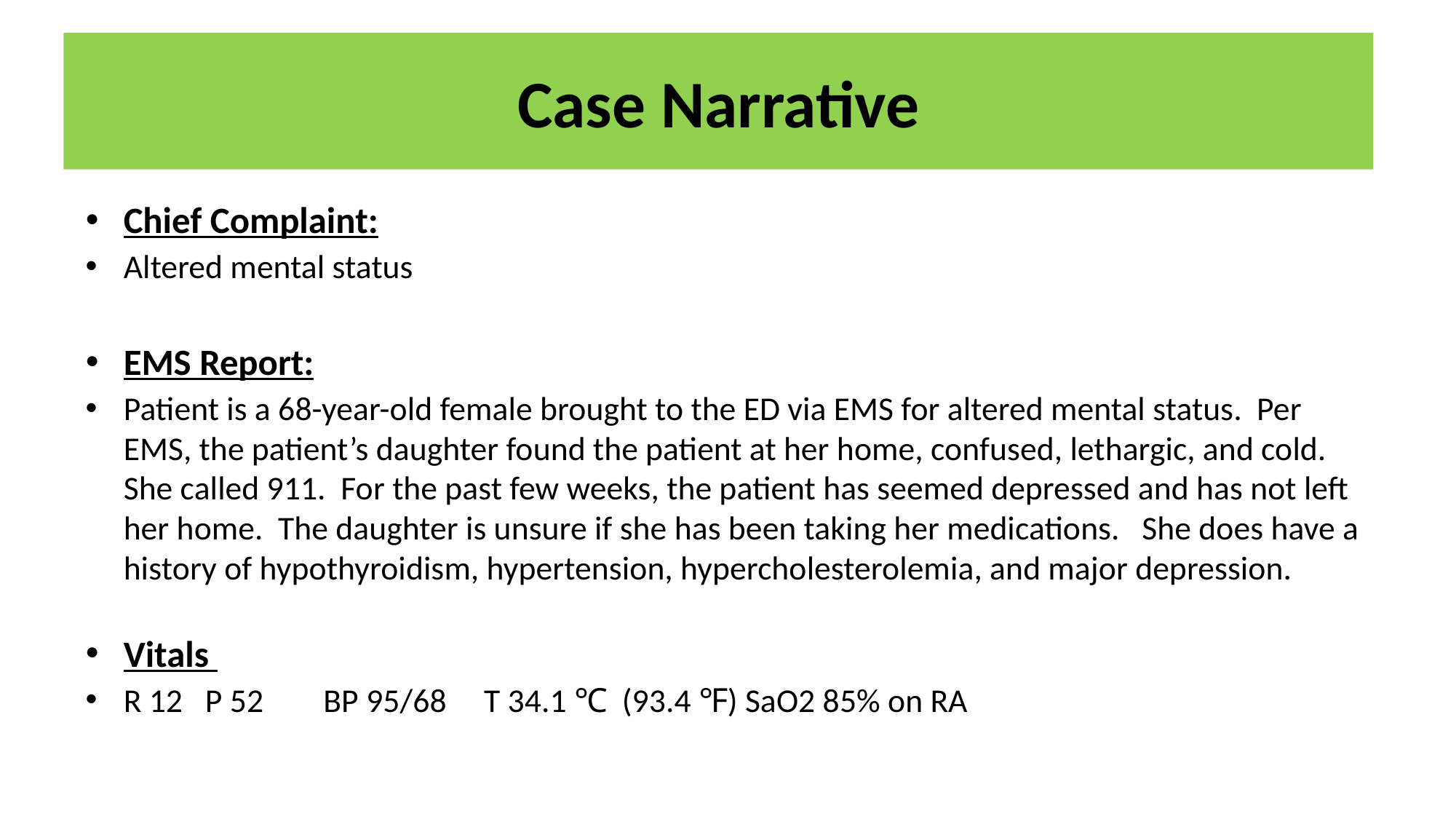

Case Narrative
#
Chief Complaint:
Altered mental status
EMS Report:
Patient is a 68-year-old female brought to the ED via EMS for altered mental status. Per EMS, the patient’s daughter found the patient at her home, confused, lethargic, and cold. She called 911. For the past few weeks, the patient has seemed depressed and has not left her home. The daughter is unsure if she has been taking her medications. She does have a history of hypothyroidism, hypertension, hypercholesterolemia, and major depression.
Vitals
R 12	 P 52	BP 95/68 T 34.1 ℃ (93.4 ℉) SaO2 85% on RA

## Slide 41
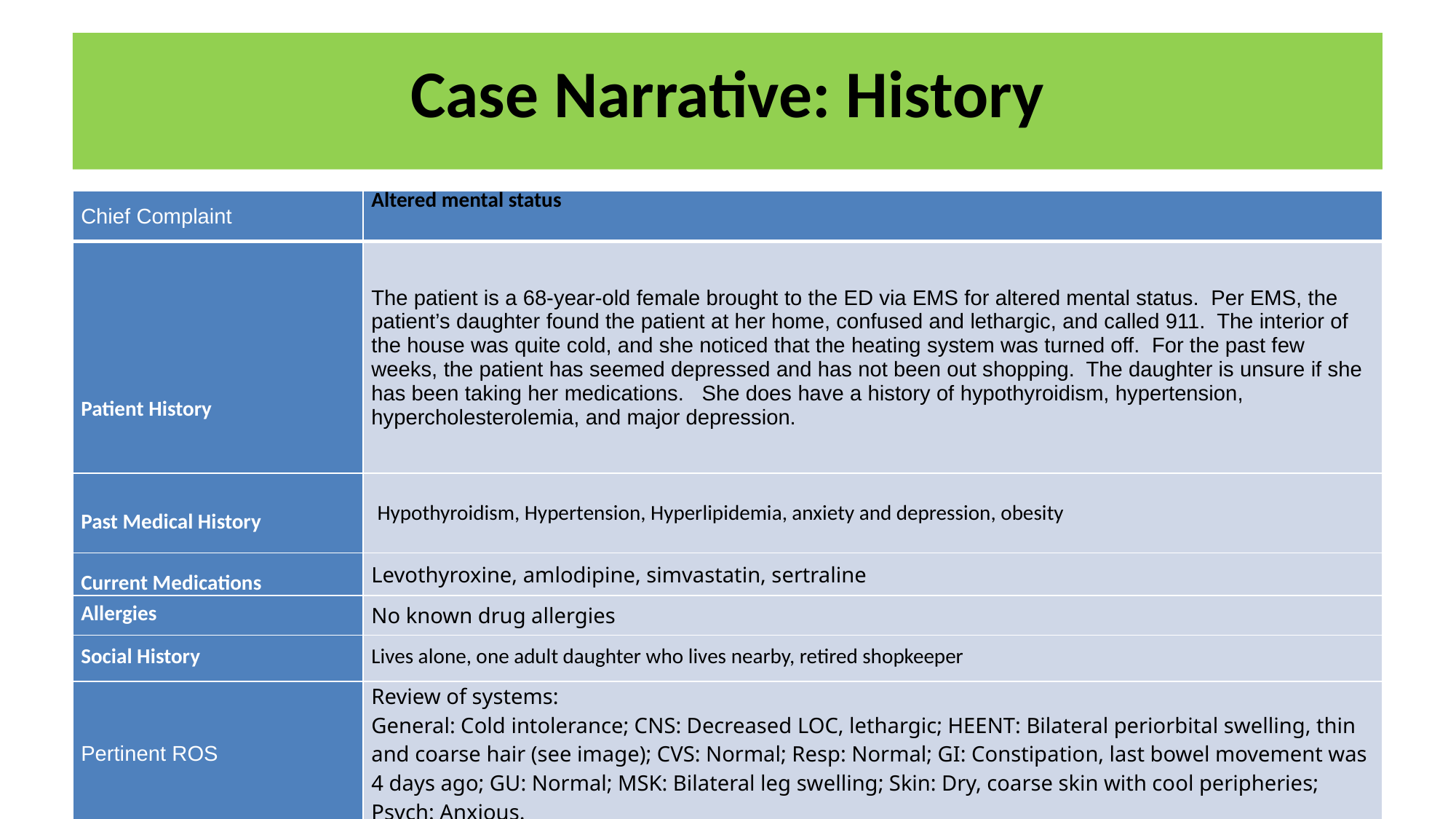

# Case Narrative: History
| Chief Complaint | Altered mental status |
| --- | --- |
| Patient History | The patient is a 68-year-old female brought to the ED via EMS for altered mental status. Per EMS, the patient’s daughter found the patient at her home, confused and lethargic, and called 911. The interior of the house was quite cold, and she noticed that the heating system was turned off. For the past few weeks, the patient has seemed depressed and has not been out shopping. The daughter is unsure if she has been taking her medications. She does have a history of hypothyroidism, hypertension, hypercholesterolemia, and major depression. |
| Past Medical History | Hypothyroidism, Hypertension, Hyperlipidemia, anxiety and depression, obesity |
| Current Medications | Levothyroxine, amlodipine, simvastatin, sertraline |
| Allergies | No known drug allergies |
| Social History | Lives alone, one adult daughter who lives nearby, retired shopkeeper |
| Pertinent ROS | Review of systems: General: Cold intolerance; CNS: Decreased LOC, lethargic; HEENT: Bilateral periorbital swelling, thin and coarse hair (see image); CVS: Normal; Resp: Normal; GI: Constipation, last bowel movement was 4 days ago; GU: Normal; MSK: Bilateral leg swelling; Skin: Dry, coarse skin with cool peripheries; Psych: Anxious. |

## Slide 42
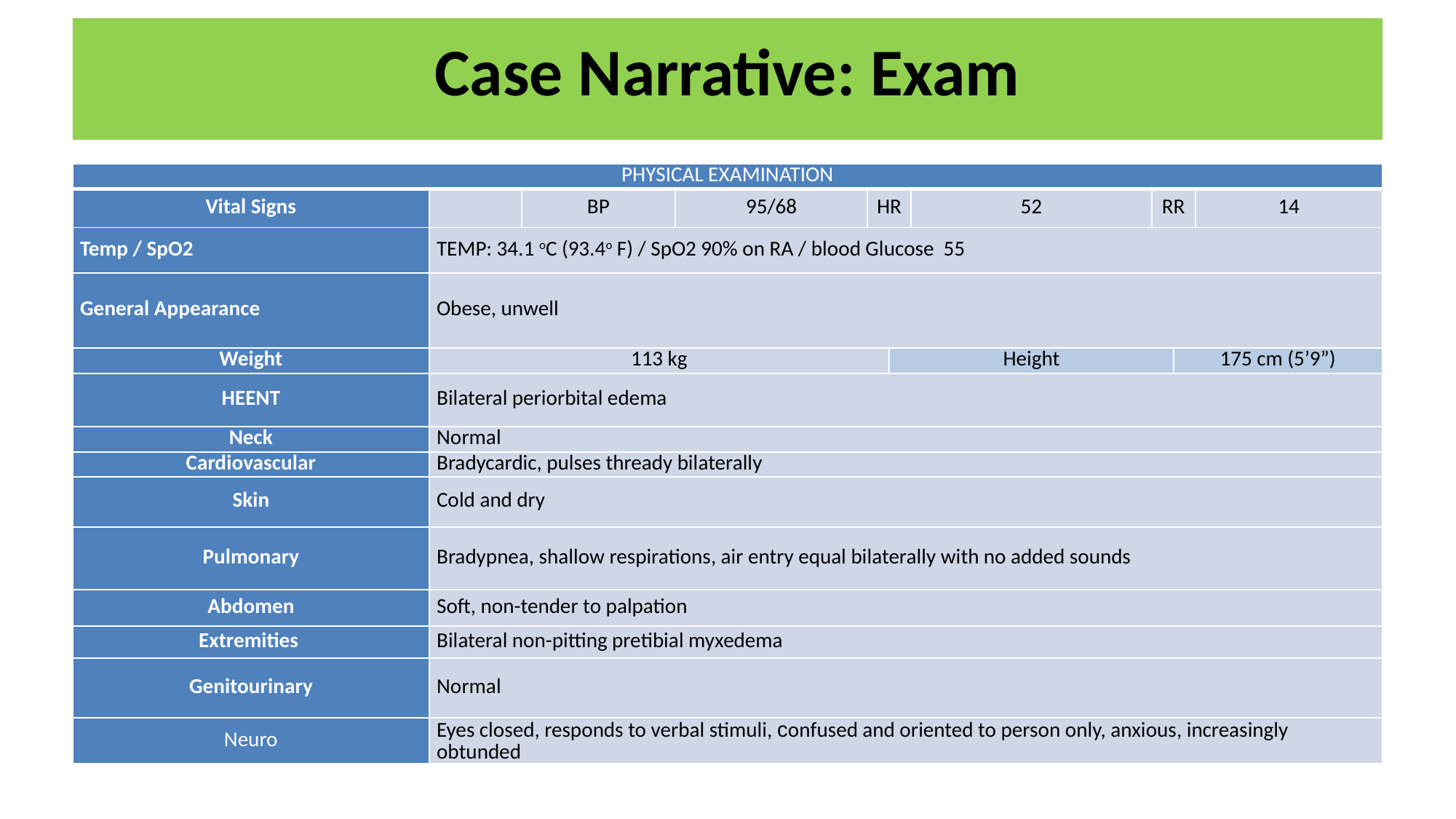

# Case Narrative: Exam
| PHYSICAL EXAMINATION | | | | | | | | | |
| --- | --- | --- | --- | --- | --- | --- | --- | --- | --- |
| Vital Signs | | BP | 95/68 | HR | | 52 | RR | | 14 |
| Temp / SpO2 | TEMP: 34.1 oC (93.4o F) / SpO2 90% on RA / blood Glucose 55 | | | | | | | | |
| General Appearance | Obese, unwell | | | | | | | | |
| Weight | 113 kg | | | | Height | | | 175 cm (5’9”) | |
| HEENT | Bilateral periorbital edema | | | | | | | | |
| Neck | Normal | | | | | | | | |
| Cardiovascular | Bradycardic, pulses thready bilaterally | | | | | | | | |
| Skin | Cold and dry | | | | | | | | |
| Pulmonary | Bradypnea, shallow respirations, air entry equal bilaterally with no added sounds | | | | | | | | |
| Abdomen | Soft, non-tender to palpation | | | | | | | | |
| Extremities | Bilateral non-pitting pretibial myxedema | | | | | | | | |
| Genitourinary | Normal | | | | | | | | |
| Neuro | Eyes closed, responds to verbal stimuli, confused and oriented to person only, anxious, increasingly obtunded | | | | | | | | |

## Slide 43
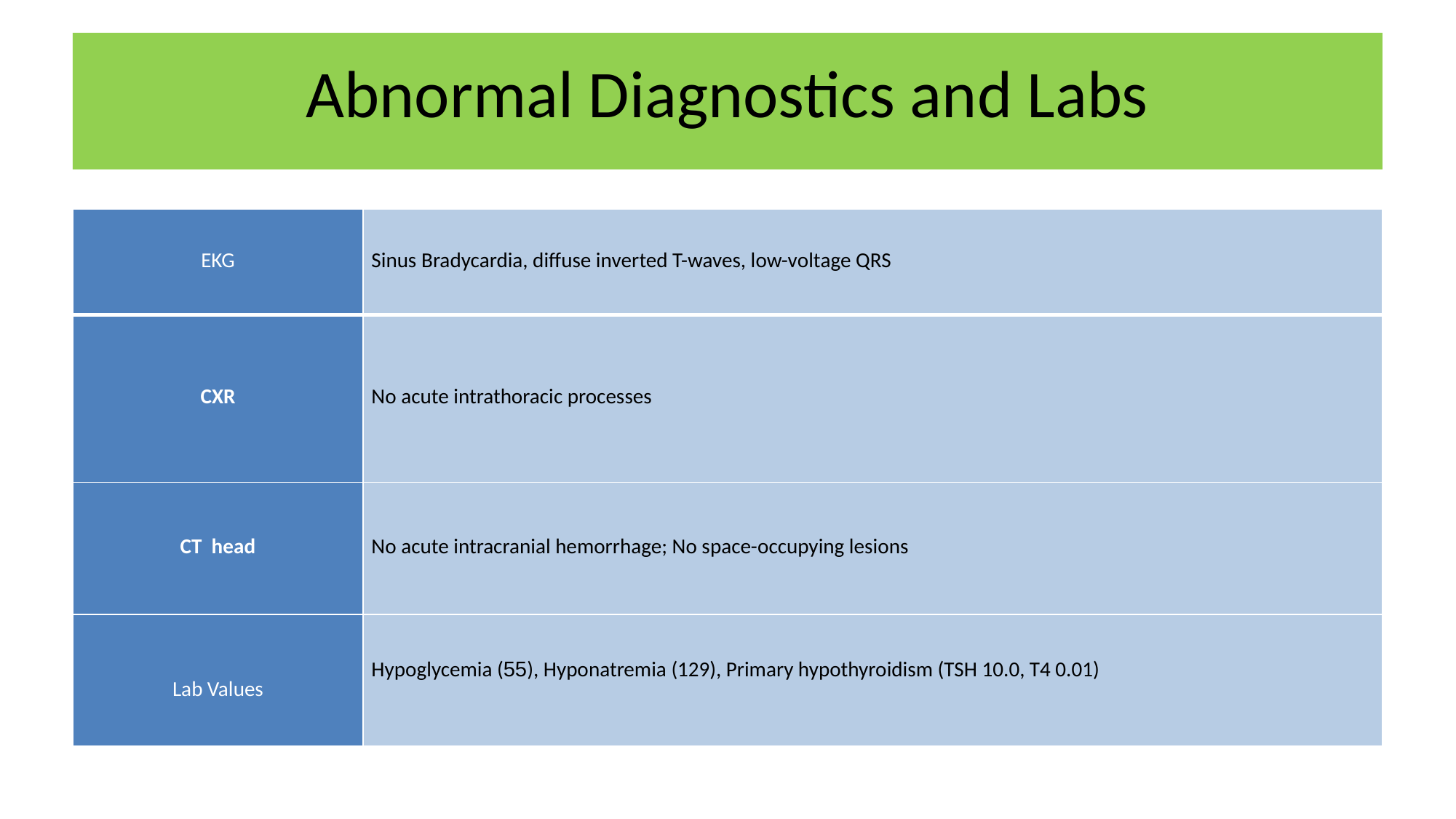

# Abnormal Diagnostics and Labs
| EKG | Sinus Bradycardia, diffuse inverted T-waves, low-voltage QRS |
| --- | --- |
| CXR | No acute intrathoracic processes |
| CT head | No acute intracranial hemorrhage; No space-occupying lesions |
| Lab Values | Hypoglycemia (55), Hyponatremia (129), Primary hypothyroidism (TSH 10.0, T4 0.01) |

## Slide 44
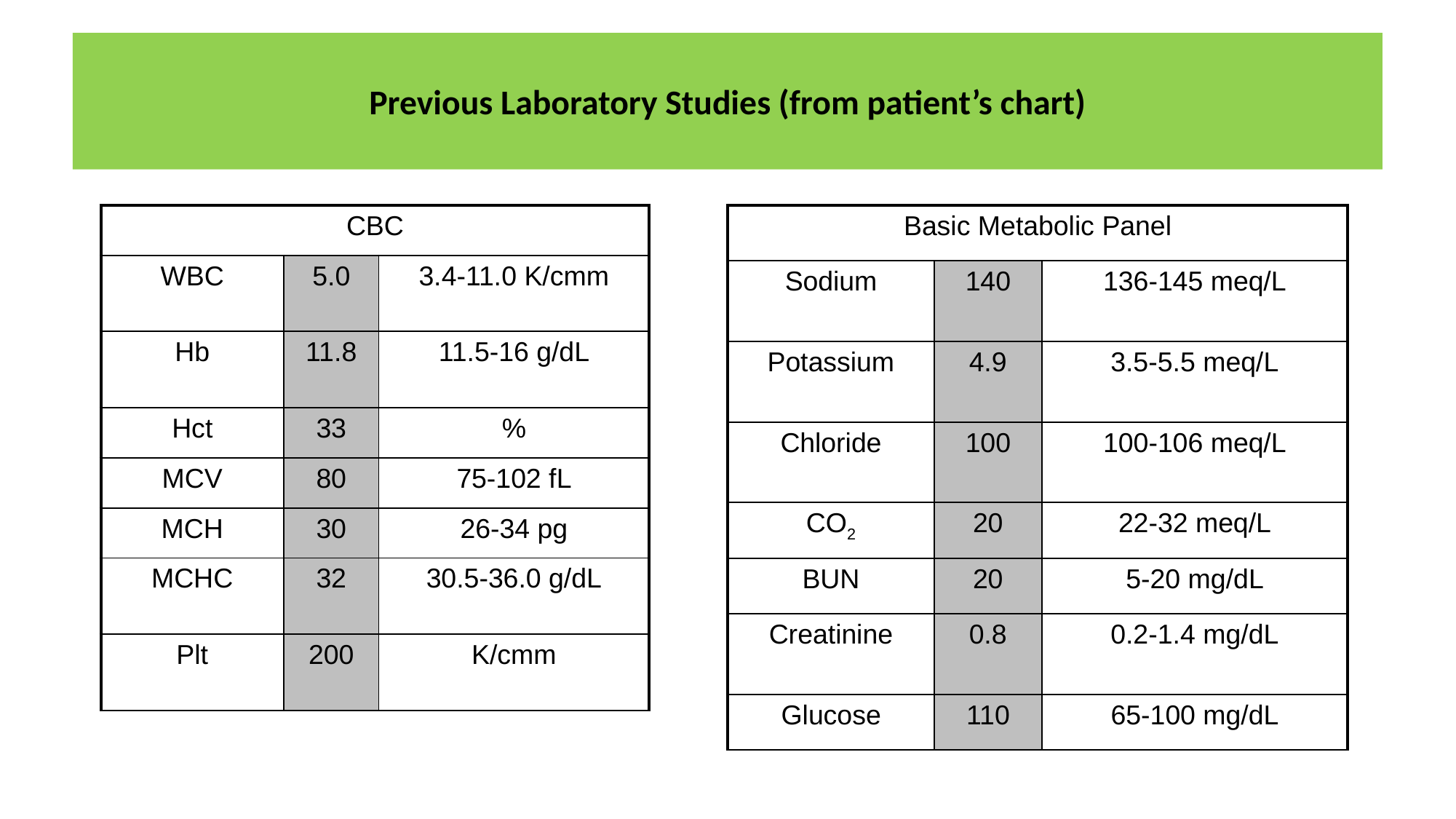

# Previous Laboratory Studies (from patient’s chart)
| CBC | | |
| --- | --- | --- |
| WBC | 5.0 | 3.4-11.0 K/cmm |
| Hb | 11.8 | 11.5-16 g/dL |
| Hct | 33 | % |
| MCV | 80 | 75-102 fL |
| MCH | 30 | 26-34 pg |
| MCHC | 32 | 30.5-36.0 g/dL |
| Plt | 200 | K/cmm |
| Basic Metabolic Panel | | |
| --- | --- | --- |
| Sodium | 140 | 136-145 meq/L |
| Potassium | 4.9 | 3.5-5.5 meq/L |
| Chloride | 100 | 100-106 meq/L |
| CO2 | 20 | 22-32 meq/L |
| BUN | 20 | 5-20 mg/dL |
| Creatinine | 0.8 | 0.2-1.4 mg/dL |
| Glucose | 110 | 65-100 mg/dL |

## Slide 45
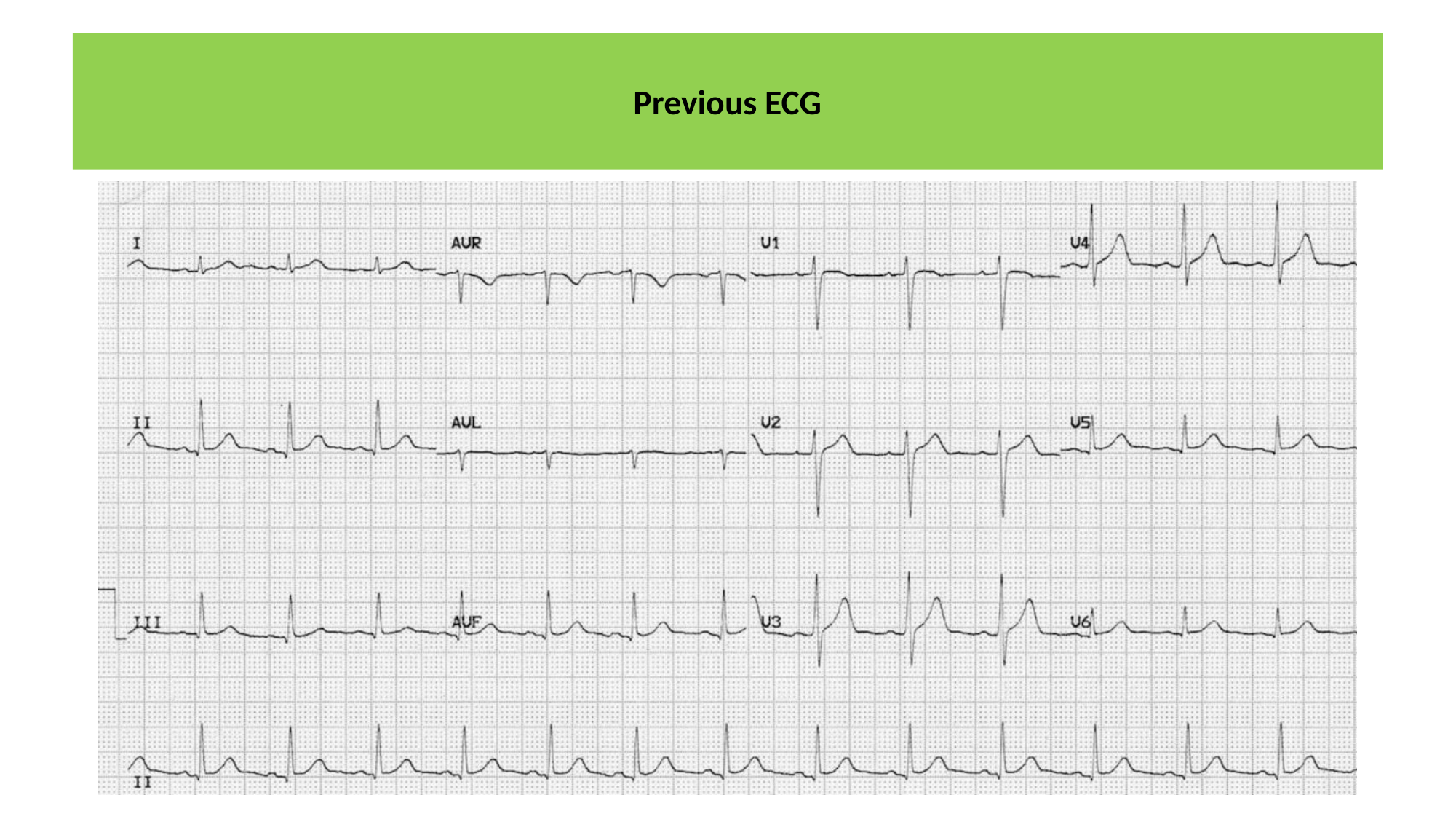

# Previous ECG

## Slide 46
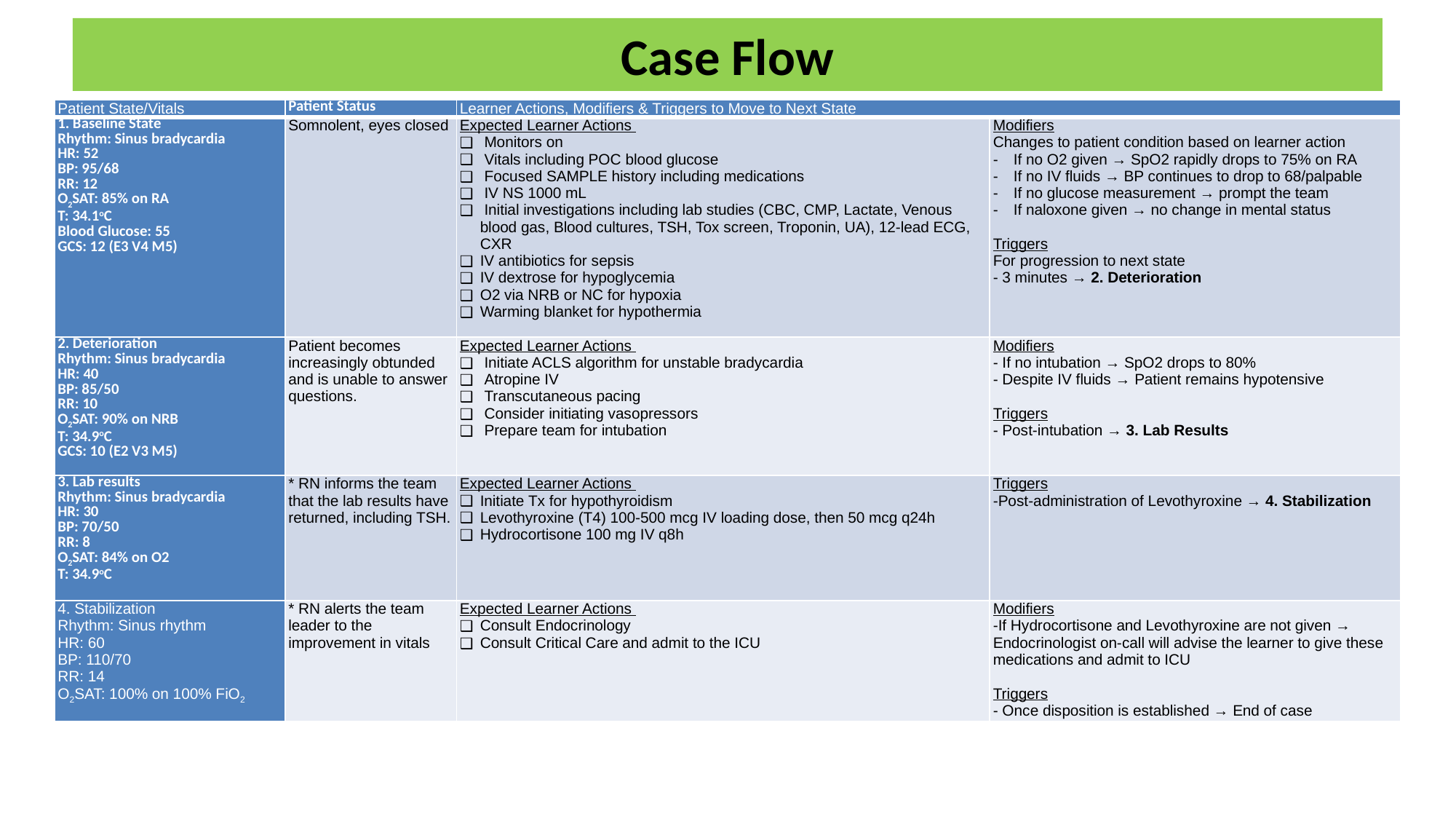

# Case Flow
| Patient State/Vitals | Patient Status | Learner Actions, Modifiers & Triggers to Move to Next State | |
| --- | --- | --- | --- |
| 1. Baseline State Rhythm: Sinus bradycardia HR: 52 BP: 95/68 RR: 12 O2SAT: 85% on RA T: 34.1oC Blood Glucose: 55 GCS: 12 (E3 V4 M5) | Somnolent, eyes closed | Expected Learner Actions Monitors on Vitals including POC blood glucose Focused SAMPLE history including medications IV NS 1000 mL Initial investigations including lab studies (CBC, CMP, Lactate, Venous blood gas, Blood cultures, TSH, Tox screen, Troponin, UA), 12-lead ECG, CXR IV antibiotics for sepsis IV dextrose for hypoglycemia O2 via NRB or NC for hypoxia Warming blanket for hypothermia | Modifiers Changes to patient condition based on learner action If no O2 given → SpO2 rapidly drops to 75% on RA If no IV fluids → BP continues to drop to 68/palpable If no glucose measurement → prompt the team If naloxone given → no change in mental status   Triggers For progression to next state - 3 minutes → 2. Deterioration |
| 2. Deterioration Rhythm: Sinus bradycardia HR: 40 BP: 85/50 RR: 10 O2SAT: 90% on NRB T: 34.9oC GCS: 10 (E2 V3 M5) | Patient becomes increasingly obtunded and is unable to answer questions. | Expected Learner Actions Initiate ACLS algorithm for unstable bradycardia Atropine IV Transcutaneous pacing Consider initiating vasopressors Prepare team for intubation | Modifiers - If no intubation → SpO2 drops to 80% - Despite IV fluids → Patient remains hypotensive   Triggers - Post-intubation → 3. Lab Results |
| 3. Lab results Rhythm: Sinus bradycardia HR: 30 BP: 70/50 RR: 8 O2SAT: 84% on O2 T: 34.9oC | \* RN informs the team that the lab results have returned, including TSH. | Expected Learner Actions Initiate Tx for hypothyroidism Levothyroxine (T4) 100-500 mcg IV loading dose, then 50 mcg q24h Hydrocortisone 100 mg IV q8h | Triggers -Post-administration of Levothyroxine → 4. Stabilization |
| 4. Stabilization Rhythm: Sinus rhythm HR: 60 BP: 110/70 RR: 14 O2SAT: 100% on 100% FiO2 | \* RN alerts the team leader to the improvement in vitals | Expected Learner Actions Consult Endocrinology Consult Critical Care and admit to the ICU | Modifiers -If Hydrocortisone and Levothyroxine are not given → Endocrinologist on-call will advise the learner to give these medications and admit to ICU   Triggers - Once disposition is established → End of case |

## Slide 47
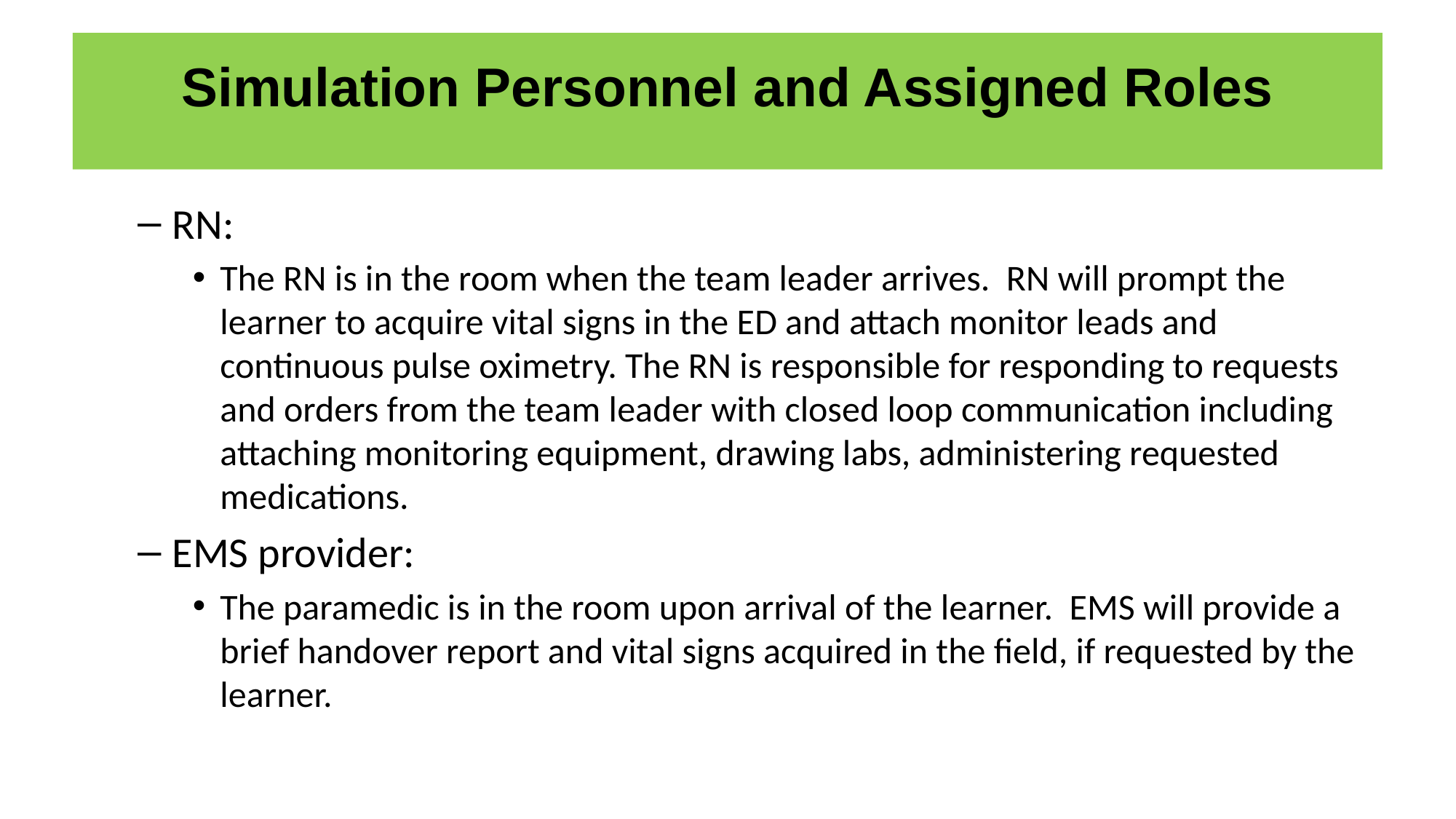

# Simulation Personnel and Assigned Roles
RN:
The RN is in the room when the team leader arrives. RN will prompt the learner to acquire vital signs in the ED and attach monitor leads and continuous pulse oximetry. The RN is responsible for responding to requests and orders from the team leader with closed loop communication including attaching monitoring equipment, drawing labs, administering requested medications.
EMS provider:
The paramedic is in the room upon arrival of the learner. EMS will provide a brief handover report and vital signs acquired in the field, if requested by the learner.

## Slide 48
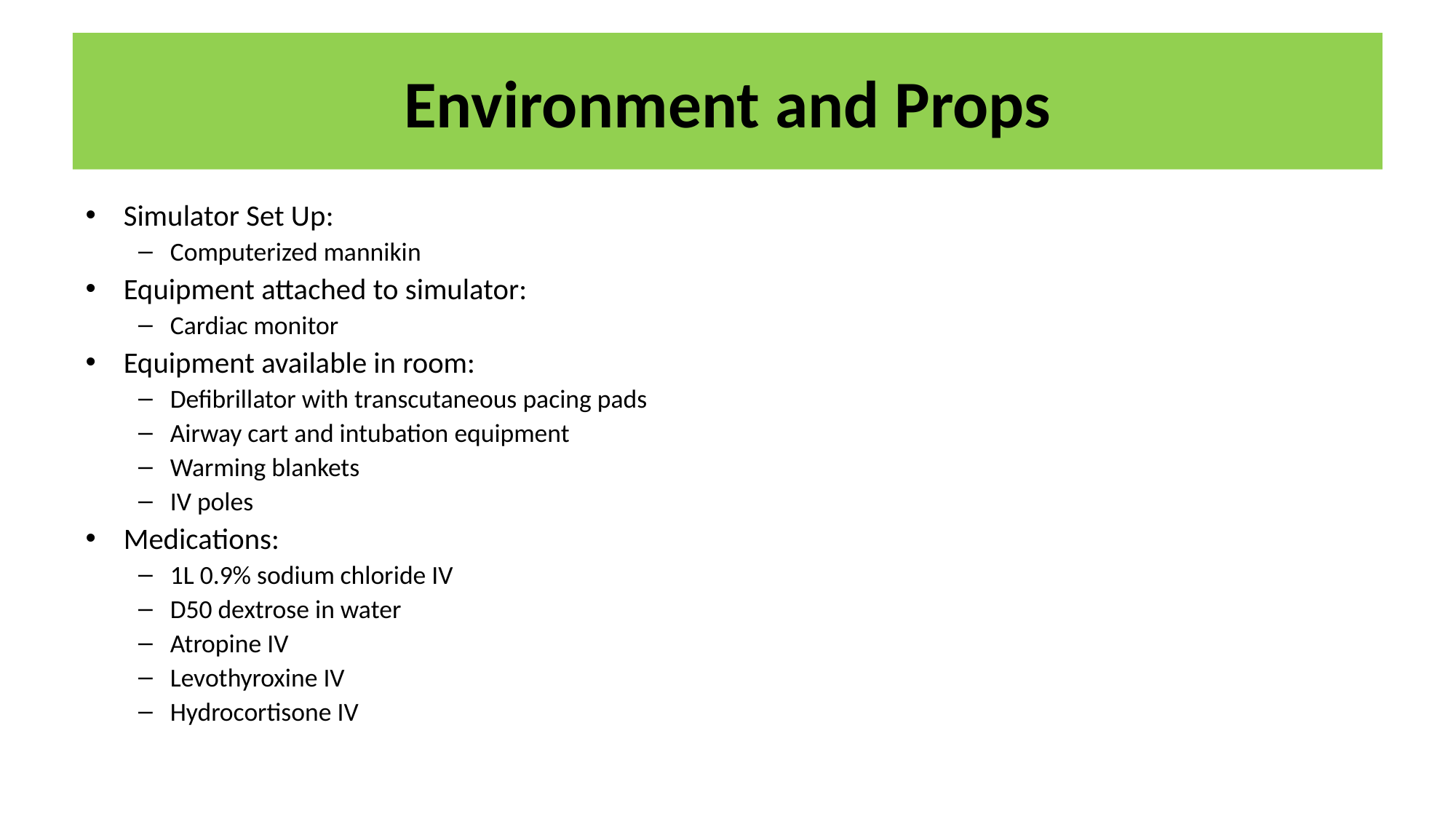

# Environment and Props
Simulator Set Up:
Computerized mannikin
Equipment attached to simulator:
Cardiac monitor
Equipment available in room:
Defibrillator with transcutaneous pacing pads
Airway cart and intubation equipment
Warming blankets
IV poles
Medications:
1L 0.9% sodium chloride IV
D50 dextrose in water
Atropine IV
Levothyroxine IV
Hydrocortisone IV

## Slide 49
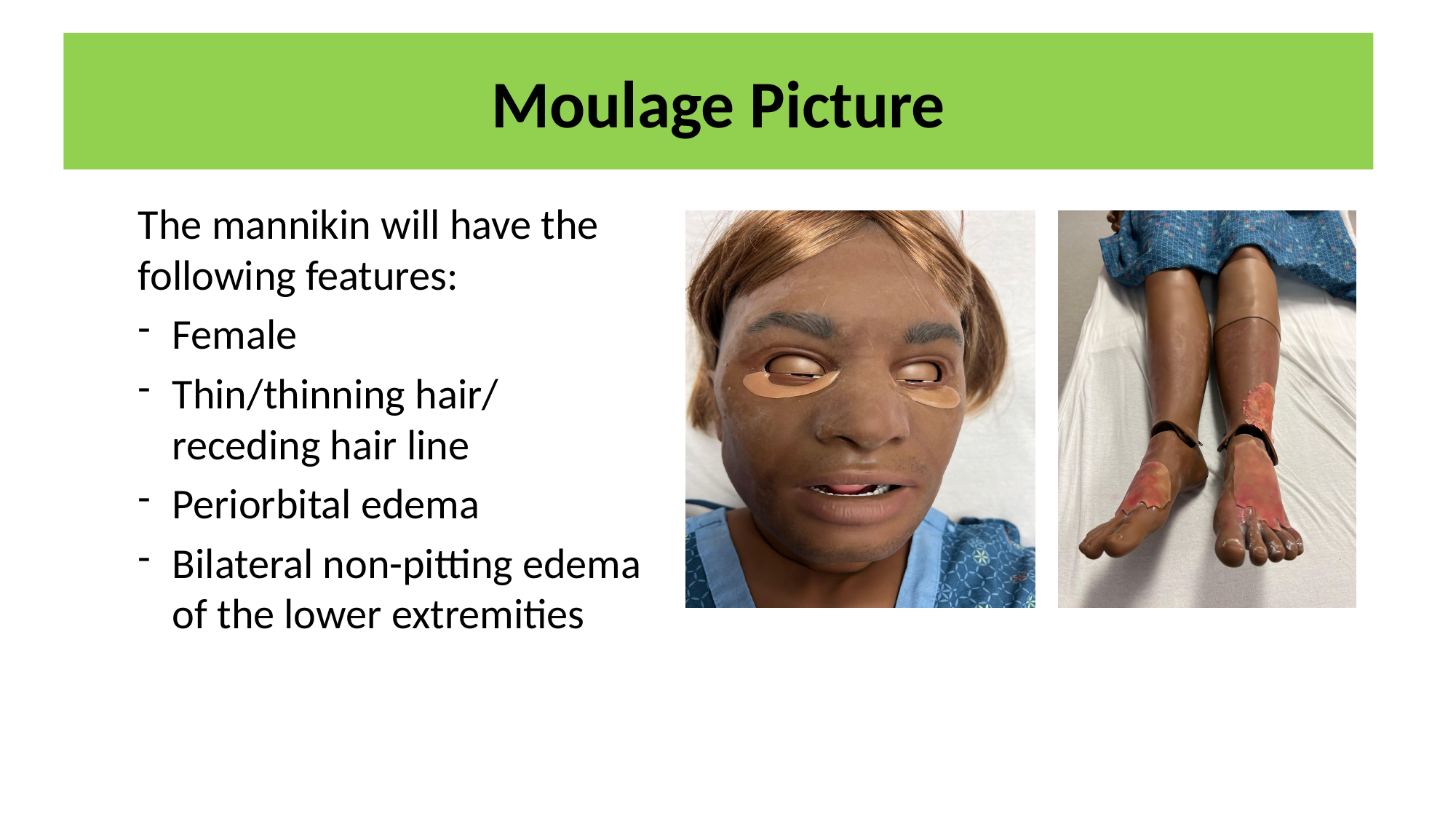

Moulage Picture
#
The mannikin will have the following features:
Female
Thin/thinning hair/ receding hair line
Periorbital edema
Bilateral non-pitting edema of the lower extremities

## Slide 50
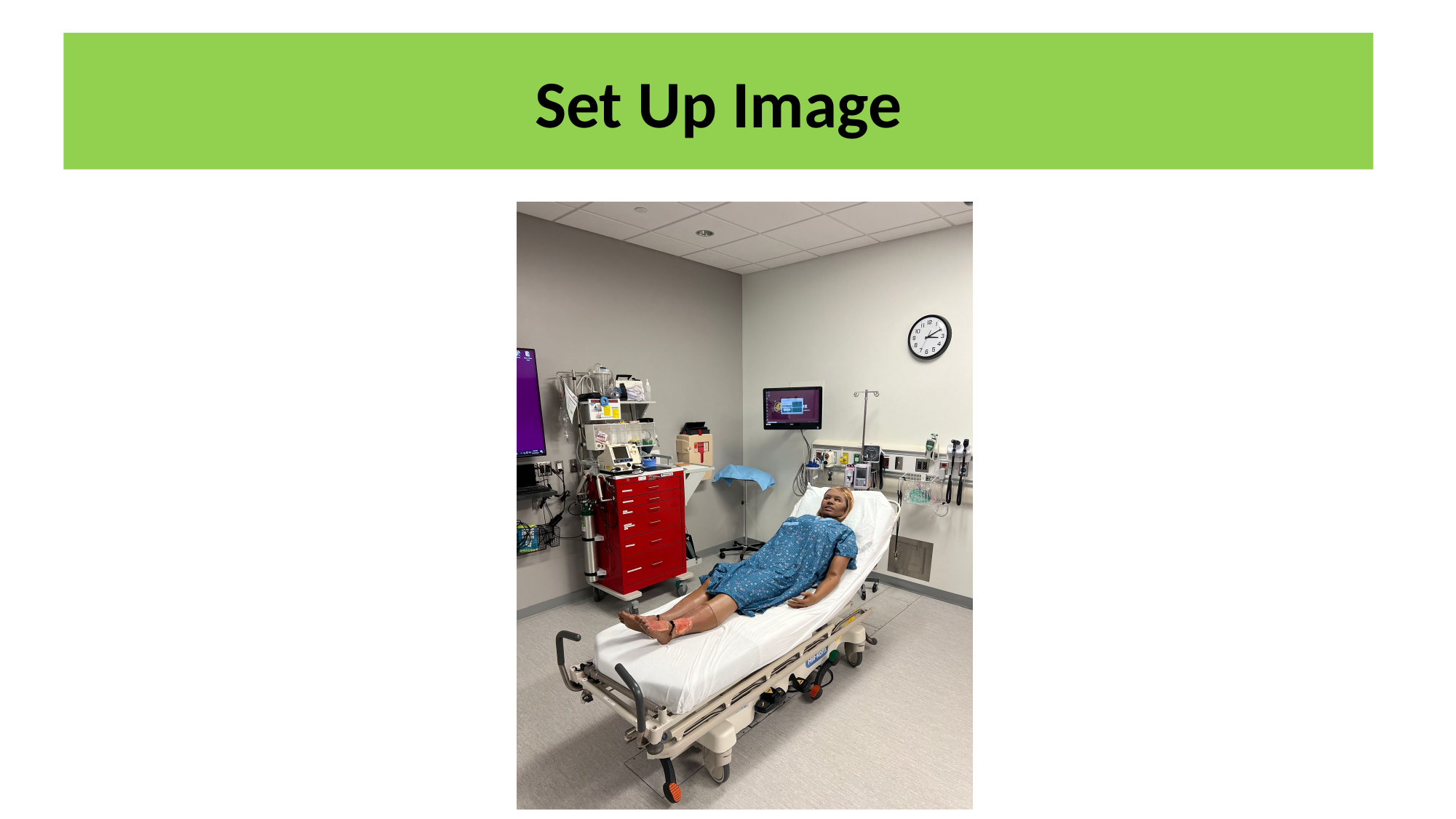

Set Up Image
#
